# Chemical Profiling and Differential Constituents Analysis of Honghema and Its Easily Confusable Species Driven by High-Resolution Mass Spectrometry and Pattern Recognition

**DOI:** 10.3390/molecules31152614

**Published:** 2026-07-27

**Authors:** Congying Huang, Yute Zhong, Han Li, Dan Wu, Taiping Li, Weijie Li, Siying Chen, Zipeng Gong, Haiyu Xu, Ping Wang

**Affiliations:** 1 State Key Laboratory for Quality Ensurance and Sustainable Use of Dao-di Herbs, Institute of Chinese Materia Medica China Academy of Chinese Medical Sciences, Beijing 100700, China; 2 Graduate School of China Academy of Chinese Meical Sciences, Suzhou 215105, China; 3 Liaoning University of Traditional Chinese Medicine, Shenyang 117004, China; 4 State Key Laboratory of Discovery and Utilization of Functional Components in Traditional Chinese Medicine, Engineering Research Center for the Development and Application of Ethnic Medicine and TCM (Ministry of Education), School of Pharmacy, Guizhou Medical University, Guian New Area 561113, China; 5 Guizhou Key Laboratory of Modern Traditional Chinese Medicine Creation, Guiyang 550004, China

**Keywords:** Honghema, *Laportea bulbifera* (Siebold &amp, Zucc.) Wedd., *Girardinia diversifolia* (Link) Friis, *Urtica mairei* H. Lév., UPLC-Q-TOF-MS/MS, chemical profiling, differential constituents

## Abstract

Background: Honghema is a traditional Chinese medicinal material and is also widely used by ethnic minority communities in Guizhou Province, where it is commonly known as “Reib ndad gunb” and “Uab Detdend”. The botanical origins were the whole plants of *Laportea bulbifera* (Siebold & Zucc.) Wedd. (*L. bulbifera*) and *Girardinia diversifolia* (Link) Friis (*G. diversifolia*). Due to morphological similarities, *Urtica mairei* H. Lév. (*U. mairei*) was often mixed as easily confusable species in actual market circulation. However, systematic research regarding the differences in chemical composition between the two botanical origins, as well as between them and their easily confusable species remain limited. This study aimed to comprehensively characterise the chemical constituents of *L. bulbifera*, *G. diversifolia*, and *U. mairei*, and to identify significantly differential chemical constituents for their discrimination. Methods: Data acquisition was performed using ultra-performance liquid chromatography–mass spectrometry (UPLC-MS). Multivariate statistical analyses combined with reference standard comparison were performed to investigate the differences in chemical profiles among *L. bulbifera*, *G. diversifolia* and *U. mairei*, identify the key differential constituents leading to the differences, and clarify their relative abundance information. Results: A total of 133, 77, and 59 chemical constituents were identified from *L. bulbifera*, *G. diversifolia*, and *U. mairei*, respectively. These compounds comprised flavonoids, organic acids, carbohydrates, organonitrogen compounds, terpenoids, coumarins, lipids, steroids, and other types of compounds. Flavonoids constituted the highest proportion of the compounds in *L. bulbifera*, whereas organic acids were the most abundant in *G. diversifolia* and *U. mairei*. Through multivariate statistical analysis, 32 inter-group differential components between *L. bulbifera* and *G. diversifolia*, 27 between *L. bulbifera* and *U. mairei*, and 13 between *G. diversifolia* and *U. mairei* were screened and identified. The core components contributing to these differences included Baimaside, Roxburic acid, 1-Galloyl-glucose, Protocatechuic acid 3-glucoside, and Schaftoside. Venn diagram analysis revealed 20 characteristic differential constituents of *L. bulbifera*, mainly represented by flavonoids; 6 characteristic differential constituents of *G. diversifolia*, primarily comprising terpenoids and organic acids; and 4 characteristic differential constituents of *U. mairei*, mainly represented by carbohydrates. Conclusion: Significant differences were observed among the chemical profiles of the three plant species. The method established in this study can effectively identify the chemical compositions of Honghema and its easily confusable species, revealing the key components responsible for the variations in their chemical profiles. This study provides a basis for elucidating the material foundation of Honghema and offers an effective analytical approach and valuable reference data for distinguishing Honghema from its easily confusable species.

## 1. Introduction

Honghema is a commonly used plant medicine in China, and a traditional medicine utilised by ethnic minorities in Guizhou Province. In 2022, it was included in the *Catalogue of Miao and other ethnic minority medicinal materials in Guizhou Province* [1]. The *Quality Standards for Chinese Medicinal Materials and Ethnic Medicinal Materials of Guizhou Province* (2019 edition) [2] stipulated that the botanical origins of Honghema were the whole plants of *L. bulbifera* and *G. diversifolia*. Honghema is often used alone or in combination with other herbal medicines for the treatment of rheumatoid arthritis (RA) and other diseases, demonstrating considerable medicinal value. Several compound preparations containing Honghema as the principal medicinal ingredient have been included in the Chinese national drug standards, including Runzao Zhiyang Capsules, Compound Shangfuning Ointment, Liuwei Shangfuning Tincture, and Tongluo Guzhining Ointment. Pharmacological studies have demonstrated that *Honghema* could effectively alleviate pain and inhibit excessive lymphocyte proliferation [3,4,5], reduce joint swelling and bone destruction [6,7], and induce apoptosis of pathological synovial cells by activating the mitochondrial apoptotic pathway [8], exhibiting significant anti-inflammatory and analgesic properties [9,10,11]. Previous chemical investigations of Honghema have mainly focused on *L. bulbifera*, which contains abundant flavonoids, phenolic acids, steroids, terpenoids, and others. Multiple constituents have been identified from different parts of this plant by researchers. Feng et al. studied the stems, leaves and roots of *L. bulbifera* and found phenolic and fatty acid components [12]. Lu et al. isolated 17 chemical constituents from the stems and leaves of *L. bulbifera* [13]. Tang et al. analyzed the extract of *L. bulbifera* and identified 6 flavonoids and 8 other components [14]. Furthermore, Shrestha et al. conducted a phytochemical analysis on the extract of *G. diversifolia* [15], identifying steroids, fatty acids, and phenolics. Zhang et al. investigated the aerial parts of *G. diversifolia* and isolated five chemical constituents [16]. Liu et al. extracted and isolated 20 chemical components from *G. diversifolia* [17], while Liu et al. isolated and identified three compounds [18]. Although these studies have made significant contributions to elucidating the chemical profile of Honghema, systematic analyses of its constituents remain insufficient, and the types, contents, and distribution characteristics of its specific marker compounds have yet to be fully clarified. The accurate identification of botanical origins and the consistency of medicinal material quality directly affect clinical efficacy and medication safety. Therefore, further chemical investigations of Honghema are required to expand the discovery of novel chemical constituents, identify differential chemical markers, which will subsequently facilitate a comprehensive understanding of the material basis underlying its medicinal effects.

On the other hand, during practical circulation and application, Honghema is commonly subjected to preliminary processing, such as coarse cutting before medicinal use. This processing results in the near-complete loss of its macroscopic morphological characteristics, thereby exacerbating the difficulty of species identification and leading to the occurrence of substitution or admixture with easily confusable species. For instance, *U. mairei*, a species belonging to the same family, is frequently used as an easily confusable species of Honghema due to its similar morphological appearance and has become one of the typical confused species encountered during commercial circulation. Regarding the current status of phytochemical research, existing reports predominantly focus on *L. bulbifera*, whereas only a limited number of studies have involved *G. diversifolia*. Furthermore, no studies have concurrently examined Honghema alongside easily confusable species. Although *L. bulbifera* and *G. diversifolia* are both recognised as botanical origins of Honghema, whether their chemical compositions are similar and whether their core pharmacologically active constituents are consistent remain unclear. Meanwhile, it is yet to be determined whether significant chemical variations exist between Honghema and its easily confusable species, and whether they can be effectively differentiated via chemical methodologies. The current absence of systematic, parallel comparative studies significantly increases the difficulty of quality control (QC) for Honghema. Therefore, analysing the differences between Honghema and its easily confusable species from a phytochemical perspective, as well as establishing a rapid and reliable method for the discovery of chemical markers and the systematic identification and discrimination of these species, constitutes a crucial approach to resolving this issue.

Honghema possesses significant medicinal value, but reliable identification methods are required for its QC. In this study, a systematic chemical analysis was conducted on the two botanical origins of Honghema (*L. bulbifera* and *G. diversifolia*) and its easily confusable species (*U. mairei*), using ultra-high-performance liquid chromatography coupled with quadrupole time-of-flight tandem mass spectrometry (UPLC-Q-TOF-MS/MS) coupled with pattern recognition methods. First, the chemical compositions of the three plant species were comprehensively characterised to elucidate the chemical profiles. Subsequently, comparative analyses were performed to investigate the differences in chemical compositions between *L. bulbifera* and *G. diversifolia*, *L. bulbifera* and *U. mairei*, as well as *G. diversifolia* and *U. mairei*. The relative abundance variations of key constituents were evaluated, and potential chemical marker compounds were screened for species discrimination. The findings of this study provide a scientific basis for elucidating the material basis of Honghema and distinguishing it from commonly confused species. Furthermore, the results offer valuable data references for improving the quality standards and QC strategies of Honghema.

## 2. Results

### 2.1. Identification of Chemical Constituents in L. bulbifera, G. diversifolia, and U. mairei

**Figure 1:**
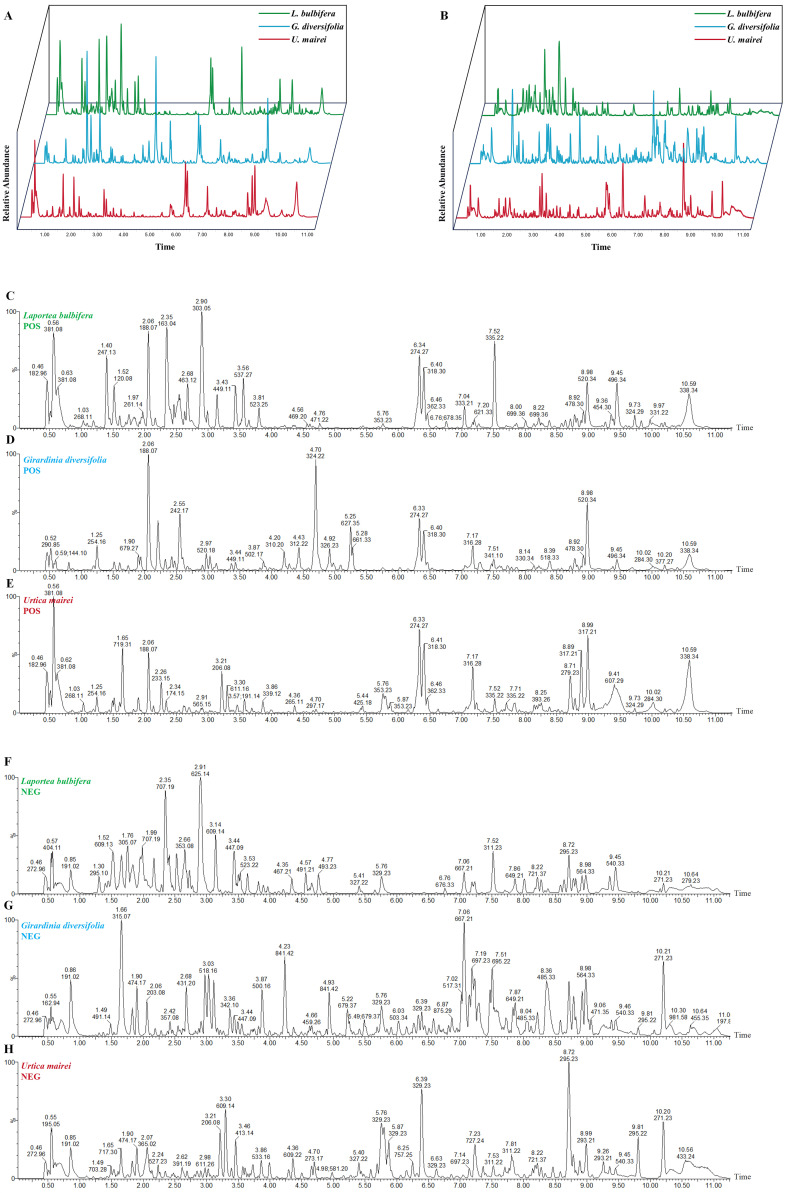
The based peak intensity (BPI) chromatograms of UPLC-Q-TOF/MS of *L. bulbifera*, *G. diversifolia*, and *U. mairei* in positive ion mode (**A**) and negative ion mode (**B**). The BPI chromatograms of *L. bulbifera* (**C**), *G. diversifolia* (**D**), and *U. mairei* (**E**) in positive ion mode. The BPI chromatograms of *L. bulbifera* (**F**), *G. diversifolia* (**G**), and *U. mairei* (**H**) in negative ion mode.

The reliability of the established UPLC-Q-TOF-MS/MS method was first evaluated using QC samples. The QC samples were prepared by mixing equal volumes of all sample extracts and were repeatedly injected at regular intervals throughout the analytical sequence. The reproducibility of the method was assessed by monitoring the stability of instrumental responses. The representative base peak intensity (BPI) chromatograms of the three plant species were shown in Figure 1A,B. Distinct differences were observed in the retention time distribution ranges and response intensities of the major chromatographic peaks among the three species, indicating that *L. bulbifera*, *G. diversifolia*, and *U. mairei* exhibited distinguishable overall chemical profiles. Based on accurate relative molecular masses, MS/MS fragmentation patterns of compounds, previously reported chemical constituents of three species, and an in-house database, the chemical constituents of the three species were systematically identified. The MS/MS spectra of reference standards were presented in Supplementary Figure S1.

**Figure 2:**
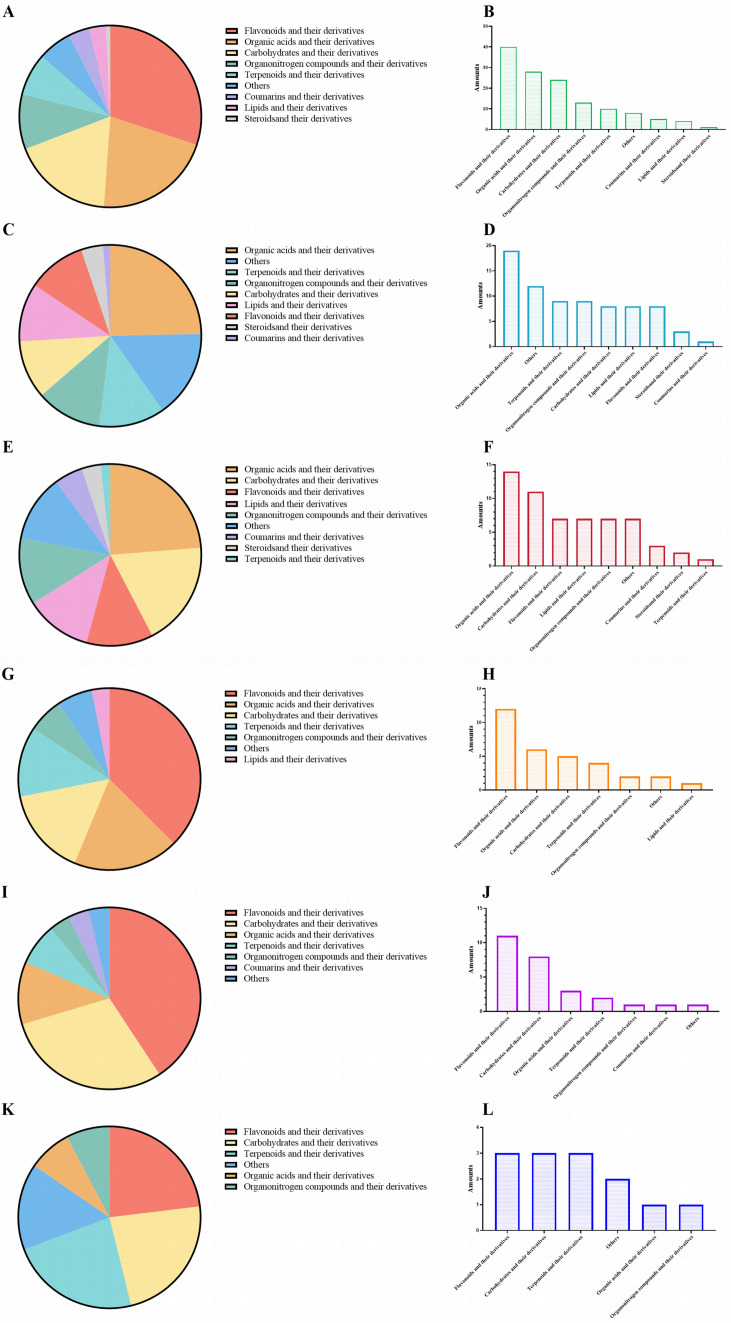
Statistics of the compounds of *L. bulbifera* (**A**,**B**), *G. diversifolia* (**C**,**D**), and *U. mairei* (**E**,**F**). Statistics of significantly differential components between *L. bulbifera* and *G. diversifolia* (**G**,**H**). Statistics of significantly differential components between *L. bulbifera* and *U. mairei* (**I**,**J**). Statistics of significantly differential components between *G. diversifolia* and *U. mairei* (**K**,**L**).

A total of 133 chemical constituents were characterised from *Laportea bulbifera*. According to their structural classifications, these constituents included 40 flavonoids, 28 organic acids, 24 carbohydrates, 13 organonitrogen compounds, 10 terpenoids, 5 coumarins, 4 lipids, 1 steroid, and 8 other constituents (Table 1; Figure 1C,F and Figure 2A,B). Flavonoids represented the predominant constituent class in *L. bulbifera* and exhibited high chromatographic responses. Organic acids and carbohydrates also showed relatively strong chromatographic responses and constituted important components of its chemical profile. A total of 77 chemical constituents were characterised from *G. diversifolia*, comprising 19 organic acids, 9 terpenoids, 9 organonitrogen compounds, 8 carbohydrates, 8 lipids, 8 flavonoids, 3 steroids, 1 coumarin, and 12 other constituents (Table 2; Figure 1D,G and Figure 2C,D). Organic acids, terpenoids and organonitrogen compounds exhibited high chromatographic responses in the chromatograms. Compared with *L. bulbifera*, flavonoids in *G. diversifolia* accounted for a smaller proportion of the total constituents. Lipids, carbohydrates and steroids constituted important classes within its chemical composition. A total of 59 chemical constituents were characterised from *U. mairei*, encompassing 14 organic acids, 11 carbohydrates, 7 flavonoids, 7 lipids, 7 organonitrogen compounds, 3 coumarins, 2 steroids, 1 terpenoid, and 7 other constituents (Table 3; Figure 1E,H and Figure 2E,F). Organic acids and carbohydrates represented the major constituent classes of *U. mairei*, and both the chromatographic response and the number of detected flavonoids were lower than those observed in *L. bulbifera* and *G. diversifolia*.

The identification of the chemical constituents in *L. bulbifera*, *G. diversifolia*, and *U. mairei* demonstrated that significant differences exist in the chemical composition characteristics and principal component categories among the three species.

#### 2.1.1. Characterization and Identification of Flavonoids and Their Derivatives

**Figure 3:**
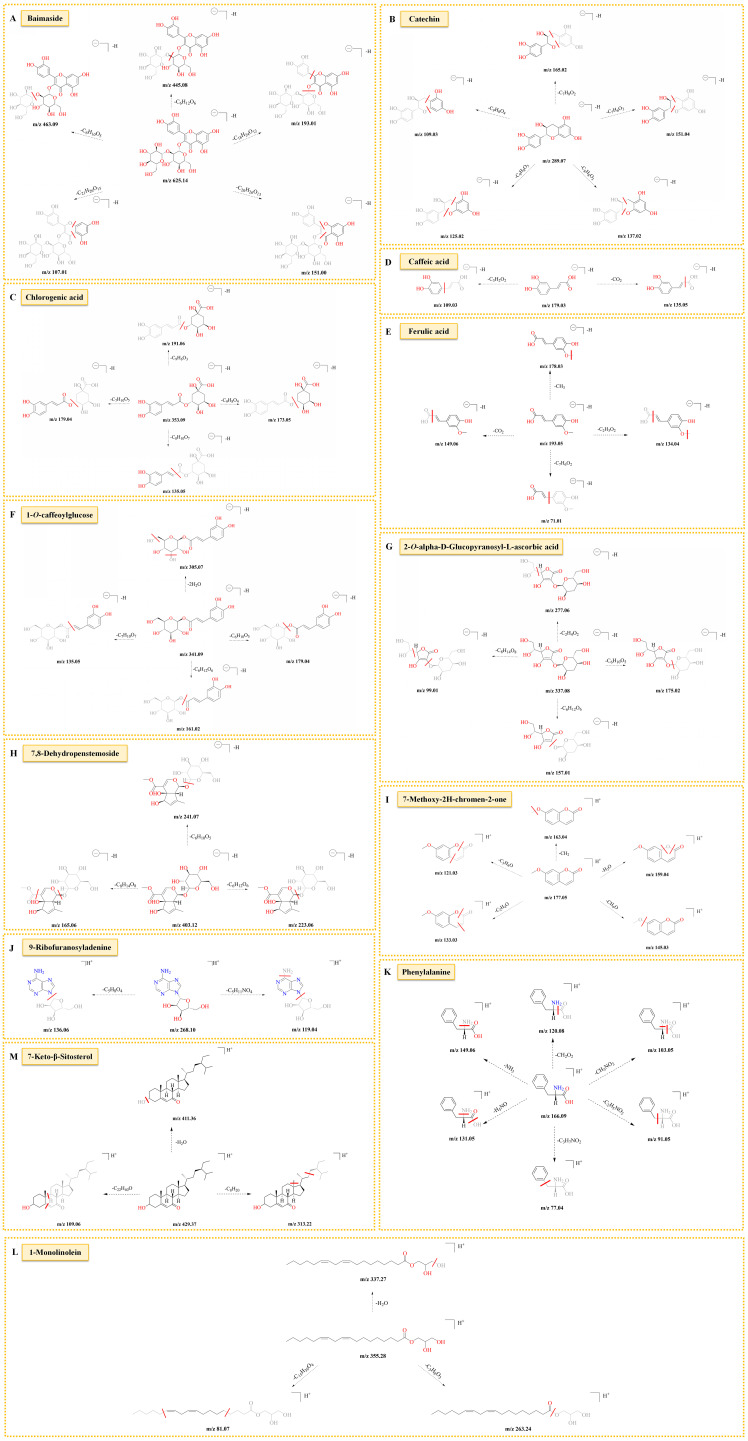
Identification process and fragmentation pathway of Baimaside (**A**), Catechin (**B**), Chlorogenic acid (**C**), Caffeic acid (**D**), Ferulic acid (**E**), 1-*O*-caffeoylglucose (**F**), 2-*O*-alpha-D-Glucopyranosyl-L-ascorbic acid (**G**), 7,8-Dehydropenstemoside (**H**), 7-Methoxy-2H-chromen-2-one (**I**), 9-Ribofuranosyladenine (**J**), Phenylalanine (**K**), 1-Monolinolein (**L**) and 7-Keto-β-Sitosterol (**M**). Red indicates oxygen-containing functional groups, and blue indicates nitrogen-containing functional groups.

Identification of flavonoids and their derivatives was mainly based on characteristic fragmentation patterns of glycosidic bonds and the Retro-Diels-Alder (RDA) cleavage of aglycones. Taking the representative compound Baimaside as an example, in the ESI^−^ mode, the deprotonated molecular ion [M−H]^−^ was observed at *m*/*z* 625.14 (C_27_H_29_O_17_^−^, mass error 0.57 ppm). MS/MS analysis of this ion revealed that it first lost a hexosyl moiety, producing the fragment ion at *m*/*z* 463.09 [M−H−C_6_H_10_O_5_]^−^, which subsequently lost one molecule of water to yield *m*/*z* 445.08 [M−H−C_6_H_10_O_5_−H_2_O]^−^. In addition, characteristic ions related to the aglycone structure and its secondary fragmentation (*m*/*z* 193.01, 151.00, 107.01) were also observed in the spectrum. Therefore, this compound was identified as Baimaside (Figure 3A).

Hyperoside was detected in positive ion mode with its quasi-molecular ion [M+H]^+^ at *m*/*z* 465.10. In its MS/MS spectrum, this ion lost a galactosyl moiety to produce the aglycone ion at *m*/*z* 303.05 [M+H−C_6_H_10_O_5_]^+^. The aglycone ion further underwent RDA cleavage, generating a characteristic fragment ion at *m*/*z* 153.02, which is consistent with the fragmentation behavior of Hyperoside. Thus, the compound was identified as Hyperoside.

In addition, for flavanols such as Catechin, the deprotonated molecular ion [M−H]^−^ was detected at *m*/*z* 289.07 in negative ion mode. MS/MS analysis revealed that this ion primarily underwent retro-aldol cleavage of the C-ring, leading to carbon skeleton fragmentation and the loss of large neutral fragments, directly generating a series of characteristic fragment ions representing the A-, B-, and C-ring structures, including *m*/*z* 165.02, *m*/*z* 151.04, *m*/*z* 137.02, and *m*/*z* 125.02, as well as a secondary fragment ion at *m*/*z* 109.03 (Figure 3B).

For flavonoid aglycones such as Quercetin, the protonated molecular ion [M+H]^+^ was observed at *m*/*z* 303.05. MS/MS analysis showed that this ion underwent retro-Diels-Alder (RDA) cleavage to produce a characteristic fragment ion at *m*/*z* 153.02, which is consistent with the mass spectral features of Quercetin.

Based on the above comprehensive analysis, a variety of flavonoids and their derivatives were successfully identified, including flavonols and their glycosides, flavones, flavanols, and dimers.

#### 2.1.2. Characterization and Identification of Organic Acids and Their Derivatives

This class of constituents mainly includes caffeoylquinic acids and their isomers, as well as simple hydroxycinnamic acids. Their mass spectrometric fragmentation behavior exhibits notable regularity: in negative ion mode, the [M−H]^−^ ions commonly undergo decarboxylation, dehydration, and cleavage of glycosidic bonds.

Caffeoylquinic acid compounds were the main organic acids identified in this study. Taking Chlorogenic acid as an example, its deprotonated molecular ion [M−H]^−^ was observed at *m*/*z* 353.09 (C_16_H_17_O_9_^−^, mass error −1.87 ppm). In the MS/MS spectrum, two characteristic fragmentation pathways were observed. In the quinic acid skeleton pathway, the ester bond underwent cleavage with the loss of a caffeoyl moiety, generating the characteristic quinic acid fragment ion at *m*/*z* 191.06 [M−H−C_9_H_6_O_3_]^−^, which further dehydrated to produce the secondary fragment ion at *m*/*z* 173.05. In the caffeic acid skeleton pathway, cleavage of the ester bond in another direction afforded the caffeic acid fragment ion at *m*/*z* 179.04 [M−H−C_7_H_10_O_5_]^−^, which subsequently decarboxylated to yield the secondary fragment ion at *m*/*z* 135.05. These specific fragment ions and their corresponding loss patterns further confirmed the structural assignment of Chlorogenic acid (Figure 3C).

Additionally, the identification of simple hydroxycinnamic acids was mainly based on their decarboxylation and side-chain cleavage. Taking caffeic acid as an example, its [M−H]^−^ ion was observed at *m*/*z* 179.03 (C_9_H_7_O_4_^−^, mass error −0.06 ppm). In its MS/MS spectrum, this ion readily underwent decarboxylation to produce the fragment ion at *m*/*z* 135.05 [M−H−CO_2_]^−^. In addition, the ion at *m*/*z* 109.03 was observed, which is presumed to arise from further cleavage of the side chain (Figure 3D).

Ferulic acid is a methoxy derivative of caffeic acid. Its [M−H]^−^ ion was observed at *m*/*z* 193.05 (C_10_H_9_O_4_^−^, mass error −1.74 ppm). MS/MS analysis revealed that its fragmentation pathway exhibited characteristic features of the methoxy group. On the one hand, the precursor ion lost a methyl radical to generate *m*/*z* 178.03 [M−H−CH_3_]^−^; on the other hand, it underwent decarboxylation to produce *m*/*z* 149.06 [M−H−CO_2_]^−^, which further lost a methyl radical to yield *m*/*z* 134.04. In addition, a low-mass ion at *m*/*z* 71.01, arising from side-chain cleavage, was observed in the spectrum, corresponding to a stable oxygen-containing fragment formed after the breakdown of the side-chain carbon skeleton. The complete fragmentation sequence—from demethylation and decarboxylation of the precursor ion to the full cleavage of the side chain—constitutes key mass spectrometric evidence for the identification of Ferulic acid (Figure 3E).

In addition, other organic acid constituents such as Benzoic acid, Citric acid, Vanillic acid and Sinapic Acid were also identified.

#### 2.1.3. Characterization and Identification of Carbohydrates and Their Derivatives

In this analysis, multiple carbohydrate components were identified, including monosaccharides, oligosaccharides, phenylethanoid glycosides, and other types. The mass spectrometric fragmentation of these components exhibits distinctive characteristics. In negative ion mode, cleavage of glycosidic bonds frequently occurs, with the loss of one or more glycosyl moieties to generate the corresponding aglycone or secondary glycoside ions, which may be accompanied by processes such as dehydration and ring-opening rearrangement.

The fragmentation of glycosidic ester components reflects the loss of both glycosyl and acyl moieties. Taking 1-*O*-caffeoylglucose as an example, its deprotonated molecular ion [M−H]^−^ was observed at *m*/*z* 341.09 (C_15_H_17_O_9_^−^, mass error −0.60 ppm). The MS/MS spectrum exhibited multiple fragmentation pathways. On one hand, cleavage of the acyl-oxygen bond occurred, with the loss of a dehydrated glucose moiety, generating the caffeate anion at *m*/*z* 179.04 [M−H−C_6_H_10_O_5_]^−^, which serves as key evidence for ester bond cleavage. The caffeate anion further lost one molecule of water to yield *m*/*z* 161.02 [M−H−C_6_H_10_O_5_−H_2_O]^−^, or underwent characteristic decarboxylation to produce *m*/*z* 135.05 [M−H−C_6_H_10_O_5_−CO_2_]^−^. On the other hand, the precursor ion lost two molecules of water to generate *m*/*z* 305.07 [M−H−2H_2_O]^−^. These fragment ions constitute important evidence for the identification of 1-*O*-caffeoylglucose (Figure 3F).

Among the glycoside components, the fragmentation behavior of 2-*O*-alpha-D-glucopyranosyl-L-ascorbic acid simultaneously reflects glycosidic bond cleavage and characteristic fragmentation of the ascorbic acid aglycone. In negative ion mode, its deprotonated molecular ion [M−H]^−^ was detected at *m*/*z* 337.08 (C_12_H_18_O_11_^−^, mass error 1.71 ppm). MS/MS analysis revealed multiple fragmentation pathways. On one hand, the precursor ion underwent glycosidic bond cleavage with the loss of one glucose residue, generating the ascorbic acid aglycone anion at *m*/*z* 175.02, which is a characteristic fragmentation of glycosides. This aglycone fragment further lost one molecule of water to produce *m*/*z* 157.01, or underwent side-chain cleavage to yield *m*/*z* 99.01. On the other hand, the precursor ion could undergo direct bond cleavage to generate the fragment at *m*/*z* 277.06. These fragmentation ions collectively constitute important evidence for the identification of 2-*O*-alpha-D-glucopyranosyl-L-ascorbic acid (Figure 3G).

During mass spectrometric fragmentation of carbohydrate components, glycosidic bond cleavage is the most common feature. Many types of glycosides preferentially lose glycosyl moieties to generate aglycone or secondary glycoside ions, and the mass of the lost glycosyl group can serve as a basis for inferring the type of sugar residue.

#### 2.1.4. Characterization and Identification of Terpenoids and Their Derivatives

Terpenoids and their derivatives are structurally diverse, and their mass spectrometric fragmentation patterns mainly include: triterpenoids readily lose carboxyl groups and water molecules, generating characteristic skeletal fragment ions; iridoids and their glycosides commonly undergo glycosidic bond cleavage, ring opening, and consecutive losses of small neutral fragments. The following is an analysis of the identification and fragmentation pathway of 7,8-dehydropenstemoside identified based on the accurate mass and MS/MS information provided by high-resolution mass spectrometry.

7,8-Dehydropenstemoside is an iridoid glycoside. Its deprotonated molecular ion [M−H]^−^ (C_17_H_24_O_11_^−^, mass error −1.22 ppm) was detected in negative ion mode. This ion underwent glycosidic bond cleavage with the loss of a dehydrated hexosyl moiety to generate the aglycone ion at *m*/*z* 241.07. Subsequently, the aglycone ion further dehydrated to produce the fragment at *m*/*z* 223.06. Alternatively, the precursor ion could also undergo side-chain cleavage or ring-opening rearrangement to yield *m*/*z* 165.06. This mass spectrometric behavior—stepwise loss of the sugar moiety followed by fragmentation of the aglycone skeleton—provides important evidence for the identification of iridoid glycosides (Figure 3H).

#### 2.1.5. Characterization and Identification of Coumarins and Their Derivatives

The mass spectrometric fragmentation of this class of constituents exhibits distinctive characteristics, commonly involving the opening of the lactone ring, the loss of small neutral molecules such as CO, and the cleavage of substituents such as methoxy and glycosyl groups. Taking 7-methoxy-2H-chromen-2-one as an example, its protonated molecular ion [M+H]^+^ was detected in positive ion mode at *m*/*z* 177.05 (C_10_H_9_O_3_^+^, mass error 0.80 ppm), along with five major characteristic fragments: *m*/*z* 163.04, 159.04, 145.03, 133.03, and 121.03. Among these, *m*/*z* 163.04 may arise from the loss of a methylene group from the precursor ion; *m*/*z* 159.04 may be formed by dehydration of the precursor ion; *m*/*z* 145.03 may originate from a rearrangement elimination involving the loss of CH_4_O, suggesting the presence of a methoxy substituent; *m*/*z* 133.03 and *m*/*z* 121.03 can be inferred as small fragment ions formed by the sequential loss of small neutral fragments after the opening of the lactone ring of the parent nucleus (Figure 3I).

#### 2.1.6. Characterization and Identification of Organonitrogen Compounds and Their Derivatives

In this analysis, nitrogen-containing compounds were identified, including nucleosides, amides, vitamins, amino acids, and sphingosine derivatives. The mass spectrometric fragmentation behaviors of this class of constituents are diverse: in positive ion mode ([M+H]^+^), nitrogen-containing compounds often undergo characteristic cleavages involving the nitrogen atom, such as the loss of ammonia (NH_3_, 17 Da) or substituents on the nitrogen-containing heterocyclic ring; nucleosides readily undergo glycosidic bond cleavage to generate characteristic base ions, while amides frequently produce characteristic fragment clusters in the low-mass region.

9-ribofuranosyladenine is a representative purine nucleoside compound. In positive ion mode, its protonated molecular ion [M+H]^+^ was detected at *m*/*z* 268.10 (C_10_H_14_N_5_O_4_^+^, mass error 0.90 ppm). Its MS/MS spectrum revealed two major fragmentation pathways: first, cleavage of the glycosidic bond with the loss of a ribose residue, generating the purine base ion at *m*/*z* 136.06, which is a key ion for the identification of adenosine and its derivatives. This adenine ion could further lose one molecule of ammonia to produce the fragment at *m*/*z* 119.04. This sequential fragmentation process of “loss of sugar—loss of ammonia” (Figure 3J) is consistent with the common fragmentation behavior of purine nucleoside compounds in positive ion mode, providing evidence for the structural confirmation of 9-ribofuranosyladenine.

The mass spectral fragmentation of phenylalanine conforms to the typical cleavage patterns of amino acids. In positive ion mode, its protonated molecular ion [M+H]^+^ was detected at *m*/*z* 166.09 (C_9_H_12_NO_2_^+^, mass error −0.92 ppm). Analysis of its MS/MS spectrum revealed that the precursor ion first lost one molecule of ammonia to generate the imine ion at *m*/*z* 149.06 [M+H−NH_3_]^+^. This is a characteristic fragmentation reaction of α-amino acids. This fragment ion could undergo further complex cleavages: *m*/*z* 149.06 could subsequently lose one molecule of water to yield *m*/*z* 131.05 [M+H−NH_3_−H_2_O]^+^. Alternatively, via a rearrangement reaction, the precursor ion directly lost formic acid to give *m*/*z* 120.08 [M+H−HCOOH]^+^, which could be assigned to the benzylmethylamine cation. These fragments further dissociated, ultimately leading to the highly characteristic benzyl ion at *m*/*z* 91.05 and even smaller fragment ions at *m*/*z* 77.04. These fragments serve as strong evidence for the identification of amino acids containing a benzene ring (Figure 3K).

#### 2.1.7. Characterization and Identification of Lipids and Their Derivatives

The lipid components identified in this study mainly include free fatty acids, oxygenated fatty acids, and simple glycerides. Fatty acids readily undergo α-cleavage, dehydration, and chain cleavage induced at carbon–carbon double bonds, generating characteristic fragment ions. These fragments are crucial for inferring the carbon chain length, positions of double bonds, and oxygen-containing functional groups.

Taking 1-Monolinolein as an example, its protonated molecular ion [M+H]^+^ was detected in positive ion mode at *m*/*z* 355.28 (C_21_H_39_O_4_^+^, mass error 0.04 ppm). MS/MS analysis revealed multiple fragmentation pathways. First, the precursor ion lost one molecule of water to generate the fragment ion at *m*/*z* 337.27 [M+H−H_2_O]^+^, which is a typical dehydration feature of glycerolipids. Subsequently, cleavage of the ester bond led to the loss of the glycerol skeleton as a neutral molecule, producing the fragment at *m*/*z* 263.24. This fragment corresponds to protonated linoleic acid and is a key ion for confirming the composition of the acyl chain. In addition, the characteristic fragment ion at *m*/*z* 81.07, arising from double-bond-induced cleavage of the linoleic acid long chain, was observed in the spectrum, further supporting the structural features of the polyunsaturated fatty acid chain (Figure 3L).

#### 2.1.8. Characterization and Identification of Steroids and Their Derivatives

The mass spectrometric fragmentation behavior of steroidal compounds exhibits distinctive characteristics, mainly involving cleavage of the steroid nucleus and loss of the side chain. 7-Keto-β-sitosterol is a major oxidized sterol identified in this study. In positive ion mode, its protonated molecular ion [M+H]^+^ was detected at *m*/*z* 429.37 (C_29_H_49_O_2_^+^, mass error 1.68 ppm). MS/MS analysis of this precursor ion showed that it could lose one molecule of water molecule to generate the fragment ion at *m*/*z* 411.36 [M+H−H_2_O]^+^, which is a common primary cleavage of oxygenated sterols. An important characteristic fragment ion was observed at *m*/*z* 313.22 [M+H−C_8_H_20_]^+^, arising from cleavage of the sterol side chain; this fragmentation provides key information regarding the side chain length and linkage mode. The ion at *m*/*z* 109.06 originates from cleavage of the steroidal nucleus. These fragments confirm the basic skeleton of this compound, which consists of a steroid nucleus with an attached side chain (Figure 3M).

#### 2.1.9. Characterization and Identification of Other Constituents

In this compositional analysis, components that could not be classified into the above major categories were also identified, mainly including aldehydes, ketones, cyclitols, and a few compounds with special structures. The mass spectrometric fragmentation behaviors of these components vary, but they still follow the characteristic fragmentation rules of organic compound functional groups, such as dehydration of alcohols, α-cleavage of ketones, and benzylic cleavage.

### 2.2. Multivariate Statistical Analysis of L. bulbifera, G. diversifolia, and U. mairei

To elucidate the overall differences in chemical composition among *L. bulbifera*, *G. diversifolia* and *U. mairei*, and to identify potential discriminatory markers, multivariate statistical analysis was performed based on the peak area datasets of chemical constituents in each sample obtained from UPLC-Q-TOF-MS/MS (Figure 1A,B).

#### 2.2.1. Principal Component Analysis

**Figure 4:**
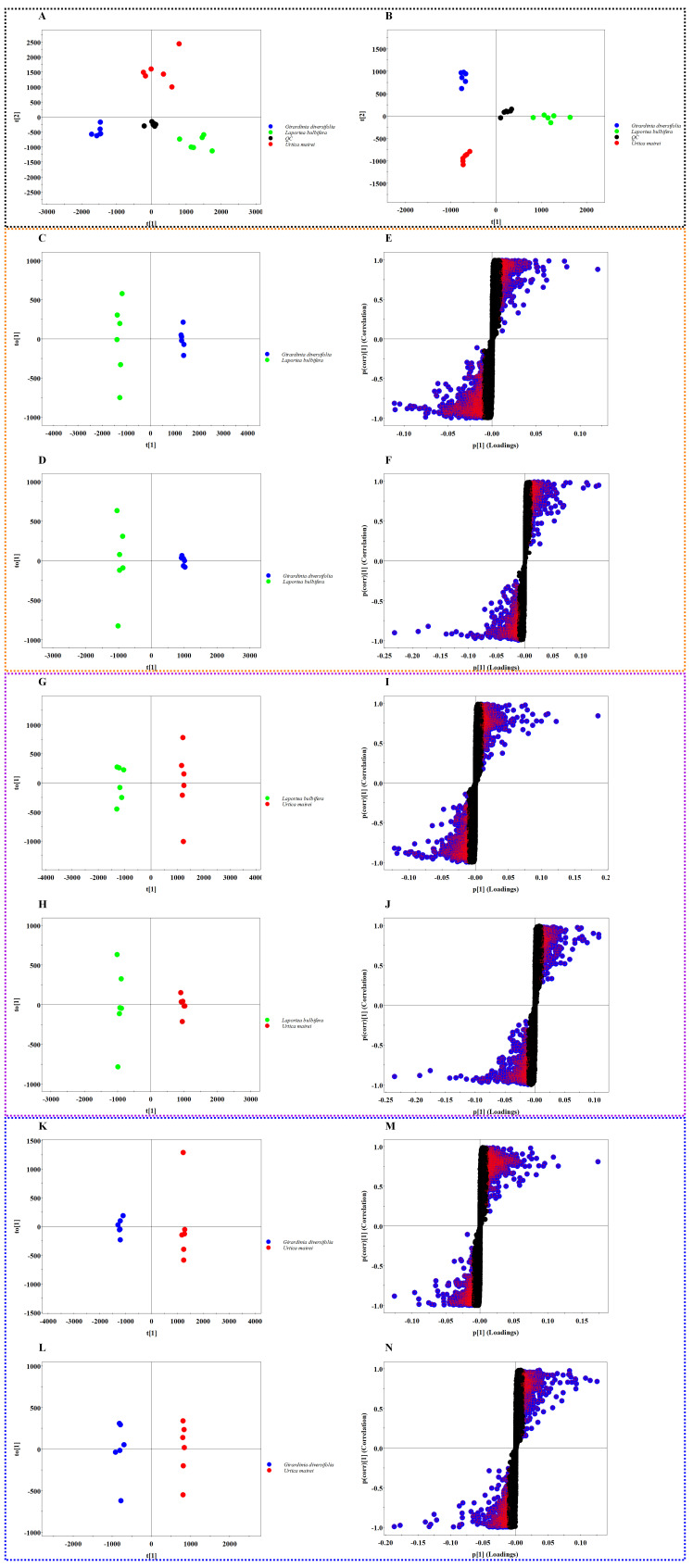
Multivariate statistical analysis based on chemical compounds from *L. bulbifera*, *G. diversifolia*, and *U. mairei*. PCA score plots of *L. bulbifera*, *G. diversifolia*, and *U. mairei* in positive ion mode (**A**) and negative ion mode (**B**). OPLS-DA score plots of *L. bulbifera* and *G. diversifolia* in positive ion mode (**C**) and negative ion mode (**D**). S-plots of *L. bulbifera* and *G. diversifolia* in positive ion mode (**E**) and negative ion mode (**F**). OPLS-DA score plots of *L. bulbifera* and *U. mairei* in positive ion mode (**G**) and negative ion mode (**H**). S-plots of *L. bulbifera* and *U. mairei* in positive ion mode (**I**) and negative ion mode (**J**). OPLS-DA score plots of *G. diversifolia* and *U. mairei* in positive ion mode (**K**) and negative ion mode (**L**). S-plots of *G. diversifolia* and *U. mairei* in positive ion mode (**M**) and negative ion mode (**N**). Points located at the upper-right and lower-left extremes of the “S” shape represent potential key differentiating variables with high contribution and strong reliability.

To observe the overall clustering trends of the samples and avoid potential bias caused by artificial grouping, an unsupervised PCA was initially performed on the raw data of all samples, including the QC samples. The results demonstrated that all QC samples clustered tightly on the score plot, verifying the instrument stability and data reliability throughout the entire analytical sequence. All test samples separated into three relatively independent clusters according to their species, without any overlap (Figure 4A,B). These findings clearly indicate that differences exist in the overall chemical profiles of the three species, which provided a basis for the subsequent pairwise comparisons among the three groups and the screening of specific discriminating components.

#### 2.2.2. Analysis of Compositional Differences Between *L. bulbifera* and *G. diversifolia*

*L. bulbifera* and *G. diversifolia* are the two botanical origins of Honghema stipulated in the *Quality Standards for Chinese Medicinal Materials and Ethnic Medicinal Materials of Guizhou Province* (2019 edition) [2]. To identify the differential components responsible for the separation of *L. bulbifera* and *G. diversifolia*, an OPLS-DA model was established. The OPLS-DA score plots demonstrated that samples from the *L. bulbifera* and *G. diversifolia* groups separated into two distinct clusters (Figure 4C–F), whereas no overlap was observed between the two groups, indicating that *L. bulbifera* and *G. diversifolia* possessed stable and significant differences in their chemical compositions.

**Figure 5:**
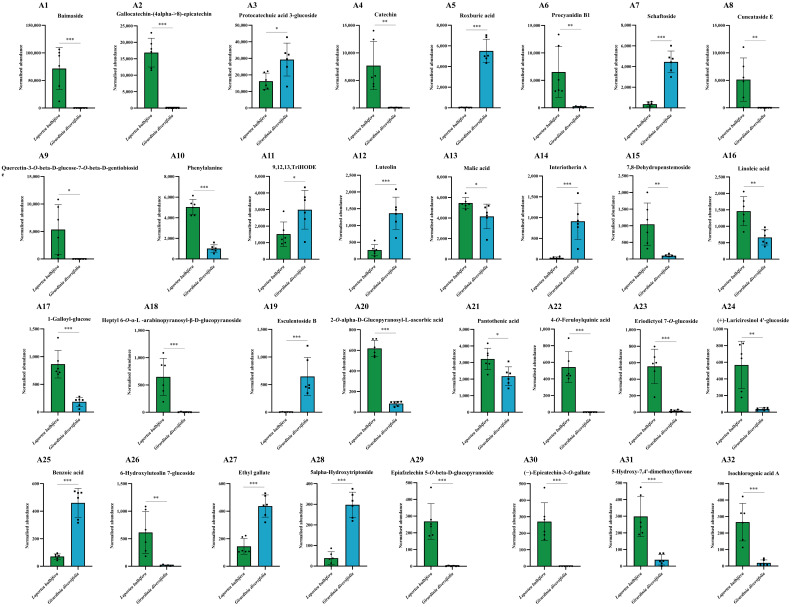
The normalised abundance of the thirty-two significantly differential components between *L. bulbifera* and *G. diversifolia* (* *p* < 0.05, ** *p* < 0.01, *** *p* < 0.001).

To screen the differential constituents contributing significantly to the discrimination between the two groups, differential ions were initially selected according to the criteria of VIP values > 1.0 and *p* < 0.05. The MS/MS fragment ions of the selected differential features were subsequently matched and verified using UNIFI 1.9.4 software. The structures of the compounds were further confirmed by comparison with reference standards and previously reported fragmentation patterns. In total, 32 significantly differential components were identified (Table 4; Figure 2G,H and Figure 5(A1–A32)). Regarding the distribution of component types, 22 differential components exhibited higher relative abundances in *L. bulbifera*, with flavonoids being the most prevalent, which is consistent with the characteristics of *L. bulbifera* as a flavonoid-rich species. Conversely, 10 differential components exhibited higher relative abundances in *G. diversifolia*, consisting primarily of organic acids and terpenoids, which is consistent with the high-abundance dominant component profile of *G. diversifolia*. These differential constituents provided a chemical basis for the discrimination between *L. bulbifera* and *G. diversifolia*.

#### 2.2.3. Analysis of Compositional Differences Between *L. bulbifera* and *U. mairei*

*U. mairei* is an easily confusable species of Honghema during commercial circulation. To establish a chemical basis for the discrimination between *L. bulbifera* and *U. mairei*, the OPLS-DA model was applied to explore the differential chemical features between *L. bulbifera* and *U. mairei*. The OPLS-DA score plots demonstrated that *L. bulbifera* and *U. mairei* could be effectively distinguished (Figure 4G–J), indicating that significant differences exist in their chemical compositions.

**Figure 6:**
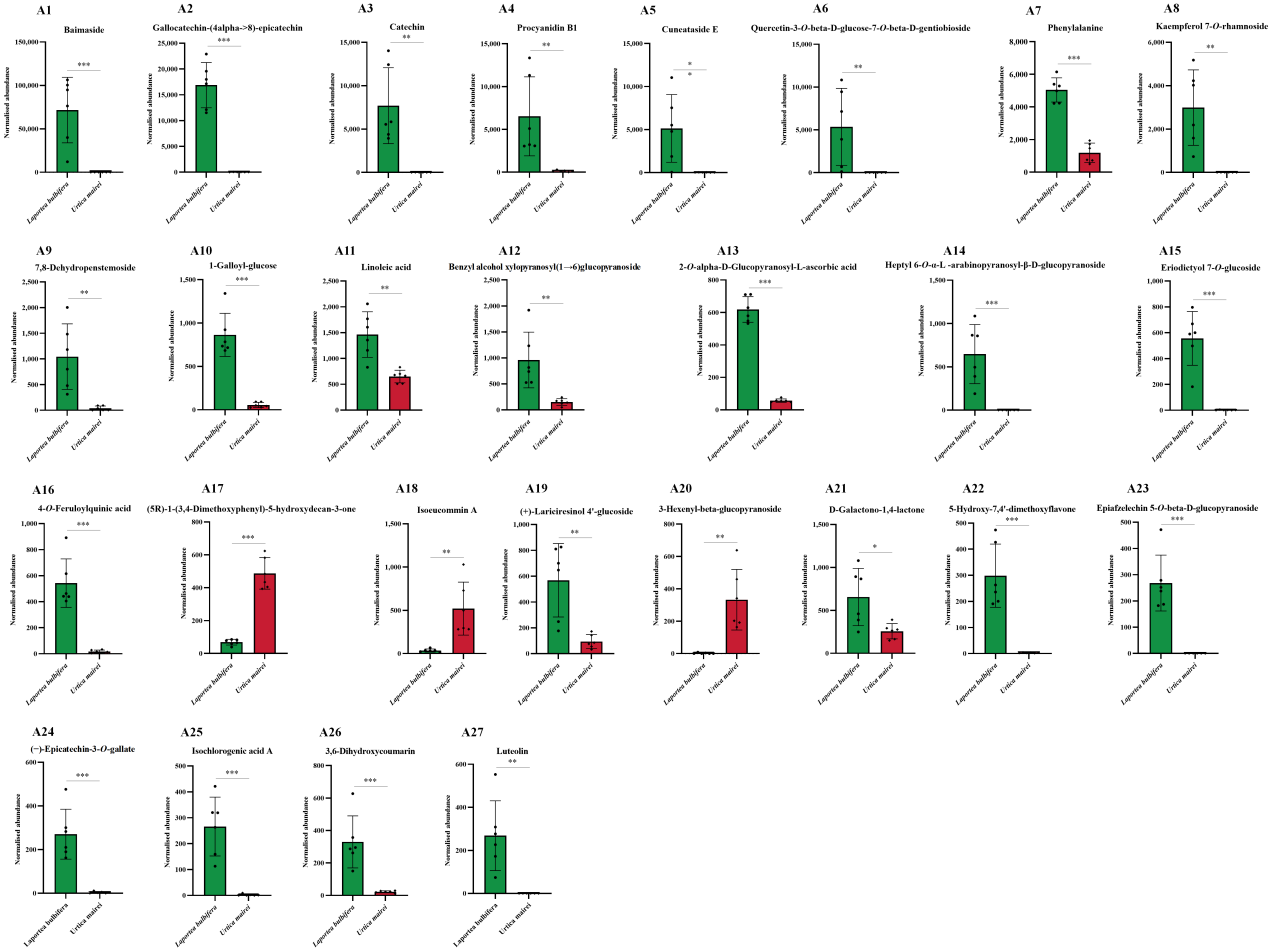
(**A1**–**A27**) The normalised abundance of the twenty-seven significantly differential components between *L. bulbifera* and *U. mairei* (* *p* < 0.05, ** *p* < 0.01, *** *p* < 0.001). Black dots represent individual sample data points (*n* = 6 per group).

To identify the differential variables that contributed significantly to the separation of the *L. bulbifera* and *U. mairei*, the VIP values generated from the OPLS-DA model were integrated with the statistical analysis of peak responses between groups using the same screening criteria of VIP > 1.0 and *p* < 0.05. A preliminary set of differential ions was thereby obtained. Following the matching of MS/MS fragments and structural confirmation, a total of 27 differential components were identified (Table 5; Figure 2I,J and Figure 6(A1–A27)). Overall, 24 differential constituents exhibited higher relative abundances in *L. bulbifera*, mainly consisting of flavonoids and carbohydrates. These constituents showed extremely low responses or were not detected in *U. mairei*. In contrast, the three constituents with higher relative abundances in *U. mairei* were mainly carbohydrates, displaying distinct differences from the predominant constituent groups of *L. bulbifera*. These differential constituents provided a chemical basis for the discrimination between *L. bulbifera* and *U. mairei*.

#### 2.2.4. Analysis of Compositional Differences Between *G. diversifolia* and *U. mairei*

The previous PCA results revealed an evident separation trend between *G. diversifolia* and *U. mairei*. To further clarify the differences in their chemical compositions, an OPLS-DA model was established. The OPLS-DA score plots showed that the samples from the *G. diversifolia* and *U. mairei* were completely separated without overlapping observations (Figure 4K–N), indicating that there were stable and identifiable differences in the chemical compositions between *G. diversifolia*, a botanical origin of Honghema, and its easily confusable species, *U. mairei*.

**Figure 7:**
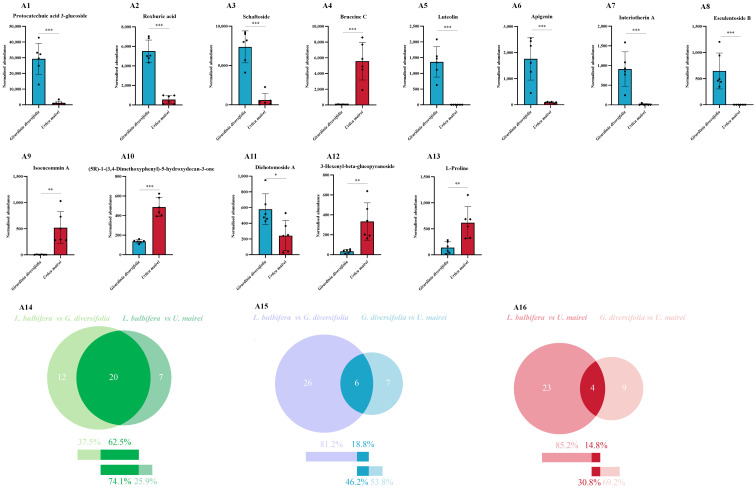
(**A1**–**A13**) The normalised abundance of the thirteen significantly differential components between *G. diversifolia* and *U. mairei* (* *p* < 0.05, ** *p* < 0.01, *** *p* < 0.001). (**A14**) The Venn diagram of significantly differential components between *L. bulbifera* vs. *G. diversifolia* and *L. bulbifera* vs. *U. mairei.* (**A15**) The Venn diagram of significantly differential components between *L. bulbifera* vs. *G. diversifolia* and *G. diversifolia* vs. *U. mairei.* (**A16**) The Venn diagram of significantly differential components between *L. bulbifera* vs. *U. mairei* and *G. diversifolia* vs. *U. mairei.*

Following the consistent screening criteria for differential ions (VIP > 1.0, *p* < 0.05), and incorporating MS/MS fragment analysis and structural identification, 13 differential components were identified between *G. diversifolia* and *U. mairei* (Table 6; Figure 2K,L and Figure 7(A1–A13)). Regarding the distribution patterns of these constituents, 8 differential constituents exhibited higher relative abundances in *G. diversifolia*, mainly comprising terpenoids and organic acids. In contrast, 5 differential constituents showed higher relative abundances in *U. mairei*, predominantly belonging to carbohydrates. These differential constituents covered the characteristic predominant constituent groups of the two plant species and provided evidence for the chemical discrimination of *G. diversifolia* from its commonly confused species *U. mairei*.

#### 2.2.5. Screening of Characteristic Constituents in the Three Species Based on Venn Diagram Analysis

To further screen highly specific and stable characteristic differential constituents that could simultaneously discriminate each species from the other two related plants, the differential constituents obtained from the three pairwise comparisons, including 32 constituents from *L. bulbifera* vs. *G. diversifolia*, 27 constituents from *L. bulbifera* vs. *U. mairei*, and 13 constituents from *G. diversifolia* vs. *U. mairei*, were subjected to Venn diagram analysis to screen characteristic constituents of the three plant species.

Twenty components, including Baimaside, were identified as common differential constituents in *L. bulbifera* compared with *G. diversifolia* and *U. mairei* (Figure 7(A14)). These components exhibited relatively high responses in *L. bulbifera* and were primarily flavonoids, suggesting them as potential characteristic markers for *L. bulbifera*. Six constituents, including Esculentoside B and Roxburic acid, were identified as common differential constituents of *G. diversifolia* compared with both *L. bulbifera* and *U. mairei* (Figure 7(A15)). These constituents showed relatively higher responses in *G. diversifolia* and were mainly represented by terpenoids, indicating their potential as candidate characteristic differential constituents of *G. diversifolia*. Furthermore, four constituents, including Isoeucommin A and 3-Hexenyl-beta-glucopyranoside, were identified as common differential constituents of *U. mairei* compared with both *L. bulbifera* and *G. diversifolia* (Figure 7(A16)). These constituents exhibited relatively higher responses in *U. mairei* and were mainly classified as carbohydrates, suggesting their potential as candidate characteristic differential constituents of *U. mairei*.

Through Venn diagram analysis, characteristic constituents of the three plant species were further screened from the pairwise differential constituents. These markers exhibit higher species specificity compared with those derived from single pairwise comparisons and provide a valuable reference for the chemical discrimination of these three plant species.

## 3. Discussion

In this study, ultra-high-performance liquid chromatography coupled with high-resolution mass spectrometry combined with multivariate statistical analysis was employed to systematically characterise the chemical compositions of the two botanical origins of Honghema and its easily confusable species *U. mairei*. The results demonstrated that the three plant species differed not only in the number of detected chemical constituents but also in their overall chemical profiles. The differential constituents screened in this study could serve as potential chemical markers for the discrimination of Honghema and its easily confusable species, providing valuable reference data for improving the quality standards of Honghema.

Regarding chemical identification, the three species exhibited markedly different compositional characteristics and significantly different predominant constituent classes. Flavonoids were the most prevalent constituents in *L. bulbifera*, accounting for 30.08%, with flavonoid glycosides such as Baimaside and Quercetin-3-*O*-beta-D-glucose-7-*O*-beta-D-gentiobioside serving as characteristic compounds. These, alongside organic acids and carbohydrates, accounted for nearly 70% of the total constituents, representing the major chemical composition of *L. bulbifera*. *G. diversifolia* showed a distinct profile where organic acids were the predominant group (24.68%), followed by a notable abundance of terpenoids and organonitrogen compounds, while the proportion of flavonoids was significantly lower than in *L. bulbifera*. *U. mairei* exhibited a unique composition dominated by organic acids (23.73%) and carbohydrates (18.64%), with relatively low abundances of terpenoids and flavonoids.

Unsupervised PCA revealed that samples from the three species clustered into completely independent groups, indicating significant differences in their secondary metabolite compositions. This approach overcomes the limitations of traditional morphology-based identification, which relies on intact plants and is highly susceptible to environmental variations and subjective experience. Instead, it allows for objective and reproducible discrimination through chemical fingerprinting combined with pattern recognition. Furthermore, the OPLS-DA model was used to quantify the contribution of each component to inter-group separation. This led to the identification of 32 differential components between *L. bulbifera* and *G. diversifolia*, 27 between *L. bulbifera* and *U. mairei*, and 13 between *G. diversifolia* and *U. mairei*. These differential constituents provided important chemical references for the discrimination of Honghema and its easily confusable species under different application scenarios. Furthermore, Venn diagram analysis identified highly specific species-exclusive markers. Twenty characteristic differential constituents, represented by flavonoids such as Baimaside and procyanidin B1, were screened as potential markers of *L. bulbifera* compared with *G. diversifolia* and *U. mairei*. Six characteristic differential constituents, including organic acids and terpenoids such as Roxburic acid and Esculentoside B, were identified as key markers distinguishing *G. diversifolia* from the other two species. Four characteristic differential constituents, mainly carbohydrates such as Isoeucommin A and 3-Hexenyl-beta-glucopyranoside, were screened as key markers for distinguishing *U. mairei* from *L. bulbifera* and *G. diversifolia*. These species-specific constituents provide a chemical basis for controlling medicinal homogeneity, distinguishing between the two botanical origins of Honghema, and identifying the easily confusable species *U. mairei* in commercial circulation.

It is noteworthy that several of the major components observed in this study have been reported in pharmacological literature to be associated with the anti-inflammatory, analgesic, and anti-RA activities of Honghema. For example, the flavonoid procyanidin B1 in *L. bulbifera* has been shown to inhibit NLRP3 inflammasome activation via the TLR4/MD-2, NF-κB and MAPK pathways [19]. Catechin has been reported to exert anti-inflammatory activity through the inhibition of NF-κB and MAPK signalling pathways, resulting in reduced expression of inflammatory mediators such as TNF-α and IL-6 [20,21]. Among the terpenoids identified in *G. diversifolia*, Roxburic acid has been reported to interact with TNF and its receptor TNF-R1, inhibit TNF-induced NF-κB activation, and consequently alleviate inflammatory responses [22]. Esculentoside B could inhibit NF-κB nuclear translocation and reduce the expression of inflammatory factors, thereby contributing to its anti-inflammatory activity [23]. These findings provide potential directions for subsequent selection of differential markers. As these species exhibit substantial chemical and pharmacological differences, establishing objective quality control standards that account for different botanical origins is a critical issue in the clinical application of multi-origin medicinal materials.

Regarding the application of identification methods, the botanical origin identification of Honghema currently relies primarily on traditional plant taxonomy, in which species discrimination is based on morphological characteristics of intact plants, such as leaf shape, stinging hair density, and inflorescence type [24]. However, these methods are susceptible to influence by environmental conditions and subjective experience. Furthermore, as Honghema often undergoes coarse processing, such as cutting, prior to its use as a medicinal material, the critical diagnostic morphological features are largely lost. This significantly increases the difficulty of species identification and hinders the development of objective standards capable of meeting the testing requirements within the medicinal material supply chain. The research strategy established in this study—integrating high-resolution mass spectrometry with the UNIFI database and multivariate statistical analysis—allows for direct detection of medicinal powders and decoction pieces, facilitating high-throughput, high-sensitivity characterisation and rapid structural elucidation. The key characteristic differential components identified can provide quantifiable reference data for the quality control of Honghema medicinal materials and their decoction pieces.

Despite the findings, this study has certain limitations, and future research may be expanded in three key directions. First, the accuracy of characteristic constituent identification should be further improved. Based on the 21 constituents currently confirmed using reference standards, the chemical constituent database of Honghema could be further refined through additional reference standard verification, isolation of individual compounds, and structural elucidation by nuclear magnetic resonance spectroscopy. Second, a quality evaluation system correlated with pharmacological activity should be constructed. Future work could establish quantitative methods for the identified core characteristic components to elucidate their distribution patterns across different botanical origins, geographical locations, and harvest seasons. By integrating this with *in vivo* and *in vitro* anti-inflammatory and analgesic assays, the activity of these components can be validated, thereby providing pharmacodynamic support for the selection of marker compounds in Honghema quality standards. Third, identification methods suitable for the supply chain should be refined. On one hand, mass spectrometry imaging could be employed to clarify the spatial distribution of characteristic components within plant tissues, providing more intuitive evidence for botanical origin identification. On the other hand, simplified and easily implemented detection methods, such as thin-layer chromatography and High Performance Liquid Chromatography (HPLC) characteristic fingerprints, could be developed. This would reduce reliance on high-end instrumentation, facilitate routine inspection and rapid on-site screening, and promote the translation of research findings into practical applications.

In conclusion, the screening strategy established in this study provided a reliable technical approach for the identification of Honghema and its easily confusable species. The characteristic differential components identified not only enrich our understanding of the material basis of Honghema but also offer scientific support for the quality control and refinement of quality standards for Honghema, thereby ensuring the safety and efficacy of its clinical application.

## 4. Materials and Methods

### 4.1. Sample Preparation

Honghema and its easily confusable samples (*L. bulbifera*, *G. diversifolia* and *U. mairei*) were collected from Guizhou Province, China. After recording the sample information, the impurities and soil were removed. The plants were then spread evenly in a well-ventilated, shaded, and light-protected area for air drying (shade-drying method) until use. The voucher specimens were deposited at the Institute of Chinese Materia Medica, China Academy of Chinese Medical Sciences. The specific information is shown in Supplement Table S1.

### 4.2. Instruments

A high-speed grinder (DFT-50A, LinDa Machinery Co., Ltd., Wenling, China); a Waters SYNAPT XS ion mobility time-of-flight high-resolution mass spectrometer (Waters Corporation, Milford, MA, USA); an ultrasonic cleaner (JP-040S, Shenzhen JieMeng Cleaning Equipment Co., Ltd., Shenzhen, China); a high-speed refrigerated benchtop centrifuge (H1850R, Hunan Xiangyi Laboratory Instrument Development Co., Ltd., Changsha, China); and an electronic balance (ME204/02, Mettler Toledo International Co., Ltd., Greifensee, Switzerland).

### 4.3. Reagents

Methanol (34860, Merck KGaA, Darmstadt, Germany); distilled water (HR-01, A.S. Watson Group, Hong Kong, China); formic acid (2244621, Thermo Fisher Scientific Inc., Waltham, MA, USA); acetonitrile (34851, Merck KGaA, Darmstadt, Germany). The purity and specifications of the reference standards were presented in Supplementary Table S2.

### 4.4. Preparation of the Test Solution

The dried whole plant samples of *L. bulbifera*, *G. diversifolia*, and *U. mairei* were cut into 2.5 cm segments and pulverized to obtain sample powder. An accurately weighed 1.0 g portion of the sample powder was mixed with 20 mL of 70% methanol, and then ultrasonicated for 30 min. After centrifugation at 12,000 r·min^−1^ for 10 min, the supernatant was filtered through a 0.22 μm microporous membrane, and the subsequent filtrate was collected as the test solution.

### 4.5. Preparation of QC Solutions

Aliquots (100 μL) of all test sample solutions were mixed in equal volumes, and the resulting mixture was divided into aliquots to prepare QC samples. The QC samples were regularly injected throughout the analytical sequence to monitor instrument stability and method reproducibility.

### 4.6. Preparation of Reference Standard Solutions

Each reference standard was accurately weighed and dissolved to prepare individual standard stock solutions, which were stored at 4 °C protected from light. Appropriate aliquots of each individual standard stock solution were taken and diluted to obtain the mixed standard solution.

### 4.7. Chromatographic Conditions

Chromatographic analyses of the test solution were performed using a Waters ACQUITY UPLC HSS T3 column (2.1 mm × 100 mm, 1.8 μm). The mobile phase consisted of 0.1% formic acid in water (A) and 0.1% formic acid in acetonitrile (B). Gradient elution was carried out as follows: 0–10 min, 5–100% B; 10–11 min, 100–5% B; 11–13 min, 5% B. The flow rate was 0.5 mL·min^−1^, the column temperature was maintained at 30 °C, and the injection volume was 2 μL.

### 4.8. Mass Spectrometer Conditions

Electrospray Ionization (ESI) was employed in mass spectrometry with Elevated Energy (MSE) mode with a scan time of 0.2 s. The capillary voltages were set at 2.00 kV in negative ion mode (ESI^−^) and 0.5 kV in positive ion mode (ESI^+^). The cone voltage was 40 V. The ion source temperature was 100 °C, and the desolvation temperature was 450 °C. Nitrogen was used as the desolvation gas at a flow rate of 900 L·h^−1^, and the cone gas (N_2_) flow rate was 50 L·h^−1^. The mass scanning range was set from *m*/*z* 50 to 1500. The low collision energy was set at 6 eV, and the high collision energy was ramped from 20 to 70 eV. During the analysis, leucine enkephalin (*m*/*z* 556.2766 [M+H]^+^, *m*/*z* 554.2620 [M–H]^−^) was used for mass axis calibration. The LC-MS data system was controlled and data were acquired and analyzed using MassLynx 4.1 software.

### 4.9. Identification of Chemical Constituents

By sorting out the research literature [3,4,5,6,7,8,9,10,11,12,13,14,15,16,17,18,19,25,26,27,28,29,30,31,32,33,34,35,36,37,38,39,40,41,42,43,44,45,46,47,48,49,50,51,52,53,54,55,56,57,58] of *L. bulbifera*, *G. diversifolia*, and *U. mairei*, the chemical composition database was constructed. The above information was imported into the UNIFI software and the analysis parameters were set. The mobile phase reagent was used as a reference to screen the ions that only existed in plant samples. The target compounds were matched by *m*/*z* and secondary fragment information, and the compounds corresponding to each molecular ion peak were determined in combination with literature reports.

### 4.10. Analysis of Differential Components

The raw LC-MS data were imported into Progenesis QI V2.1 software (Waters Corporation, USA) for noise reduction, peak alignment, and normalization. Adduct ions were set as [M+H]^+^, [M+Na]^+^, and [2M+H]^+^ in positive ion mode, and as [M−H]^−^, [M+Cl]^−^, [M+HCOO]^−^, and [2M−H]^−^ in negative ion mode. The processed data were subsequently exported to EZinfo 3.0 software for multivariate statistical analysis, including principal component analysis (PCA) and orthogonal projections to latent structures-discriminant analysis (OPLS-DA). Potential key differential constituents were screened based on the variable importance in projection (VIP) values and intergroup response differences, with the criteria of VIP > 1 and *p* < 0.05.

## 5. Conclusions

In this study, UPLC-Q-TOF-MS/MS, combined with multivariate statistical analysis, was employed to systematically compare the chemical compositions of the two botanical origins of Honghema, namely *L. bulbifera* and *G. diversifolia*, as well as its easily confusable species *U. mairei*. Significant differences were observed among the three plant species in terms of both constituent types and relative abundances. Flavonoids represented the predominant constituent class in *L. bulbifera*, whereas organic acids were the major constituent class in both *G. diversifolia* and *U. mairei*. Through OPLS-DA and Venn diagram analyses, characteristic differential constituents of *L. bulbifera*, *G. diversifolia*, and *U. mairei* were screened. These findings provide a basis for the botanical origin authentication and quality evaluation of Honghema and establish a foundation for ensuring the consistency, controllability, and reliability of its clinical application.

## Figures and Tables

**Table 1:** *Laportea bulbifera* chemical compounds identification.

NO.	Compound	Formula	Observed RT (min)	Molecular Mass	Ionization Model	Observed *m*/*z*	Mass Error (ppm)	Fragment Ions	CAS	Class
1	Histidine	C_6_H_9_N_3_O_2_	0.51	155.07	[M−H]^−^	154.06	0.11	74.02 [M−H−C_4_H_4_N_2_]^−^	71-00-1	Organonitrogen compounds and their derivatives
2	L-Asparagine	C_4_H_8_N_2_O_3_	0.53	132.05	[M−H]^−^	131.05	−3.89	114.02 [M−H−NH_3_]^−^ 113.04 [M−H−H_2_O]^−^ 72.01 [M−H−C_2_H_5_NO]^−^	70-47-3	Organonitrogen compounds and their derivatives
3	D-Raffinose	C_18_H_32_O_16_	0.55	504.17	[M+Cl]^−^	539.14	−0.67	341.11 [M−H−C_6_H_10_O_5_]^−^ 323.10 [M−H−C_6_H_12_O_6_]^−^ 179.06 [M−H−C_12_H_20_O_10_]^−^ 175.02 [M−H−C_12_H_24_O_10_]^−^ 161.05 [M−H−C_12_H_22_O_11_]^−^ 143.04 [M−H−C_12_H_24_O_12_]^−^	512-69-6	Carbohydrates and their derivatives
4	L-glutamic acid	C_5_H_9_NO_4_	0.56	147.05	[M+H]^+^	148.06	0.84	130.05 [M+H−H_2_O]^+^ 104.07 [M+H−CO_2_]^+^ 74.02 [M+H−C_3_H_6_O_2_]^+^	56-86-0	Organonitrogen compounds and their derivatives
5	Maltose	C_12_H_22_O_11_	0.56	342.12	[M+Cl]^−^	377.09	−1.50	323.10 [M−H−H_2_O]^−^ 179.06 [M−H−C_6_H_10_O_5_]^−^ 161.05 [M−H−C_6_H_12_O_6_]^−^	69-79-4	Carbohydrates and their derivatives
6	D-Galactono-1,4-lactone	C_6_H_10_O_6_	0.57	178.05	[M+COOH]^−^	223.05	−2.05	159.03 [M−H−H_2_O]^−^ 147.03 [M−H−CH_2_O]^−^ 117.02 [M−H−C_2_H_4_O_2_]^−^ 87.01 [M−H−C_3_H_6_O_3_]^−^	2782-07-2	Carbohydrates and their derivatives
7	2-*O*-alpha-D-Glucopyranosyl-L-ascorbic acid *	C_12_H_18_O_11_	0.61	338.08	[M−H]^−^	337.08	−1.71	277.06 [M−H−C_2_H_4_O_2_]^−^ 175.02 [M−H−C_6_H_10_O_5_]^−^ 157.01 [M−H−C_6_H_12_O_6_]^−^ 99.01 [M−H−C_8_H_14_O_8_]^−^	129499-78-1	Carbohydrates and their derivatives
8	L-Valine	C_5_H_11_NO_2_	0.61	117.08	[M+H]^+^	118.09	1.02	72.08 [M+H−CH_2_O_2_]^+^	72-18-4	Organonitrogen compounds and their derivatives
9	Malic acid	C_4_H_6_O_5_	0.62	134.02	[M−H]^−^	133.01	−3.11	115.00 [M−H−H_2_O]^−^ 72.99 [M−H−C_2_H_4_O_2_]^−^	6915-15-7	Organic acids and their derivatives
10	Citric acid *	C_6_H_8_O_7_	0.66	192.03	[M−H]^−^	191.02	−2.01	173.01 [M−H−H_2_O]^−^ 129.02 [M−H−CH_2_O_3_]^−^ 87.01 [M−H−C_3_H_4_O_4_]^−^	77-92-9	Organic acids and their derivatives
11	9-Ribofuranosyladenine *	C_10_H_13_N_5_O_4_	1.04	267.10	[M+H]^+^	268.10	0.90	136.06 [M+H−C_5_H_8_O_4_]^+^ 119.04 [M+H−C_5_H_11_NO_4_]^+^	58-61-7	Organonitrogen compounds and their derivatives
12	L-Tyrosine	C_9_H_11_NO_3_	1.06	181.07	[M+H]^+^	182.08	−0.57	165.05 [M+H−NH_3_]^+^ 147.04 [M+H−H_5_NO]^+^	60-18-4	Organonitrogen compounds and their derivatives
13	Guanosine	C_10_H_13_N_5_O_5_	1.15	283.09	[M−H]^−^	282.08	−0.68	150.04 [M−H−C_5_H_8_O_4_]^−^ 133.02 [M−H−C_5_H_11_NO_4_]^−^	118-00-3	Organonitrogen compounds and their derivatives
14	Homoarbutin	C_13_H_18_O_7_	1.28	286.11	[M+COOH]^−^	331.10	0.69	123.05 [M−H−C_6_H_10_O_5_]^−^	25712-94-1	Carbohydrates and their derivatives
15	p-Hydroxybenzoic acid	C_7_H_6_O_3_	1.44	138.03	[M−H]^−^	137.02	0.67	93.03 [M−H−CO_2_]^−^	99-96-7	Organic acids and their derivatives
16	1-Galloyl-glucose	C_13_H_16_O_10_	1.46	332.07	[M−H]^−^	331.07	0.81	168.01 [M−H−C_6_H_11_O_5_]^−^ 153.02 [M−H−C_6_H_10_O_6_]^−^	554-37-0	Carbohydrates and their derivatives
17	Cardiospermin	C_11_H_17_NO_7_	1.47	275.10	[M−H]^−^	274.09	−1.14	256.08 [M−H−H_2_O]^−^ 89.02 [M−H−C_8_H_11_NO_4_]^−^	54525-10-9	Carbohydrates and their derivatives
18	Phenylalanine *	C_9_H_11_NO_2_	1.52	165.08	[M+H]^+^	166.09	−0.92	149.06 [M+H−NH_3_]^+^ 131.05 [M+H−H_5_NO]^+^ 120.08 [M+H−CH_2_O_2_]^+^ 103.05 [M+H−CH_5_NO_2_]^+^ 91.05 [M+H−C_2_H_5_NO_2_]^+^ 77.04 [M+H−C_3_H_7_NO_2_]^+^	63-91-2	Organonitrogen compounds and their derivatives
19	Tryptophan *	C_11_H_12_N_2_O_2_	1.55	204.09	[M+H]^+^	205.10	−1.18	188.07 [M+H−NH_3_]^+^ 118.07 [M+H−C_3_H_5_NO_2_]^+^	73-22-3	Organonitrogen compounds and their derivatives
20	Pantothenic acid	C_9_H_17_NO_5_	1.61	219.11	[M+H]^+^	220.12	0.23	202.11 [M+H−H_2_O]^+^ 148.06 [M+H−C_4_H_8_O]^+^	79-83-4	Organonitrogen compounds and their derivatives
21	3,3′,5-Trihydroxy-4′,7-dimethoxyflavanone	C_17_H_16_O_7_	1.65	332.09	[M+H]^+^	333.10	2.84	165.05 [M+H−C_8_H_8_O_4_]^+^ 137.02 [M+H−C_10_H_12_O_4_]^+^	79995-67-8	Flavonoids and their derivatives
22	Protocatechuic acid 3-glucoside *	C_13_H_16_O_9_	1.66	316.08	[M−H]^−^	315.07	−0.44	152.01 [M−H−C_6_H_11_O_5_]^−^ 109.03 [M−H−C_7_H_10_O_7_]^−^ 108.02 [M−H−C_7_H_11_O_7_]^−^	20300-54-3	Carbohydrates and their derivatives
23	(-)-Epigallocatechin	C_15_H_14_O_7_	1.76	306.07	[M−H]^−^	305.07	−0.51	179.04 [M−H−C_6_H_6_O_3_]^−^ 125.02 [M−H−C_9_H_8_O_4_]^−^	970-74-1	Flavonoids and their derivatives
24	3,6-Dihydroxycoumarin	C_9_H_6_O_4_	1.82	178.03	[M+H]^+^	179.03	1.61	123.04 [M+H−2CO]+	3450-80-4	Coumarins and their derivatives
25	Gallocatechin-(4alpha->8)-epicatechin	C_30_H_26_O_13_	1.83	594.14	[M−H]^−^	593.13	0.72	441.08 [M−H−C_8_H_8_O_3_]^−^ 423.07 [M−H−C_8_H_10_O_4_]^−^ 407.08 [M−H−C_8_H_10_O_5_]^−^ 182.02 [M−H−C_22_H_19_O_8_]^−^ 125.02 [M−H−C_24_H_20_O_10_]^−^ 109.03 [M−H−C_24_H_20_O_11_]^−^	79199-56-7	Flavonoids and their derivatives
26	(−)-Epicatechin-3-*O*-gallate	C_22_H_18_O_10_	1.84	442.09	[M−H]^−^	441.08	0.69	423.07 [M−H−H_2_O]^−^ 305.07 [M−H−C_7_H_4_O_3_]^−^ 289.07 [M−H−C_7_H_4_O_4_]^−^ 125.02 [M−H−C_16_H_12_O_7_]^−^	1257-08-5	Flavonoids and their derivatives
27	Vanillic acid beta-D-glucopyranosyl ester	C_14_H_18_O_9_	1.86	330.10	[M−H]^−^	329.09	−0.76	191.06 [M−H−C_7_H_6_O_3_]^−^ 167.04 [M−H−C_6_H_10_O_5_]^−^ 165.02 [M−H−C_6_H_12_O_5_]^−^ 109.03 [M−H−C_8_H_12_O_7_]^−^	68985-14-8	Carbohydrates and their derivatives
28	Hyperoside *	C_21_H_20_O_12_	1.92	464.10	[M+H]^+^	465.10	2.52	303.05 [M+H−C_6_H_10_O_5_]^+^ 285.04 [M+H−C_6_H_12_O_6_]^+^ 247.06 [M+H−C_8_H_10_O_7_]^+^ 153.02 [M+H−C_14_H_16_O_8_]^+^	482-36-0	Flavonoids and their derivatives
29	Quercetin-3-gentiobioside-7-glucoside	C_33_H_40_O_22_	1.92	788.20	[M−H]^−^	787.19	0.39	625.14 [M−H−C_6_H_10_O_5_]^−^ 462.08 [M−H−C_12_H_21_O_10_]^−^ 445.08 [M−H−C_12_H_22_O_11_]^−^ 299.02 [M−H−C_18_H_32_O_15_]^−^	-	Flavonoids and their derivatives
30	Cryptochlorogenic acid *	C_16_H_18_O_9_	1.94	354.10	[M−H]^−^	353.09	−1.10	191.06 [M−H−C_9_H_6_O_3_]^−^ 179.04 [M−H−C_7_H_10_O_5_]^−^ 135.05 [M−H−C_8_H_10_O_7_]^−^	905-99-7	Organic acids and their derivatives
31	1H-indole-2-carbaldehyde	C_9_H_7_NO	2.06	145.05	[M+H]^+^	146.06	−0.86	118.07 [M+H−CO]^+^	19005-93-7	Organonitrogen compounds and their derivatives
32	Esculin	C_15_H_16_O_9_	2.09	340.08	[M−H]^−^	339.07	−1.46	303.05 [M−H−2H_2_O]^−^ 177.02 [M−H−C_6_H_10_O_5_]^−^ 161.02 [M−H−C_6_H_10_O_6_]^−^	531-75-9	Coumarins and their derivatives
33	Procyanidin	C_30_H_26_O_13_	2.16	594.14	[M+H]^+^	595.15	1.33	307.08 [M+H−C_15_H_12_O_6_]^+^ 291.09 [M+H−C_15_H_12_O_7_]^+^ 181.05 [M+H−C_21_H_18_O_9_]^+^	4852-22-6	Flavonoids and their derivatives
34	Vanillic acid	C_8_H_8_O_4_	2.17	168.04	[M+H]^+^	169.05	1.09	151.04 [M+H−H_2_O]^+^ 139.04 [M+H−CH_2_O]^+^ 121.03 [M+H−CH_4_O_2_]^+^ 107.05 [M+H−CH_2_O_3_]^+^	121-34-6	Organic acids and their derivatives
35	Caffeic acid	C_9_H_8_O_4_	2.17	180.04	[M−H]^−^	179.03	−0.06	135.05 [M−H−CO_2_]^−^ 109.03 [M−H−C_3_H_2_O_2_]^−^	331-39-5	Organic acids and their derivatives
36	7-Methoxy-2H-chromen-2-one *	C_10_H_8_O_3_	2.18	176.05	[M+H]^+^	177.05	0.80	163.04 [M+H−CH_2_]^+^ 159.04 [M+H−H_2_O]^+^ 145.03 [M+H−CH_4_O]^+^ 133.03 [M+H−C_2_H_4_O]^+^ 121.03 [M+H−C_3_H_4_O]^+^	531-59-9	Coumarins and their derivatives
37	Epiafzelechin 5-*O*-beta-D-glucopyranoside	C_21_H_24_O_10_	2.27	436.14	[M+COOH]^−^	481.14	0.11	301.07 [M−H−C_5_H_10_O_4_]^−^ 165.06 [M−H−C_12_H_14_O_7_]^−^ 151.04 [M−H−C_13_H_16_O_7_]^−^ 137.06 [M−H−C_13_H_14_O_8_]^−^	-	Flavonoids and their derivatives
38	Ruberythric acid	C_25_H_26_O_13_	2.29	534.14	[M−H]^−^	533.13	0.50	515.12 [M−H−H_2_O]^−^ 383.08 [M−H−C_5_H_10_O_5_]^−^	152-84-1	Organic acids and their derivatives
39	Corchoionoside A	C_19_H_32_O_8_	2.29	388.21	[M+Na]^+^	411.20	0.83	191.14 [M+H−C_6_H_14_O_7_]^+^	189351-14-2	Carbohydrates and their derivatives
40	Polygalatenoside B	C_19_H_28_O_10_	2.33	416.17	[M+Na]^+^	439.16	2.71	399.17 [M+H−H_2_O]^+^ 237.11 [M+H−C_6_H_12_O_6_]^+^	-	Carbohydrates and their derivatives
41	3-Caffeoylshikimic acid	C_16_H_16_O_8_	2.35	336.08	[M+H]^+^	337.09	2.23	291.09 [M+H−CH_2_O_2_]^+^ 215.06 [M+H−C_7_H_6_O_2_]^+^ 165.05 [M+H−C_7_H_8_O_5_]^+^ 163.04 [M+H−C_7_H_10_O_5_]^+^ 145.03 [M+H−C_7_H_12_O_6_]^+^ 135.04 [M+H−C_8_H_10_O_6_]^+^	180981-12-8	Organic acids and their derivatives
42	p-Coumaric acid	C_9_H_8_O_3_	2.37	164.05	[M−H]^−^	163.04	−0.27	119.05 [M−H−CO_2_]^−^ 93.03 [M−H−C_3_H_2_O_2_]^−^ 71.01 [M−H−C_6_H_4_O]^−^	7400-08-0	Organic acids and their derivatives
43	Protocatechualdehyde	C_7_H_6_O_3_	2.37	138.03	[M+H]^+^	139.04	2.23	111.04 [M+H−CO]^+^ 93.03 [M+H−CH_2_O_2_]^+^	139-85-5	Organic acids and their derivatives
44	Leiocarposide	C_27_H_34_O_16_	2.38	614.18	[M+Cl]^−^	649.16	2.05	289.07 [M−H−C_12_H_20_O_10_]^−^ 165.02 [M−H−C_19_H_28_O_12_]^−^ 121.03 [M−H−C_20_H_28_O_14_]^−^	71953-77-0	Carbohydrates and their derivatives
45	Isochlorogenic acid C	C_25_H_24_O_12_	2.39	516.13	[M+H]^+^	517.14	2.62	499.12 [M+H−H_2_O]^+^ 473.11 [M+H−C_2_H_4_O]^+^ 137.06 [M+H−C_17_H_16_O_10_]^+^ 111.04 [M+H−C_19_H_18_O_10_]^+^	89886-30-6	Organic acids and their derivatives
46	Secoisolariciresinol diglucoside	C_32_H_46_O_16_	2.42	686.28	[M+COOH]^−^	731.27	−3.57	361.17 [M−H−C_12_H_20_O_10_]^−^ 297.11 [M−H−C_14_H_28_O_12_]^−^ 135.05 [M−H−C_24_H_38_O_14_]^−^	158932-33-3	Others
47	Fraxin	C_16_H_18_O_10_	2.45	370.09	[M−H]^−^	369.08	0.02	205.01 [M−H−C_6_H_12_O_5_]^−^	524-30-1	Coumarins and their derivatives
48	Genipin	C_11_H_14_O_5_	2.46	226.08	[M−H]^−^	225.08	−1.41	123.05 [M−H−C_4_H_6_O_3_]^−^	6902-77-8	Terpenoids and their derivatives
49	Procyanidin B1 *	C_30_H_26_O_12_	2.49	578.14	[M−H]^−^	577.14	−0.01	451.10 [M−H−C_6_H_6_O_3_]^−^ 425.09 [M−H−C_8_H_8_O_3_]^−^ 407.08 [M−H−C_8_H_10_O_4_]^−^ 289.07 [M−H−C_15_H_12_O_6_]^−^ 245.05 [M−H−C_17_H_16_O_7_]^−^	20315-25-7	Flavonoids and their derivatives
50	Olivil 4′-*O*-beta-D-glucopyranoside	C_26_H_34_O_12_	2.50	538.21	[M−H]^−^	537.20	−0.17	339.12 [M−H−C_6_H_14_O_7_]^−^ 325.11 [M−H−C_7_H_16_O_7_]^−^ 205.05 [M−H−C_15_H_24_O_8_]^−^ 149.02 [M−H−C_18_H_28_O_9_]^−^ 135.05 [M−H−C_18_H_26_O_10_]^−^ 123.05 [M−H−C_19_H_26_O_10_]^−^ 119.05 [M−H−C_18_H_26_O_11_]^−^	76880-93-8	Carbohydrates and their derivatives
51	Quercetin-3-*O*-beta-D-glucose-7-*O*-beta-D-gentiobioside *	C_33_H_40_O_22_	2.53	788.20	[M+H]^+^	789.21	1.41	627.16 [M+H−C_6_H_10_O_5_] + 465.10 [M+H−C_12_H_20_O_10_]^+^ 303.05 [M+H−C_18_H_30_O_15_]^+^ 285.04 [M+H−C_18_H_32_O_16_]^+^	60778-02-1	Flavonoids and their derivatives
52	Ferulic acid *	C_10_H_10_O_4_	2.55	194.06	[M−H]^−^	193.05	−1.74	178.03 [M−H−CH_3_]^−^ 149.06 [M−H−CO_2_]^−^ 134.04 [M−H−C_2_H_3_O_2_]^−^ 71.01 [M−H−C_7_H_6_O_2_]^−^	1135-24-6	Organic acids and their derivatives
53	Benzyl alcohol xylopyranosyl(1 → 6)glucopyranoside	C_18_H_26_O_10_	2.56	402.15	[M−H]^−^	401.14	−1.71	269.10 [M−H−C_5_H_8_O_4_]^−^ 161.05 [M−H−C_12_H_16_O_5_]^−^	130622-31-0	Carbohydrates and their derivatives
54	Nicotiflorin	C_27_H_30_O_15_	2.63	594.16	[M+H]^+^	595.17	0.26	559.14 [M+H−2H_2_O]^+^ 177.02 [M+H−C_18_H_26_O_11_]^+^	17650-84-9	Flavonoids and their derivatives
55	3-(3-hydroxy-1-butenyl)-2,4,4-trimethyl-2-cyclohexen-1-ketone	C_13_H_20_O_2_	2.64	208.15	[M+H]^+^	209.15	−1.28	191.14 [M+H−H_2_O]^+^ 167.14 [M+H−C_2_H_2_O]^+^ 151.11 [M+H−C_3_H_6_O]^+^	-	Others
56	Creoside IV	C_17_H_32_O_10_	2.65	396.20	[M−H]^−^	395.19	−0.77	235.12 [M−H−C_7_H_12_O_4_]^−^ 191.06 [M−H−C_10_H_20_O_4_]^−^ 173.05 [M−H−C_10_H_22_O_5_]^−^ 87.01 [M−H−C_14_H_28_O_7_]^−^	-	Carbohydrates and their derivatives
57	Chlorogenic acid *	C_16_H_18_O_9_	2.66	354.10	[M−H]^−^	353.09	−1.87	191.06 [M−H−C_9_H_6_O_3_]^−^ 179.04 [M−H−C_7_H_10_O_5_]^−^ 173.05 [M−H−C_9_H_8_O_4_]^−^ 135.05 [M−H−C_8_H_10_O_7_]^−^	327-97-9	Organic acids and their derivatives
58	3′-Hydroxymelanettin	C_16_H_12_O_6_	2.68	300.06	[M+H]^+^	301.07	1.74	269.04 [M+H−CH_4_O]^+^ 175.04 [M+H−C_6_H_6_O_3_]^+^ 134.04 [M+H−C_8_H_7_O_4_]^+^	200391-95-3	Flavonoids and their derivatives
59	Sinapic Acid *	C_11_H_12_O_5_	2.69	224.07	[M−H]^−^	223.06	−3.37	179.07 [M−H−CO_2_]^−^ 164.05 [M−H−C_2_H_3_O_2_]^−^	7362-37-0	Organic acids and their derivatives
60	Isorhamnetin 3-*O*-alpha-rhamnopyranosyl-(1-2)-beta-galactopyranoside	C_28_H_32_O_16_	2.72	624.17	[M+H]^+^	625.18	−0.43	433.11 [M+H−C_7_H_12_O_6_]^+^ 123.04 [M+H−C_21_H_26_O_14_]^+^	107740-46-5	Flavonoids and their derivatives
61	Catechin *	C_15_H_14_O_6_	2.73	290.08	[M−H]^−^	289.07	−2.07	165.02 [M−H−C_7_H_8_O_2_]^−^ 151.04 [M−H−C_7_H_6_O_3_]^−^ 137.02 [M−H−C_8_H_8_O_3_]^−^ 125.02 [M−H−C_9_H_8_O_3_]^−^ 109.03 [M−H−C_9_H_8_O_4_]^−^	154-23-4	Flavonoids and their derivatives
62	Eriodictyol 7-*O*-glucoside	C_21_H_22_O_11_	2.75	450.12	[M−H]^−^	449.11	−1.27	431.10 [M−H−H_2_O]^−^ 287.06 [M−H−C_6_H_10_O_5_]^−^ 151.04 [M−H−C_13_H_14_O_8_]^−^	38965-51-4	Flavonoids and their derivatives
63	7,8-Dehydropenstemoside	C_17_H_24_O_11_	2.78	404.13	[M−H]^−^	403.12	−1.22	241.07 [M−H−C_6_H_10_O_5_]^−^ 223.06 [M−H−C_6_H_12_O_6_]^−^ 165.06 [M−H−C_8_H_14_O_8_]^−^	-	Terpenoids and their derivatives
64	5-Hydroxy-7,4′-dimethoxyflavone	C_17_H_14_O_5_	2.79	298.08	[M+COOH]^−^	343.08	−1.38	175.04 [M−H−C_7_H_6_O_2_]^−^ 167.04 [M−H−C_9_H_6_O]^−^	5128-44-9	Flavonoids and their derivatives
65	Dichotomoside A	C_26_H_32_O_13_	2.85	552.18	[M−H]^−^	551.18	−1.08	389.12 [M−H−C_6_H_10_O_5_]^−^ 193.05 [M−H−C_16_H_22_O_9_]^−^ 135.05 [M−H−C_18_H_24_O_11_]^−^	845673-17-8	Carbohydrates and their derivatives
66	1-*O*-caffeoylglucose *	C_15_H_18_O_9_	2.86	342.10	[M−H]^−^	341.09	−0.60	305.07 [M−H−2H_2_O]^−^ 179.04 [M−H−C_6_H_10_O_5_]^−^ 161.02 [M−H−C_6_H_12_O_6_]^−^ 135.05 [M−H−C_7_H_10_O_7_]^−^	14364-08-0	Carbohydrates and their derivatives
67	Robinetin	C_15_H_10_O_7_	2.90	302.04	[M+H]^+^	303.05	0.30	285.04 [M+H−H_2_O]^+^ 247.06 [M+H−2CO]^+^ 137.02 [M+H−C_8_H_6_O_4_]^+^	490-31-3	Flavonoids and their derivatives
68	Myricetin-3-*O*-α-L-rhamnopyranoside	C_21_H_20_O_12_	2.91	464.10	[M+H]^+^	465.10	2.18	447.09 [M+H−H_2_O]^+^ 303.05 [M+H−C_6_H_10_O_5_]^+^ 285.04 [M+H−C_6_H_12_O_6_]^+^ 153.02 [M+H−C_14_H_16_O_8_]^+^ 127.04 [M+H−C_15_H_14_O_9_]^+^	17912-87-7	Flavonoids and their derivatives
69	Baimaside *	C_27_H_30_O_17_	2.91	626.15	[M−H]^−^	625.14	0.57	463.09 [M−H−C_6_H_10_O_5_]^−^ 445.08 [M−H−C_6_H_12_O_6_]^−^ 193.01 [M−H−C_18_H_24_O_12_]^−^ 151.00 [M−H−C_20_H_26_O_13_]^−^ 107.01 [M−H−C_21_H_26_O_15_]^−^	18609-17-1	Flavonoids and their derivatives
70	Schaftoside	C_26_H_28_O_14_	2.92	564.15	[M+H]^+^	565.16	1.30	547.14 [M+H−H_2_O]^+^ 529.13 [M+H−2H_2_O]^+^	51938-32-0	Flavonoids and their derivatives
71	Agnuside	C_22_H_26_O_11_	2.94	466.15	[M−H]^−^	465.14	−0.80	303.09 [M−H−C_6_H_10_O_5_]^−^ 285.08 [M−H−C_6_H_12_O_6_]^−^	11027-63-7	Terpenoids and their derivatives
72	Narirutin	C_27_H_32_O_14_	2.95	580.18	[M−H]^−^	579.17	−3.43	473.11 [M−H−C_4_H_10_O_3_]^−^ 373.09 [M−H−C_8_H_14_O_6_]^−^ 311.06 [M−H−C_10_H_20_O_8_]^−^ 165.06 [M−H−C_18_H_22_O_11_]^−^	14259-46-2	Flavonoids and their derivatives
73	Pratensein-7-*O*-β-D-glucopyranoside	C_22_H_22_O_11_	3.00	462.12	[M−H]^−^	461.11	−0.46	299.06 [M−H−C_6_H_10_O_5_]^−^ 283.02 [M−H−C_7_H_14_O_5_]^−^ 267.03 [M−H−C_7_H_14_O_6_]^−^ 161.02 [M−H−C_13_H_16_O_8_]^−^ 123.05 [M−H−C_15_H_14_O_9_]^−^	36191-03-4	Flavonoids and their derivatives
74	6-Hydroxyluteolin 7-glucoside	C_21_H_20_O_12_	3.01	464.10	[M−H]^−^	463.09	0.04	283.02 [M−H−C_6_H_12_O_6_]^−^ 133.03 [M−H−C_13_H_14_O_10_]^−^	54300-65-1	Flavonoids and their derivatives
75	Tangeretin	C_20_H_20_O_7_	3.05	372.12	[M−H]^−^	371.11	−3.25	340.10 [M−H−CH_3_O]^−^ 175.04 [M−H−C_10_H_12_O_4_]^−^	481-53-8	Flavonoids and their derivatives
76	1-*O*-(4-Coumaroyl)-beta-D-glucose	C_15_H_18_O_8_	3.08	326.10	[M−H]^−^	325.09	−0.16	147.05 [M−H−C_6_H_10_O_6_]^−^ 119.06 [M−H−C_7_H_10_O_7_]^−^	7139-64-2	Carbohydrates and their derivatives
77	Isochlorogenic acid A	C_25_H_24_O_12_	3.09	516.13	[M−H]^−^	515.12	−0.47	191.06 [M−H−C_18_H_12_O_6_]^−^ 179.04 [M−H−C_16_H_16_O_8_]^−^	89919-62-0	Organic acids and their derivatives
78	(+)-Lariciresinol 4′-glucoside	C_26_H_34_O_11_	3.12	522.21	[M−H]^−^	521.20	−1.21	359.15 [M−H−C_6_H_10_O_5_]^−^ 223.10 [M−H−C_14_H_18_O_7_]^−^ 161.05 [M−H−C_20_H_24_O_6_]^−^ 135.05 [M−H−C_18_H_26_O_9_]^−^	143663-00-7	Carbohydrates and their derivatives
79	Kaempferol 3-gentiobioside	C_27_H_30_O_16_	3.14	610.15	[M+H]^+^	611.16	0.63	449.11 [M+H−C_6_H_10_O_5_]^+^ 287.06 [M+H−C_12_H_20_O_10_]^+^ 269.04 [M+H−C_12_H_22_O_11_]^+^ 153.02 [M+H−C_20_H_26_O_12_]^+^	22149-35-5	Flavonoids and their derivatives
80	4-*O*-Feruloylquinic acid	C_17_H_20_O_9_	3.19	368.11	[M−H]^−^	367.10	−0.94	191.06 [M−H−C_10_H_8_O_3_]^−^ 134.04 [M−H−C_9_H_13_O_7_]^−^	2613-86-7	Organic acids and their derivatives
81	Homoplantaginin	C_22_H_22_O_11_	3.24	462.12	[M+H]^+^	463.12	2.40	301.07 [M+H−C_6_H_10_O_5_]^+^ 286.05 [M+H−C_7_H_13_O_5_]^+^ 163.04 [M+H−C_13_H_16_O_8_]^+^	17680-84-1	Flavonoids and their derivatives
82	Isorhamnetin	C_16_H_12_O_7_	3.26	316.06	[M+H]^+^	317.07	2.50	153.02 [M+H−C_9_H_8_O_3_]^+^	480-19-3	Flavonoids and their derivatives
83	Plantagoside	C_21_H_22_O_12_	3.27	466.11	[M−H]^−^	465.10	0.23	303.05 [M−H−C_6_H_10_O_5_]^−^ 285.04 [M−H−C_6_H_12_O_6_]^−^ 161.05 [M−H−C_15_H_12_O_7_]^−^ 151.00 [M−H−C_14_H_18_O_8_]^−^ 125.02 [M−H−C_15_H_16_O_9_]^−^	78708-33-5	Flavonoids and their derivatives
84	Nystose	C_24_H_42_O_21_	3.28	666.22	[M+COOH]^−^	711.21	−9.75	339.09 [M−H−C_12_H_22_O_10_]^−^ 311.10 [M−H−C_13_H_22_O_11_]^−^ 161.05 [M−H−C_18_H_32_O_16_]^−^	13133-07-8	Carbohydrates and their derivatives
85	Rutin *	C_27_H_30_O_16_	3.30	610.15	[M+H]^+^	611.16	1.65	303.05 [M+H−C_12_H_20_O_9_]^+^ 287.06 [M+H−C_12_H_20_O_10_]^+^	153-18-4	Flavonoids and their derivatives
86	Alatoside A	C_21_H_36_O_8_	3.41	416.24	[M+COOH]^−^	461.24	−4.91	209.12 [M−H−C_9_H_18_O_5_]^−^	-	Terpenoids and their derivatives
87	Kaempferol *	C_15_H_10_O_6_	3.44	286.05	[M+H]^+^	287.06	1.37	269.04 [M+H−H_2_O]^+^ 229.05 [M+H−C_2_H_2_O_2_]^+^ 161.02 [M+H−C_6_H_6_O_3_]^+^ 153.02 [M+H−C_8_H_6_O_2_]^+^	520-18-3	Flavonoids and their derivatives
88	Kaempferol-3-*O*-β-D-glucopyranoside	C_21_H_20_O_11_	3.44	448.10	[M−H]^−^	447.09	−0.94	285.04 [M−H−C_6_H_10_O_5_]^−^ 283.02 [M−H−C_6_H_12_O_5_]^−^ 267.03 [M−H−C_6_H_12_O_6_]^−^ 151.00 [M−H−C_14_H_16_O_7_]^−^ 107.01 [M−H−C_15_H_16_O_9_]^−^	480-10-4	Flavonoids and their derivatives
89	Quercetin	C_15_H_10_O_7_	3.45	302.04	[M+H]^+^	303.05	2.21	153.02 [M+H−C_8_H_6_O_3_]^+^	117-39-5	Flavonoids and their derivatives
90	(3S,7E,9S)-9-Hydroxy-4,7-megastigmadien-3-one 9-glucoside	C_19_H_30_O_7_	3.48	370.20	[M+H]^+^	371.21	2.30	209.15 [M+H−C_6_H_10_O_5_]^+^ 191.14 [M+H−C_6_H_12_O_6_]^+^ 137.10 [M+H−C_10_H_18_O_6_]^+^	159813-37-3	Others
91	6,6′,7,7′-Tetramethoxyl-8,8’-biscoumarin	C_22_H_18_O_8_	3.51	410.10	[M+COOH]^−^	455.09	−8.69	369.10 [M−H−C_2_O]^−^ 175.04 [M−H−C_12_H_10_O_5_]^−^	-	Coumarins and their derivatives
92	Heptyl 6-*O*-α-L -arabinopyranosyl-β-D-glucopyranoside	C_18_H_34_O_10_	3.55	410.22	[M+COOH]^−^	455.21	−1.60	395.19 [M−H−CH_2_]^−^ 263.15 [M−H−C_6_H_10_O_4_]^−^	-	Carbohydrates and their derivatives
93	(+)-Isolariciresinol 9’-*O*-glucoside	C_26_H_34_O_11_	3.64	522.21	[M+COOH]^−^	567.21	0.43	329.14 [M−H−C_7_H_12_O_6_]^−^ 191.06 [M−H−C_19_H_22_O_5_]^−^ 161.05 [M−H−C_20_H_24_O_6_]^−^	63358-12-3	Carbohydrates and their derivatives
94	4-(3-hydroxy-1-butenyl) -3,5,5-trimethyl-2-cyclohexanone	C_13_H_22_O_2_	3.65	210.16	[M+H]^+^	211.17	−1.22	193.16 [M+H−H_2_O]^+^ 151.11 [M+H−C_3_H_8_O]^+^ 137.10 [M+H−C_4_H_10_O]^+^	-	Others
95	Cuneataside E	C_24_H_40_O_11_	3.65	504.26	[M+COOH]^−^	549.26	−0.36	429.18 [M−H−C_4_H_10_O]^−^ 371.21 [M−H−C_5_H_8_O_4_]^−^ 209.15 [M−H−C_11_H_18_O_9_]^−^ 161.05 [M−H−C_18_H_30_O_6_]^−^ 143.04 [M−H−C_18_H_32_O_7_]^−^	-	Terpenoids and their derivatives
96	Matairesinoside	C_26_H_32_O_11_	3.69	520.19	[M+COOH]^−^	565.19	−0.26	339.12 [M−H−C_6_H_12_O_6_]^−^ 179.06 [M−H−C_20_H_20_O_5_]^−^ 121.03 [M−H−C_19_H_26_O_9_]^−^	23202-85-9	Carbohydrates and their derivatives
97	Benzoic acid	C_7_H_6_O_2_	3.71	122.04	[M−H]^−^	121.03	−0.04	77.04 [M−H−CO_2_]^−^	65-85-0	Organic acids and their derivatives
98	kaempferide	C_16_H_12_O_6_	3.75	300.06	[M−H]^−^	299.06	−0.72	284.03 [M−H−CH_3_]^−^	491-54-3	Flavonoids and their derivatives
99	Inositol	C_6_H_12_O_6_	3.79	180.06	[M−H]^−^	179.06	−0.52	161.05 [M−H−H_2_O]^−^ 89.02 [M−H−C_3_H_6_O_3_]^−^ 59.01 [M−H−C_4_H_8_O_4_]^−^	87-89-8	Others
100	Apigenin	C_15_H_10_O_5_	3.82	270.05	[M+H]^+^	271.06	1.23	153.02 [M+H−C_8_H_6_O]^+^ 145.03 [M+H−C_6_H_6_O_3_]^+^ 119.05 [M+H−C_7_H_4_O_4_]^+^	520-36-5	Flavonoids and their derivatives
101	Kaempferol 7-*O*-rhamnoside	C_21_H_20_O_10_	3.82	432.11	[M−H]^−^	431.10	−1.38	285.04 [M−H−C_6_H_10_O_4_]^−^ 267.03 [M−H−C_6_H_12_O_5_]^−^ 179.04 [M−H−C_12_H_12_O_6_]^−^ 161.05 [M−H−C_15_H_10_O_5_]^−^ 135.05 [M−H−C_13_H_12_O_8_]^−^	20196-89-8	Flavonoids and their derivatives
102	Homoorientin	C_21_H_20_O_11_	3.83	448.10	[M+H]^+^	449.11	4.27	287.06 [M+H−C_6_H_10_O_5_]^+^ 271.06 [M+H−C_6_H_10_O_6_]^+^	4261-42-1	Flavonoids and their derivatives
103	Diosmetin 7-*O*-beta-D-glucoside	C_22_H_22_O_11_	3.91	462.12	[M−H]^−^	461.11	0.24	446.09 [M−H−CH_3_]^−^ 297.04 [M−H−C_6_H_12_O_5_]^−^ 283.02 [M−H−C_7_H_14_O_5_]^−^	20126-59-4	Flavonoids and their derivatives
104	4-(3-α-hydroxy-1-butenyl)-3,5,5- trimethyl-2-cyclohexen-1-ketone	C_13_H_20_O_2_	4.05	208.15	[M+H]^+^	209.15	0.28	191.14 [M+H−H_2_O]^+^ 137.10 [M+H−C_4_H_8_O]^+^	-	Others
105	Ajugaside A	C_32_H_50_O_14_	4.28	658.32	[M+COOH]^−^	703.32	−1.88	495.26 [M−H−C_6_H_10_O_5_]^−^ 333.21 [M−H−C_12_H_20_O_10_]^−^	213134-86-2	Terpenoids and their derivatives
106	10-epi-Atractyloside A	C_21_H_36_O_10_	4.38	448.23	[M+COOH]^−^	493.23	−0.94	429.21 [M−H−H_2_O]^−^	-	Terpenoids and their derivatives
107	Kaemferitrin	C_27_H_30_O_14_	4.50	578.16	[M−H]^−^	577.16	−1.01	269.05 [M−H−C_12_H_20_O_9_]^−^ 253.05 [M−H−C_12_H_20_O_10_]^−^	482-38-2	Flavonoids and their derivatives
108	Ophiopogoside B	C_26_H_28_O_12_	4.76	532.16	[M−H]^−^	531.15	−0.12	351.09 [M−H−C_6_H_12_O_6_]^−^ 161.05 [M−H−C_20_H_18_O_7_]^−^ 133.03 [M−H−C_18_H_22_O_10_]^−^	-	Flavonoids and their derivatives
109	Luteolin *	C_15_H_10_O_6_	4.77	286.05	[M−H]^−^	285.04	−1.48	133.03 [M−H−C_7_H_4_O_4_]^−^	491-70-3	Flavonoids and their derivatives
110	Lappaol A	C_30_H_32_O_9_	5.15	536.20	[M+COOH]^−^	581.20	−0.91	355.12 [M−H−C_10_H_12_O_3_]^−^ 150.03 [M−H−C_22_H_25_O_6_]^−^	62333-08-8	Organic acids and their derivatives
111	9,12,13,TriHODE	C_18_H_32_O_5_	5.41	328.22	[M−H]^−^	327.22	−2.09	229.14 [M−H−C_6_H_10_O]^−^ 211.13 [M−H−C_6_H_12_O_2_]^−^ 183.14 [M−H−C_7_H_12_O_3_]^−^ 171.10 [M−H−C_9_H_16_O_2_]^−^	-	Organic acids and their derivatives
112	9,10,13-Trihydroxy-11-octadecenoic acid	C_18_H_34_O_5_	5.77	330.24	[M−H]^−^	329.23	−1.30	311.22 [M−H−H_2_O]^−^ 229.14 [M−H−C_6_H_12_O]^−^ 211.13 [M−H−C_6_H_14_O_2_]^−^ 171.10 [M−H−C_9_H_18_O_2_]^−^ 127.11 [M−H−C_10_H_18_O_4_]^−^	29907-57-1	Organic acids and their derivatives
113	Euscaphic acid	C_30_H_48_O_5_	6.52	488.35	[M+COOH]^−^	533.35	0.82	183.14 [M−H−C_19_H_28_O_3_]^−^	53155-25-2	Terpenoids and their derivatives
114	Interiotherin A	C_29_H_28_O_8_	6.65	504.18	[M−H]^−^	503.17	−0.27	367.12 [M−H−C_8_H_8_O_2_]^−^ 150.03 [M−H−C_21_H_21_O_5_]^−^	181701-06-4	Others
115	8-Nonenoic acid	C_9_H_16_O_2_	7.05	156.12	[M+COOH]^−^	201.11	−0.64	137.10 [M−H−H_2_O]^−^	31642-67-8	Organic acids and their derivatives
116	Dehydrophytosphingosine	C_18_H_37_NO_3_	7.17	315.28	[M+H]^+^	316.28	0.14	298.27 [M+H−H_2_O]^+^ 280.26 [M+H−2H_2_O]^+^	3687-54-5	Organonitrogen compounds and their derivatives
117	Oleanolic acid	C_30_H_48_O_3_	7.47	456.36	[M+H]^+^	457.37	1.90	421.35 [M+H−2H_2_O]^+^	508-02-1	Terpenoids and their derivatives
118	1,18-Octadec-9-enedioic acid	C_18_H_32_O_4_	7.52	312.23	[M+Na]^+^	335.22	−0.31	277.22 [M+H−2H_2_O]^−^ 109.10 [M+H−C_10_H_20_O_4_]^−^	20701-67-1	Organic acids and their derivatives
119	9-HOTrE	C_18_H_30_O_3_	7.52	294.22	[M−H]^−^	293.21	−2.20	275.20 [M−H−H_2_O]^−^ 185.12 [M−H−C_8_H_12_]^−^ 171.10 [M−H−C_9_H_14_]^−^ 167.11 [M−H−C_8_H_14_O]^−^	89886-42-0	Organic acids and their derivatives
120	(5R)-1-(3,4-Dimethoxyphenyl)-5-hydroxydecan-3-one	C_18_H_28_O_4_	7.54	308.20	[M−H]^−^	307.19	−1.33	165.09 [M−H−C_8_H_14_O_2_]^−^ 127.11 [M−H−C_10_H_12_O_3_]^−^	-	Others
121	Octadecanedioic acid	C_18_H_34_O_4_	7.69	314.25	[M−H]^−^	313.24	−9.74	277.22 [M−H−2H_2_O]^−^	871-70-5	Organic acids and their derivatives
122	9-Hydroperoxy-10,12,15-octadecatrienoic acid	C_18_H_30_O_4_	8.22	310.21	[M+Na]^+^	333.20	−4.04	293.21 [M+H−H_2_O]^+^ 121.10 [M+H−C_9_H_18_O_4_]^+^ 109.10 [M+H−C_10_H_18_O_4_]^+^ 107.09 [M+H−C_10_H_20_O_4_]^+^ 97.03 [M+H−C_13_H_26_O_2_]^+^ 81.07 [M+H−C_12_H_22_O_4_]^+^	111004-08-1	Organic acids and their derivatives
123	1-Monolinolein *	C_21_H_38_O_4_	8.58	354.28	[M+H]^+^	355.28	0.04	337.27 [M+H−H_2_O]^+^ 263.24 [M+H−C_3_H_8_O_3_]^+^ 81.07 [M+H−C_15_H_30_O_4_]^+^	2277-28-3	Lipids and their derivatives
124	Gingerglycolipid B	C_33_H_58_O_14_	8.58	678.38	[M+COOH]^−^	723.38	0.50	415.15 [M−H−C_18_H_30_O]^−^ 397.14 [M−H−C_18_H_32_O_2_]^−^ 309.21 [M−H−C_15_H_28_O_10_]^−^	88168-90-5	Carbohydrates and their derivatives
125	9R-Hydroxy-10E,12Z-octadecadienoic acid	C_18_H_32_O_3_	8.72	296.24	[M−H]^−^	295.23	−0.62	277.22 [M−H−H_2_O]^−^ 195.14 [M−H−C_6_H_12_O]^−^ 171.10 [M−H−C_9_H_16_]^−^	10075-11-3	Organic acids and their derivatives
126	Echinocystic acid	C_30_H_48_O_4_	8.77	472.36	[M−H]^−^	471.35	0.35	453.34 [M−H−H_2_O]^−^ 407.33 [M−H−CH_4_O_3_]^−^	510-30-5	Terpenoids and their derivatives
127	Dibutyl phthalate	C_16_H_22_O_4_	9.00	278.15	[M+Na]^+^	301.14	1.62	149.02 [M+H−C_8_H_18_O]^+^	84-74-2	Lipids and their derivatives
128	1-Palmitoyl-galactosylglycerol	C_25_H_48_O_9_	9.45	492.33	[M+Na]^+^	515.32	−6.94	313.27 [M+H−C_6_H_12_O_6_]^+^	-	Lipids and their derivatives
129	12-Hydroxypentanoic acid methyl ester	C_15_H_30_O_3_	9.74	258.22	[M−H]^−^	257.21	−0.92	211.21 [M−H−CH_2_O_2_]^−^	-	Lipids and their derivatives
130	Linolenic acid	C_18_H_30_O_2_	10.14	278.22	[M−H]^−^	277.22	−0.92	235.17 [M−H−C_3_H_6_]^−^ 195.14 [M−H−C_6_H_10_]^−^	463-40-1	Organic acids and their derivatives
131	7-Keto-β-Sitosterol	C_29_H_48_O_2_	10.64	428.37	[M+H]^+^	429.37	1.68	411.36 [M+H−H_2_O]^+^ 313.22 [M+H−C_8_H_20_]^+^ 109.06 [M+H−C_22_H_40_O]^+^	2034-74-4	Steroids and their derivatives
132	Linoleic acid	C_18_H_32_O_2_	10.64	280.24	[M−H]^−^	279.23	−1.97	261.22 [M−H−H_2_O]^−^ 189.13 [M−H−C_5_H_14_O]^−^ 163.11 [M−H−C_7_H_16_O]^−^ 137.10 [M−H−C_9_H_18_O]^−^	60-33-3	Organic acids and their derivatives
133	9(Z)-Octadecenamide	C_18_H_35_NO	10.77	281.27	[M+H]^+^	282.28	3.78	95.09 [M+H−C_11_H_25_NO]^+^ 86.10 [M+H−C_13_H_24_O]^+^ 81.07 [M+H−C_12_H_27_NO]^+^ 67.05 [M+H−C_13_H_29_NO]^+^	301-02-0	Organonitrogen compounds and their derivatives

The symbol ‘*’ indicates compounds were identified using standard substances, the remaining compounds were identified by fragmentation regularities.

**Table 2:** *Girardinia diversifolia* chemical compounds identification.

NO.	Compound	Formula	Observed RT (min)	Molecular Mass	Ionization Model	Observed *m*/*z*	Mass Error (ppm)	Fragment Ions	CAS	Class
1	Gluconic acid	C_6_H_12_O_7_	0.56	196.06	[M−H]^−^	195.05	−1.45	177.04 [M−H−H_2_O]^−^ 165.04 [M−H−CH_2_O]^−^ 105.02 [M−H−C_3_H_6_O_3_]^−^	526-95-4	Organic acids and their derivatives
2	2-*O*-alpha-D-Glucopyranosyl-L-ascorbic acid *	C_12_H_18_O_11_	0.60	338.08	[M−H]^−^	337.08	−0.52	175.02 [M−H−C_6_H_10_O_5_]^−^ 157.01 [M−H−C_6_H_12_O_6_]^−^	129499-78-1	Carbohydrates and their derivatives
3	Malic acid	C_4_H_6_O_5_	0.61	134.02	[M−H]^−^	133.01	−2.19	115.00 [M−H−H_2_O]^−^ 72.99 [M−H−C_2_H_4_O_2_]^−^	6915-15-7	Organic acids and their derivatives
4	9-Ribofuranosyladenine *	C_10_H_13_N_5_O_4_	1.04	267.10	[M+H]^+^	268.10	2.03	136.06 [M+H−C_5_H_8_O_4_]^+^ 119.04 [M+H−C_5_H_11_NO_4_]^+^	58-61-7	Organonitrogen compounds and their derivatives
5	Guanosine	C_10_H_13_N_5_O_5_	1.15	283.09	[M−H]^−^	282.08	−1.09	150.04 [M−H−C_5_H_8_O_4_]^−^ 133.02 [M−H−C_5_H_11_NO_4_]^−^	118-00-3	Organonitrogen compounds and their derivatives
6	p-Hydroxybenzoic acid	C_7_H_6_O_3_	1.44	138.03	[M−H]^−^	137.02	1.07	93.03 [M−H−CO_2_]^−^	99-96-7	Organic acids and their derivatives
7	Phenylalanine *	C_9_H_11_NO_2_	1.52	165.08	[M+H]^+^	166.09	−0.34	120.08 [M+H−CH_2_O_2_]^+^ 103.05 [M+H−CH_5_NO_2_]^+^	63-91-2	Organonitrogen compounds and their derivatives
8	2,5-Dihydroxybenzoic acid	C_7_H_6_O_4_	1.66	154.03	[M+H]^+^	155.03	0.79	137.02 [M+H−H_2_O]^+^ 109.03 [M+H−CH_2_O_2_]^+^	490-79-9	Organic acids and their derivatives
9	Protocatechuic acid 3-glucoside *	C_13_H_16_O_9_	1.66	316.08	[M−H]^−^	315.07	−0.46	297.06 [M−H−H_2_O]^−^ 152.01 [M−H−C_6_H_11_O_5_]^−^ 108.02 [M−H−C_7_H_11_O_7_]^−^	20300-54-3	Carbohydrates and their derivatives
10	Danshensu	C_9_H_10_O_5_	1.83	198.05	[M+H]^+^	199.06	−1.67	181.05 [M+H−H_2_O]^+^	76822-21-4	Organic acids and their derivatives
11	Ethyl gallate	C_9_H_10_O_5_	1.83	198.05	[M−H]^−^	197.05	−0.53	153.02 [M−H−C_2_H_4_O]^−^ 123.01 [M−H−C_3_H_6_O_2_]^−^	831-61-8	Lipids and their derivatives
12	c-Veratroylglycol	C_10_H_12_O_5_	1.96	212.07	[M−H]^−^	211.06	0.62	167.04 [M−H−C_2_H_4_O]^−^	168293-10-5	Others
13	1H-indole-2-carbaldehyde	C_9_H_7_NO	2.06	145.05	[M+H]^+^	146.06	−1.34	118.07 [M+H−CO]^+^	19005-93-7	Organonitrogen compounds and their derivatives
14	p-Coumaric acid	C_9_H_8_O_3_	2.37	164.05	[M−H]^−^	163.04	−0.80	119.05 [M−H−CO_2_]^−^ 104.03 [M−H−C_2_H_3_O_2_]^−^	7400-08-0	Organic acids and their derivatives
15	Ferulic acid *	C_10_H_10_O_4_	2.55	194.06	[M−H]^−^	193.05	−2.28	178.03 [M−H−CH_3_]^−^ 149.06 [M−H−CO_2_]^−^ 134.04 [M−H−C_2_H_3_O_2_]^−^	1135-24-6	Organic acids and their derivatives
16	Vitexin 2″−*O*−glucoside	C_27_H_30_O_15_	2.64	594.16	[M−H]^−^	593.15	0.65	473.11 [M−H−C_4_H_8_O_4_]^−^ 383.08 [M−H−C_7_H_14_O_7_]^−^ 129.02 [M−H−C_22_H_24_O_11_]^−^	-	Carbohydrates and their derivatives
17	Vitamin B2	C_17_H_20_N_4_O_6_	2.69	376.14	[M+H]^+^	377.15	0.84	256.15 [M+H−C_4_N_3_O_2_]^+^ 120.08 [M+H−C_9_H_11_N_3_O_6_]^+^	83-88-5	Organonitrogen compounds and their derivatives
18	Dichotomoside A	C_26_H_32_O_13_	2.85	552.18	[M−H]^−^	551.18	−1.95	389.12 [M−H−C_6_H_10_O_5_]^−^ 357.13 [M−H−C_6_H_10_O_7_]^−^	845673-17-8	Organic acids and their derivatives
19	D-Aspartatic acid	C_4_H_7_NO_4_	2.88	133.04	[M−H]^−^	132.03	0.06	88.04 [M−H−CO_2_]^−^	6899-03-2	Organonitrogen compounds and their derivatives
20	Schaftoside	C_26_H_28_O_14_	2.91	564.15	[M+H]^+^	565.16	0.93	547.14 [M+H−H_2_O]^+^ 162.03 [M+H−C_17_H_23_O_11_]^+^	51938-32-0	Flavonoids and their derivatives
21	Vitexin 2″-*O*-xyloside	C_26_H_28_O_14_	2.92	564.15	[M−H]^−^	563.14	−0.30	545.13 [M−H−H_2_O]^−^ 383.08 [M−H−C_6_H_12_O_6_]^−^	-	Carbohydrates and their derivatives
22	4-Hydroxy-3-methoxycinnamaldehyde	C_10_H_10_O_3_	2.97	178.06	[M+H]^+^	179.07	−2.02	161.06 [M+H−H_2_O]^+^ 151.08 [M+H−CO]^+^ 135.04 [M+H−C_2_H_4_O]^+^ 107.05 [M+H−C_3_H_4_O_2_]^+^	458-36-6	Others
23	3,5,6,8,3′,4′,5′-Heptamethoxyflavone	C_22_H_24_O_9_	3.06	432.14	[M+COOH]^−^	477.14	−0.69	371.11 [M−H−C_2_H_4_O_2_]^−^ 208.04 [M−H−C_12_H_15_O_4_]^−^ 149.02 [M−H−C_14_H_18_O_6_]^−^	-	Flavonoids and their derivatives
24	Eleutheroside B	C_17_H_24_O_9_	3.18	372.14	[M+COOH]^−^	417.14	−1.85	209.08 [M−H−C_6_H_10_O_5_]^−^ 193.05 [M−H−C_7_H_14_O_5_]^−^	118-34-3	Carbohydrates and their derivatives
25	Kaempferol-3-*O*-β-D-glucopyranoside	C_21_H_20_O_11_	3.44	448.10	[M−H]^−^	447.09	−0.48	285.04 [M−H−C_6_H_10_O_5_]^−^ 283.02 [M−H−C_6_H_12_O_5_]^−^ 227.04 [M−H−C_8_H_12_O_7_]^−^ 151.00 [M−H−C_14_H_16_O_7_]^−^ 107.01 [M−H−C_15_H_16_O_9_]^−^	480-10-4	Flavonoids and their derivatives
26	Scopoletin	C_10_H_8_O_4_	3.46	192.04	[M+H]^+^	193.05	−0.33	178.03 [M+H−CH_3_]^+^ 122.04 [M+H−C_3_H_3_O_2_]^+^	92-61-5	Coumarins and their derivatives
27	(+)-Isolariciresinol 9′-*O*-glucoside	C_26_H_34_O_11_	3.50	522.21	[M−H]^−^	521.20	1.07	399.17 [M−H−C_7_H_6_O_2_]^−^ 329.14 [M−H−C_7_H_12_O_6_]^−^	63358-12-3	Carbohydrates and their derivatives
28	Denudanolide B	C_20_H_22_O_6_	3.62	358.14	[M−H]^−^	357.14	2.30	339.12 [M−H−H_2_O]^−^ 312.10 [M−H−C_2_H_5_O]^−^	-	Others
29	Caffeic acid cinnamyl ester	C_18_H_16_O_4_	3.39	296.10	[M+COOH]^−^	341.10	1.30	205.05 [M−H−C_7_H_6_]^−^ 163.04 [M−H−C_9_H_8_O]^−^	-	Lipids and their derivatives
30	(+)-Pinoresinol 4-*O*-glucoside	C_26_H_32_O_11_	3.59	520.19	[M−H]^−^	519.19	−2.94	165.06 [M−H−C_17_H_22_O_8_]^−^ 151.04 [M−H−C_18_H_24_O_8_]^−^	69251-96-3	Carbohydrates and their derivatives
31	Benzoic acid	C_7_H_6_O_2_	3.71	122.04	[M−H]^−^	121.03	−0.12	77.04 [M−H−CO_2_]^−^	65-85-0	Organic acids and their derivatives
32	2-(4-Hydroxy-3-methoxy-phenyl)-5-(3-hydroxy-propyl)-7-methoxy-benzofuran-3-carbaldehyde	C_20_H_20_O_6_	3.78	356.13	[M+H]^+^	357.13	−0.88	293.08 [M+H−C_2_H_8_O_2_]^+^ 177.05 [M+H−C_10_H_12_O_3_]^+^	135040-83-4	Flavonoids and their derivatives
33	Apigenin	C_15_H_10_O_5_	3.82	270.05	[M+H]^+^	271.06	0.70	153.02 [M+H−C_8_H_6_O]^+^	520-36-5	Flavonoids and their derivatives
34	Vitexin	C_21_H_20_O_10_	3.82	432.11	[M−H]^−^	431.10	−0.37	267.03 [M−H−C_6_H_12_O_5_]^−^	3681-93-4	Flavonoids and their derivatives
35	5alpha-Hydroxytriptonide	C_20_H_22_O_7_	4.00	374.14	[M−H]^−^	373.13	−2.23	355.12 [M−H−H_2_O]^−^ 193.05 [M−H−C_10_H_12_O_3_]^−^ 179.07 [M−H−C_10_H_10_O_4_]^−^ 164.05 [M−H−C_11_H_13_O_4_]^−^	-	Others
36	Melianoninol	C_20_H_20_O_6_	4.06	356.13	[M−H]^−^	355.12	0.29	337.11 [M−H−H_2_O]^−^ 327.12 [M−H−CO]^−^ 325.11 [M−H−CH_2_O]^−^ 306.09 [M−H−CH_5_O_2_]^−^	136880-81-4	Others
37	(+)-Cabralealactone	C_27_H_42_O_3_	4.23	414.31	[M+Na]^+^	437.31	8.50	219.17 [M+H−C_12_H_20_O_2_]^+^ 205.16 [M+H−C_13_H_22_O_2_]^+^ 137.10 [M+H−C_18_H_30_O_2_]^+^	19865-87-3	Terpenoids and their derivatives
38	Gossypetin hexamethyl ether	C_21_H_22_O_8_	4.32	402.13	[M+COOH]^−^	447.13	−0.87	208.04 [M−H−C_11_H_13_O_3_]^−^	7741-47-1	Flavonoids and their derivatives
39	Kinobeon A	C_20_H_20_O_6_	4.56	356.13	[M+COOH]^−^	401.12	−0.08	177.06 [M−H−C_10_H_10_O_3_]^−^ 162.03 [M−H−C_11_H_13_O_3_]^−^ 148.05 [M−H−C_11_H_11_O_4_]^−^ 134.04 [M−H−C_12_H_13_O_4_]^−^	155239-87-5	Others
40	Dumetorine	C_13_H_21_NO_2_	4.70	223.16	[M+H]^+^	224.16	0.39	140.11 [M+H−C_5_H_8_O]^+^ 83.05 [M+H−C_8_H_15_NO]^+^ 82.07 [M+H−C_8_H_14_O_2_]^+^	96552-67-9	Organonitrogen compounds and their derivatives
41	(E,7R)-7-Hydroxy-1,7-bis(4-hydroxy-3-methoxyphenyl)hept-1-ene-3,5-dione	C_21_H_22_O_7_	4.73	386.14	[M+COOH]^−^	431.14	2.00	207.07 [M−H−C_10_H_10_O_3_]^−^ 192.04 [M−H−C_11_H_13_O_3_]^−^ 177.02 [M−H−C_12_H_16_O_3_]^−^	-	Others
42	Luteolin *	C_15_H_10_O_6_	4.77	286.05	[M−H]^−^	285.04	0.02	133.03 [M−H−C_7_H_4_O_4_]^−^	491-70-3	Flavonoids and their derivatives
43	1,4-Bis(benzoyloxy)butane	C_18_H_18_O_4_	4.86	298.12	[M+Na]^+^	321.11	9.70	165.09 [M+H−C_8_H_6_O_2_]^+^	19224-27-2	Others
44	Methyl-trans-4-hydroxycinnamate	C_10_H_10_O_3_	4.87	178.06	[M−H]^−^	177.06	−0.59	117.03 [M−H−C_2_H_4_O_2_]−	19367-38-5	Lipids and their derivatives
45	Lappaol A	C_30_H_32_O_9_	5.15	536.20	[M+COOH]^−^	581.20	0.15	367.12 [M−H−C_9_H_12_O_3_]^−^ 150.03 [M−H−C_22_H_25_O_6_]^−^ 119.05 [M−H−C_22_H_24_O_8_]^−^	62333-08-8	Organic acids and their derivatives
46	9,12,13,TriHODE	C_18_H_32_O_5_	5.41	328.22	[M−H]^−^	327.22	−1.63	229.14 [M−H−C_6_H_10_O]^−^ 211.13 [M−H−C_6_H_12_O_2_]^−^ 171.10 [M−H−C_9_H_16_O_2_]^−^	-	Organic acids and their derivatives
47	Esculentoside B	C_36_H_56_O_11_	5.73	664.38	[M−H]^−^	663.37	−0.48	515.30 [M−H−C_6_H_12_O_4_]^−^ 501.32 [M−H−C_6_H_10_O_5_]^−^ 487.34 [M−H−C_6_H_8_O_6_]^−^ 467.32 [M−H−C_6_H_12_O_7_]^−^ 453.30 [M−H−C_7_H_14_O_7_]^−^	60820-94-2	Terpenoids and their derivatives
48	9,10,13-Trihydroxy-11-octadecenoic acid	C_18_H_34_O_5_	5.76	330.24	[M−H]^−^	329.23	−1.18	311.22 [M−H−H_2_O]^−^ 293.21 [M−H−2H_2_O]^−^ 229.14 [M−H−C_6_H_12_O]^−^ 211.13 [M−H−C_6_H_14_O_2_]^−^ 171.10 [M−H−C_9_H_18_O_2_]^−^	29907-57-1	Organic acids and their derivatives
49	Cyclocurcumin	C_21_H_20_O_6_	6.15	368.13	[M+H]^+^	369.13	−1.28	351.12 [M+H−H_2_O]^+^ 337.11 [M+H−CH_4_O]^+^	153127-42-5	Others
50	Ganoderic acid C2	C_30_H_46_O_7_	6.32	518.32	[M−H]^−^	517.32	−1.51	499.31 [M−H−H_2_O]^−^ 473.29 [M−H−C_2_H_4_O]^−^ 455.32 [M−H−CH_2_O_3_]^−^ 429.30 [M−H−C_3_H_4_O_3_]^−^	103773-62-2	Terpenoids and their derivatives
51	Roxburic acid	C_30_H_48_O_6_	6.34	504.35	[M−H]^−^	503.34	0.04	485.33 [M−H−H_2_O]^−^ 467.32 [M−H−2H_2_O]^−^ 441.34 [M−H−CH_2_O_3_]^−^	108657-25-6	Terpenoids and their derivatives
52	Interiotherin A	C_29_H_28_O_8_	6.65	504.18	[M−H]^−^	503.17	0.06	367.12 [M−H−C_8_H_8_O_2_]^−^ 150.03 [M−H−C_21_H_21_O_5_]^−^	181701-06-4	Others
53	Antheridic acid	C_19_H_22_O_6_	6.66	346.14	[M+Na]^+^	369.13	5.17	311.13 [M+H−2H_2_O]^+^	34327-25-8	Organic acids and their derivatives
54	Asiatic acid	C_30_H_48_O_5_	6.94	488.35	[M−H]^−^	487.34	−1.49	441.34 [M−H−CH_2_O_2_]^−^ 427.32 [M−H−C_2_H_4_O_2_]^−^ 425.34 [M−H−CH_2_O_3_]^−^	464-92-6	Terpenoids and their derivatives
55	Dehydrophytosphingosine	C_18_H_37_NO_3_	7.17	315.28	[M+H]^+^	316.28	0.78	298.27 [M+H−H_2_O]^+^ 280.26 [M+H−2H_2_O]^+^	3687-54-5	Organonitrogen compounds and their derivatives
56	1,18-Octadec-9-enedioic acid	C_18_H_32_O_4_	7.53	312.23	[M+Na]^+^	335.22	−0.23	109.10 [M+H−C_10_H_20_O_4_]^+^ 95.09 [M+H−C_11_H_22_O_4_]^+^	20701-67-1	Organic acids and their derivatives
57	tormentic acid	C_30_H_48_O_5_	7.54	488.35	[M−H]^−^	487.34	−0.64	469.33 [M−H−H_2_O]^−^ 441.34 [M−H−CH_2_O_2_]^−^ 427.32 [M−H−C_2_H_4_O_2_]^−^ 171.10 [M−H−C_21_H_32_O_2_]^−^	13850-16-3	Terpenoids and their derivatives
58	Octadecanedioic acid	C_18_H_34_O_4_	7.68	314.25	[M−H]^−^	313.24	−4.02	277.22 [M−H−2H_2_O]^−^	871-70-5	Organic acids and their derivatives
59	6-Methylgingediol	C_18_H_30_O_4_	8.14	310.21	[M+Na]^+^	333.20	−0.32	293.21 [M+H−H_2_O]^+^ 165.09 [M+H−C_8_H_18_O_2_]^+^	-	Others
60	9-Hydroperoxy-10,12,15-octadecatrienoic acid	C_18_H_30_O_4_	8.22	310.21	[M+Na]^+^	333.20	−1.46	293.21 [M+H−H_2_O]^+^ 125.10 [M+H−C_10_H_18_O_3_]^+^ 107.09 [M+H−C_10_H_20_O_4_]^+^ 95.09 [M+H−C_11_H_20_O_4_]^+^	111004-08-1	Organic acids and their derivatives
61	1-Monolinolein *	C_21_H_38_O_4_	8.58	354.28	[M+H]^+^	355.28	0.45	337.27 [M+H−H_2_O]^+^ 311.26 [M+H−C_2_H_4_O]^+^ 263.24 [M+H−C_3_H_8_O_3_]^+^	2277-28-3	Lipids and their derivatives
62	Gingerglycolipid B	C_33_H_58_O_14_	8.58	678.38	[M+COOH]^−^	723.38	1.48	415.15 [M−H−C_18_H_30_O]^−^ 397.14 [M−H−C_18_H_32_O_2_]^−^	88168-90-5	Carbohydrates and their derivatives
63	9R-Hydroxy-10E,12Z-octadecadienoic acid	C_18_H_32_O_3_	8.72	296.24	[M−H]^−^	295.23	−1.03	277.22 [M−H−H_2_O]^−^ 249.22 [M−H−CH_2_O_2_]^−^ 195.14 [M−H−C_6_H_12_O]^−^ 171.10 [M−H−C_9_H_16_]^−^	10075-11-3	Organic acids and their derivatives
64	Sumaresinolic acid	C_30_H_48_O_4_	8.77	472.36	[M−H]^−^	471.35	−0.92	453.34 [M−H−H_2_O]^−^	559-64-8	Terpenoids and their derivatives
65	Nigranoic acid	C_30_H_46_O_4_	8.13	470.34	[M−H]^−^	469.33	−1.21	455.32 [M−H−CH_2_]^−^ 411.33 [M−H−C_2_H_2_O_2_]^−^ 395.30 [M−H−C_3_H_6_O_2_]^−^	39111-07-4	Organic acids and their derivatives
66	9-oxooctadeca-10,12-dienoic acid	C_18_H_30_O_3_	8.99	294.22	[M−H]^−^	293.21	−2.60	275.20 [M−H−H_2_O]^−^ 171.10 [M−H−C_9_H_14_]^−^	54232-58-5	Organic acids and their derivatives
67	Dibutyl phthalate	C_16_H_22_O_4_	9.01	278.15	[M+Na]^+^	301.14	0.91	177.05 [M+H−C_6_H_14_O]^+^ 149.02 [M+H−C_8_H_18_O]^+^	84-74-2	Lipids and their derivatives
68	CAULOPHYLLOGENIN	C_30_H_48_O_5_	9.08	488.35	[M+COOH]^−^	533.35	−0.27	469.33 [M−H−H_2_O]^−^ 439.32 [M−H−CH_4_O_2_]^−^ 253.22 [M−H−C_14_H_18_O_3_]^−^	52936-64-8	Terpenoids and their derivatives
69	Mudanpinoic acid A	C_30_H_46_O_3_	9.26	454.34	[M+H]^+^	455.35	−1.88	409.35 [M+H−CH_2_O_2_]^+^	203511-36-8	Steroids and their derivatives
70	12-Hydroxypentanoic acid methyl ester	C_15_H_30_O_3_	9.74	258.22	[M−H]^−^	257.21	−2.74	211.21 [M−H−CH_2_O_2_]^−^	-	Lipids and their derivatives
71	Bis(2-propylpentyl) phthalate	C_24_H_38_O_4_	10.01	390.28	[M+Na]^+^	413.27	1.36	149.02 [M+H−C_16_H_34_O]^+^	70910-37-1	Others
72	16-O-Acetyldarutigenol	C_22_H_36_O_4_	10.28	364.26	[M+Na]^+^	387.25	3.39	261.22 [M+H−C_4_H_8_O_3_]^+^	1188282-01-0	Terpenoids and their derivatives
73	Incensole oxide	C_20_H_34_O_3_	10.35	322.25	[M+Na]^+^	345.24	0.27	263.20 [M+H−C_3_H_8_O]^+^	21698-66-8	Lipids and their derivatives
74	Brassicasterol	C_28_H_46_O	10.63	398.35	[M+H]^+^	399.36	−1.33	261.22 [M+H−C_10_H_18_]^+^ 205.20 [M+H−C_13_H_22_O]^+^ 179.14 [M+H−C_16_H_28_]^+^ 109.10 [M+H−C_20_H_34_O]^+^	474-67-9	Steroids and their derivatives
75	Caffeic acid docosanoyl ester	C_31_H_50_O_5_	10.63	502.37	[M−H]^−^	501.36	−1.39	391.32 [M−H−C_6_H_6_O_2_]^−^ 279.23 [M−H−C_13_H_18_O_3_]^−^	-	Lipids and their derivatives
76	7-Keto-β-Sitosterol	C_29_H_48_O_2_	10.64	428.37	[M+H]^+^	429.37	1.03	411.36 [M+H−H_2_O]^+^ 275.20 [M+H−C_11_H_22_]^+^	2034-74-4	Steroids and their derivatives
77	9(Z)-Octadecenamide	C_18_H_35_NO	10.77	281.27	[M+H]^+^	282.28	4.03	95.09 [M+H−C_11_H_25_NO]^+^ 86.10 [M+H−C_13_H_24_O]^+^ 81.07 [M+H−C_12_H_27_NO]^+^	301-02-0	Organonitrogen compounds and their derivatives

The symbol ‘*’ indicates compounds were identified using standard substances, the remaining compounds were identified by fragmentation regularities.

**Table 3:** *Urtica mairei* chemical compounds identification.

NO.	Compound	Formula	Observed RT (min)	Molecular Mass	Ionization Model	Observed *m*/*z*	Mass Error (ppm)	Fragment Ions	CAS	Class
1	Gluconic acid	C_6_H_12_O_7_	0.55	196.06	[M−H]^−^	195.05	−3.67	177.04 [M−H−H_2_O]^−^ 165.04 [M−H−CH_2_O]^−^ 159.03 [M−H−2H_2_O]^−^ 105.02 [M−H−C_3_H_6_O_3_]^−^	526-95-4	Organic acids and their derivatives
2	D-Raffinose	C_18_H_32_O_16_	0.56	504.17	[M+Cl]^−^	539.14	2.78	341.11 [M−H−C_6_H_10_O_5_]^−^ 179.06 [M−H−C_12_H_20_O_10_]^−^ 161.05 [M−H−C_12_H_22_O_11_]^−^ 143.04 [M−H−C_12_H_24_O_12_]^−^	512-69-6	Carbohydrates and their derivatives
3	L-Proline	C_5_H_9_NO_2_	0.58	115.06	[M+H]^+^	116.07	1.45	85.03 [M+H−CH_5_N]^+^ 70.07 [M+H−CH_2_O_2_]^+^	147-85-3	Organonitrogen compounds and their derivatives
4	Malic acid	C_4_H_6_O_5_	0.61	134.02	[M−H]^−^	133.01	−3.44	115.00 [M−H−H_2_O]^−^ 72.99 [M−H−C_2_H_4_O_2_]^−^	6915-15-7	Organic acids and their derivatives
5	Citric acid *	C_6_H_8_O_7_	0.66	192.03	[M−H]^−^	191.02	−3.14	173.01 [M−H−H_2_O]^−^ 129.02 [M−H−CH_2_O_3_]^−^ 87.01 [M−H−C_3_H_4_O_4_]^−^	77-92-9	Organic acids and their derivatives
6	9-Ribofuranosyladenine *	C_10_H_13_N_5_O_4_	1.04	267.10	[M+H]^+^	268.10	1.32	136.06 [M+H−C_5_H_8_O_4_]^+^ 119.04 [M+H−C_5_H_11_NO_4_]^+^	58-61-7	Organonitrogen compounds and their derivatives
7	Succinic acid	C_4_H_6_O_4_	1.06	118.03	[M−H]^−^	117.02	−0.40	73.03 [M−H−CO_2_]^−^	110-15-6	Organic acids and their derivatives
8	Guanosine	C_10_H_13_N_5_O_5_	1.15	283.09	[M−H]^−^	282.08	−0.35	150.04 [M−H−C_5_H_8_O_4_]^−^ 133.02 [M−H−C_5_H_11_NO_4_]^−^	118-00-3	Organonitrogen compounds and their derivatives
9	p-Hydroxybenzoic acid	C_7_H_6_O_3_	1.43	138.03	[M−H]^−^	137.02	2.71	93.03 [M−H−CO_2_]^−^	99-96-7	Organic acids and their derivatives
10	Phenylalanine *	C_9_H_11_NO_2_	1.52	165.08	[M+H]^+^	166.09	−0.72	120.08 [M+H−CH_2_O_2_]^+^ 103.05 [M+H−CH_5_NO_2_]^+^ 77.04 [M+H−C_3_H_7_NO_2_]^+^	63-91-2	Organonitrogen compounds and their derivatives
11	Protocatechuic acid 3-glucoside	C_13_H_16_O_9_	1.66	316.08	[M−H]^−^	315.07	−1.71	152.01 [M−H−C_6_H_11_O_5_]^−^ 108.02 [M−H−C_7_H_11_O_7_]^−^	20300-54-3	Carbohydrates and their derivatives
12	Vanillin	C_8_H_8_O_3_	1.83	152.05	[M+COOH]^−^	197.05	−1.56	109.03 [M−H−C_2_H_2_O]^−^	121-33-5	Others
13	1H-indole-2-carbaldehyde	C_9_H_7_NO	2.06	145.05	[M+H]^+^	146.06	−0.75	118.07 [M+H−CO]^+^	19005-93-7	Organonitrogen compounds and their derivatives
14	Esculin	C_15_H_16_O_9_	2.09	340.08	[M−H]^−^	339.07	0.45	177.02 [M−H−C_6_H_10_O_5_]^−^	531-75-9	Coumarins and their derivatives
15	Diosmin	C_28_H_32_O_15_	2.49	608.17	[M+H]^+^	609.18	0.44	301.07 [M+H−C_12_H_20_O_9_]^+^ 286.05 [M+H−C_13_H_23_O_9_]^+^	520-27-4	Flavonoids and their derivatives
16	Nicotiflorin	C_27_H_30_O_15_	2.64	594.16	[M+H]^+^	595.17	0.24	559.14 [M+H−2H_2_O]^+^ 253.13 [M+H−C_17_H_10_O_8_]^+^	17650-84-9	Flavonoids and their derivatives
17	Vitexin 2″-*O*-glucoside	C_27_H_30_O_15_	2.64	594.16	[M−H]^−^	593.15	0.39	575.14 [M−H−H_2_O]^−^ 473.11 [M−H−C_4_H_8_O_4_]^−^ 383.08 [M−H−C_7_H_14_O_7_]^−^	-	Carbohydrates and their derivatives
18	Apiopaeonoside	C_20_H_28_O_12_	2.71	460.16	[M−H]^−^	459.15	0.87	310.11 [M−H−C_5_H_9_O_5_]^−^ 151.04 [M−H−C_12_H_20_O_9_]^−^ 121.03 [M−H−C_13_H_22_O_10_]^−^	100291-86-9	Carbohydrates and their derivatives
19	Schaftoside	C_26_H_28_O_14_	2.91	564.15	[M+H]^+^	565.16	1.63	529.13 [M+H−2H_2_O]^+^ 93.03 [M+H−C_20_H_24_O_13_]^+^	51938-32-0	Flavonoids and their derivatives
20	Vitexin 2″-*O*-xyloside	C_26_H_28_O_14_	2.91	564.15	[M−H]^−^	563.14	−0.19	545.13 [M−H−H_2_O]^−^ 383.08 [M−H−C_6_H_12_O_6_]^−^	-	Carbohydrates and their derivatives
21	5-*O*-Feruloylquinic acid	C_17_H_20_O_9_	2.95	368.11	[M+Na]^+^	391.10	3.12	204.06 [M+H−C_9_H_9_O_3_]^+^ 177.05 [M+H−C_7_H_12_O_6_]^+^ 145.03 [M+H−C_8_H_16_O_7_]^+^	40242-06-6	Flavonoids and their derivatives
22	2-(2-pentenyl) -3-methyl-4-hydroxy-2-pentenyl-1-ketone	C_11_H_16_O_2_	3.03	180.12	[M+COOH]^−^	225.11	0.08	149.06 [M−H−C_2_H_6_]^−^ 122.04 [M−H−C_4_H_9_]^−^	-	Others
23	3-Hexenyl-beta-glucopyranoside	C_12_H_22_O_6_	3.18	262.14	[M+COOH]^−^	307.14	−0.97	191.06 [M−H−C_5_H_10_]^−^ 119.04 [M−H−C_8_H_14_O_2_]^−^ 101.02 [M−H−C_8_H_16_O_3_]^−^	95632-87-4	Carbohydrates and their derivatives
24	4-*O*-Feruloylquinic acid	C_17_H_20_O_9_	3.19	368.11	[M−H]^−^	367.10	1.46	191.06 [M−H−C_10_H_8_O_3_]^−^ 177.06 [M−H−C_7_H_10_O_6_]^−^ 71.01 [M−H−C_14_H_16_O_7_]^−^	2613-86-7	Organic acids and their derivatives
25	Citrusin B	C_27_H_36_O_13_	3.21	568.22	[M+Na]^+^	591.20	0.31	207.07 [M+H−C_16_H_26_O_9_]^+^ 179.07 [M+H−C_17_H_26_O_10_]^+^ 163.08 [M+H−C_17_H_26_O_11_]^+^ 137.06 [M+H−C_19_H_28_O_11_]^+^	105279-10-5	Carbohydrates and their derivatives
26	1-beta-Glucogeniposide	C_17_H_26_O_10_	3.22	390.15	[M+COOH]^−^	435.15	6.12	210.05 [M−H−C_7_H_15_O_5_]^−^ 177.06 [M−H−C_7_H_16_O_7_]^−^	-	Carbohydrates and their derivatives
27	5,6,7-Trihydroxyflavone	C_15_H_10_O5	3.22	270.05	[M+H]+	271.06	2.83	131.05 [M+H−C_6_H_4_O_4_]^+^ 103.05 [M+H−C_7_H_4_O_5_]^+^	491-67-8	Flavonoids and their derivatives
28	Vicenin 2	C_27_H_30_O_15_	3.42	594.16	[M+H]+	595.17	3.29	271.06 [M+H−C_12_H_20_O_10_]^+^ 149.06 [M+H−C_18_H_22_O_13_]^+^ 121.06 [M+H−C_19_H_22_O_14_]^+^	-	Flavonoids and their derivatives
29	Scopoletin	C_10_H_8_O_4_	3.46	192.04	[M+H]+	193.05	−0.34	178.03 [M+H−CH_3_]^+^ 150.03 [M+H−C_2_H_3_O]^+^ 122.04 [M+H−C_3_H_3_O_2_]^+^	92-61-5	Coumarins and their derivatives
30	Matairesinoside	C_26_H_32_O_11_	3.69	520.19	[M+COOH]^−^	565.19	0.20	339.12 [M−H−C_6_H_12_O_6_]^−^ 324.10 [M−H−C_7_H_15_O_6_]^−^ 179.06 [M−H−C_2_0H_20_O_5_]^−^	23202-85-9	Carbohydrates and their derivatives
31	Scoparone	C_11_H_10_O_4_	3.87	206.06	[M+H]+	207.07	0.03	189.05 [M+H−H_2_O]^+^ 151.04 [M+H−C_3_H_4_O]^+^ 137.06 [M+H−C_3_H_2_O_2_]^+^	120-08-1	Coumarins and their derivatives
32	Isoeucommin A	C_27_H_34_O_12_	3.91	550.21	[M−H]^−^	549.20	−3.38	369.13 [M−H−C_6_H_12_O_6_]^−^ 249.11 [M−H−C_13_H_16_O_8_]^−^ 153.06 [M−H−C_19_H_24_O_9_]^−^	-	Carbohydrates and their derivatives
33	Bruceine C	C_28_H_36_O_12_	4.37	564.22	[M+Na]^+^	587.21	0.83	467.15 [M+H−C_6_H_10_O]^+^ 151.08 [M+H−C_19_H_26_O_10_]^+^	25514-30-1	Terpenoids and their derivatives
34	Thujaplicatin methyl ether	C_21_H_24_O_7_	4.51	388.15	[M−H]^−^	387.15	0.45	369.13 [M−H−H_2_O]^−^ 357.13 [M−H−CH_2_O]^−^ 342.11 [M−H−C_2_H_5_O]^−^	6512-67-0	Lipids and their derivatives
35	Ophiopogoside B	C_26_H_28_O_12_	4.76	532.16	[M−H]^−^	531.15	0.73	351.09 [M−H−C_6_H_12_O_6_]^−^ 321.08 [M−H−C_7_H_14_O_7_]^−^	-	Flavonoids and their derivatives
36	Lappaol A	C_30_H_32_O_9_	5.15	536.20	[M+COOH]^−^	581.20	0.40	367.12 [M−H−C_9_H_12_O_3_]^−^ 300.10 [M−H−C_13_H_15_O_4_]^−^ 179.07 [M−H−C_20_H_20_O_6_]^−^ 122.04 [M−H−C_23_H_25_O_7_]^−^	62333-08-8	Organic acids and their derivatives
37	9,12,13,TriHODE	C_18_H_32_O_5_	5.41	328.22	[M−H]^−^	327.22	−1.50	291.20 [M−H−2H_2_O]^−^ 229.14 [M−H−C_6_H_1_0O]^−^ 211.13 [M−H−C_6_H_12_O_2_]^−^ 171.10 [M−H−C_9_H_16_O_2_]^−^	-	Organic acids and their derivatives
38	9,10,13-Trihydroxy-11-octadecenoic acid	C_18_H_34_O_5_	5.77	330.24	[M−H]^−^	329.23	−1.65	311.22 [M−H−H_2_O]^−^ 229.14 [M−H−C_6_H_12_O]^−^	29907-57-1	Organic acids and their derivatives
39	Cyclocurcumin	C_21_H_20_O_6_	6.16	368.13	[M+H]^+^	369.13	−0.12	351.12 [M+H−H_2_O]^+^ 337.11 [M+H−CH_4_O]^+^ 203.07 [M+H−C_9_H_10_O_3_]^+^ 167.07 [M+H−C_12_H_10_O_3_]^+^	153127-42-5	Others
40	Piperenol A	C_21_H_20_O_7_	6.25	384.12	[M+H]^+^	385.13	0.38	367.12 [M+H−H_2_O]^+^ 323.09 [M+H−C_2_H_6_O_2_]^+^ 177.05 [M+H−C_11_H_12_O_4_]^+^	134476-89-4	Lipids and their derivatives
41	Dehydrophytosphingosine	C_18_H_37_NO_3_	7.18	315.28	[M+H]^+^	316.28	−0.18	298.27 [M+H−H_2_O]^+^ 280.26 [M+H−2H_2_O]^+^	3687-54-5	Organonitrogen compounds and their derivatives
42	(5R)-1-(3,4-Dimethoxyphenyl)-5-hydroxydecan-3-one	C_18_H_28_O_4_	7.54	308.20	[M−H]^−^	307.19	−1.17	193.09 [M−H−C_7_H_14_O]^−^ 139.11 [M−H−C_9_H_12_O_3_]^−^	-	Others
43	1,2-Dioctanoyl-sn-glycerol	C_19_H_36_O_5_	7.56	344.26	[M−H]^−^	343.25	−1.35	201.11 [M−H−C_9_H_18_O]^−^ 157.09 [M−H−C_11_H_22_O_2_]^−^	60514-48-9	Lipids and their derivatives
44	Nigranoic acid	C_30_H_46_O_4_	8.12	470.34	[M−H]^−^	469.33	2.38	263.20 [M−H−C_13_H_18_O_2_]^−^	39111-07-4	Organic acids and their derivatives
45	6-Methylgingediol	C_18_H_30_O_4_	8.14	310.21	[M+Na]^+^	333.20	−2.13	293.21 [M+H−H_2_O]^+^ 121.06 [M+H−C_10_H_22_O_3_]^+^	-	Others
46	9-Hydroperoxy-10,12,15-octadecatrienoic acid	C_18_H_30_O_4_	8.22	310.21	[M+Na]^+^	333.20	−3.82	293.21 [M+H−H_2_O]^+^ 109.10 [M+H−C_10_H_18_O_4_]^+^ 95.09 [M+H−C_11_H_20_O_4_]^+^	111004-08-1	Organic acids and their derivatives
47	1-Monolinolein *	C_21_H_38_O_4_	8.58	354.28	[M+H]^+^	355.28	1.31	337.27 [M+H−H_2_O]^+^ 311.26 [M+H−C_2_H_4_O]^+^ 263.24 [M+H−C_3_H_8_O_3_]^+^	2277-28-3	Lipids and their derivatives
48	Gingerglycolipid B	C_33_H_58_O_14_	8.58	678.38	[M+COOH]^−^	723.38	1.14	415.15 [M−H−C_18_H_30_O]^−^ 397.14 [M−H−C_18_H_32_O_2_]^−^	88168-90-5	Carbohydrates and their derivatives
49	9R-Hydroxy-10E,12Z-octadecadienoic acid	C_18_H_32_O_3_	8.72	296.24	[M−H]^−^	295.23	−0.31	277.22 [M−H−H_2_O]^−^ 259.21 [M−H−2H_2_O]^−^ 249.22 [M−H−CH_2_O_2_]^−^ 195.14 [M−H−C_6_H_12_O]^−^ 171.10 [M−H−C_9_H_16_]^−^	10075-11-3	Organic acids and their derivatives
50	9-oxooctadeca-10,12-dienoic acid	C_18_H_30_O_3_	8.99	294.22	[M−H]^−^	293.21	−2.68	249.22 [M−H−CO_2_]^−^ 185.12 [M−H−C_8_H_12_]^−^	54232-58-5	Organic acids and their derivatives
51	Dibutyl phthalate	C_16_H_22_O_4_	9.01	278.15	[M+Na]^+^	301.14	1.05	149.02 [M+H−C_8_H_18_O]^+^ 79.05 [M+H−C_10_H_16_O_4_]^+^	84-74-2	Lipids and their derivatives
52	Vitetrifolin E	C_22_H_36_O_4_	9.05	364.26	[M+H]^+^	365.27	0.45	347.26 [M+H−H_2_O]^+^	-	Others
53	Mudanpinoic acid A	C_30_H_46_O_3_	9.26	454.34	[M+H]^+^	455.35	0.01	409.35 [M+H−CH_2_O_2_]^+^	203511-36-8	Steroids and their derivatives
54	1-Palmitoyl-galactosylglycerol	C_25_H_48_O_9_	9.45	492.33	[M+Na]^+^	515.32	−6.55	313.27 [M+H−C_6_H_12_O_6_]^+^	-	Lipids and their derivatives
55	12-Hydroxypentanoic acid methyl ester	C_15_H_30_O_3_	9.74	258.22	[M−H]^−^	257.21	−1.45	211.21 [M−H−CH_2_O_2_]^−^	-	Lipids and their derivatives
56	Bis (2-propylpentyl) phthalate	C_24_H_38_O_4_	10.00	390.28	[M+Na]^+^	413.27	1.12	149.02 [M+H−C_16_H_34_O]^+^	70910-37-1	Others
57	Brassicasterol	C_28_H_46_O	10.64	398.35	[M+H]^+^	399.36	−0.38	179.14 [M+H−C_16_H_28_]^+^ 109.10 [M+H−C_20_H_34_O]^+^ 81.07 [M+H−C_22_H_38_O]^+^ 67.05 [M+H−C_23_H_40_O]^+^	474-67-9	Steroids and their derivatives
58	Linoleic acid	C_18_H_32_O_2_	10.64	280.24	[M−H]^−^	279.23	−2.19	205.16 [M−H−C_4_H_10_O]^−^ 163.11 [M−H−C_7_H_16_O]^−^ 113.06 [M−H−C_12_H_22_]^−^ 83.05 [M−H−C_13_H_24_O]^−^	60-33-3	Organic acids and their derivatives
59	9(Z)-Octadecenamide	C_18_H_35_NO	10.77	281.27	[M+H]^+^	282.28	4.62	123.12 [M+H−C_9_H_21_NO]^+^ 109.10 [M+H−C_10_H_23_NO]^+^ 81.07 [M+H−C_12_H_27_NO]^+^	301-02-0	Organonitrogen compounds and their derivatives

The symbol ‘*’ indicates compounds were identified using standard substances, the remaining compounds were identified by fragmentation regularities.

**Table 4:** The detailed information of differential compounds between *Laportea bulbifera* and *Girardinia diversifolia.*

NO.	Compound	VIP Value	*p*	Class
1	Baimaside	16.9932	9.23 × 10^−4^	Flavonoids and their derivatives
2	Gallocatechin-(4alpha->8)-epicatechin	8.8361	2.72 × 10^−6^	Flavonoids and their derivatives
3	Protocatechuic acid 3-glucoside	6.5556	1.61 × 10^−2^	Carbohydrates and their derivatives
4	Catechin	5.4767	1.62 × 10^−3^	Flavonoids and their derivatives
5	Roxburic acid	5.0877	3.33 × 10^−7^	Terpenoids and their derivatives
6	Procyanidin B1	4.7645	7.01 × 10^−3^	Flavonoids and their derivatives
7	Schaftoside	4.3478	3.04 × 10^−6^	Flavonoids and their derivatives
8	Cuneataside E	4.2182	9.60 × 10^−3^	Terpenoids and their derivatives
9	Quercetin-3-*O*-beta-D-glucose-7-*O*-beta-D-gentiobioside	4.2061	1.56 × 10^−2^	Flavonoids and their derivatives
10	Phenylalanine	3.6882	2.96 × 10^−7^	Organonitrogen compounds and their derivatives
11	9,12,13,TriHODE	2.1420	2.59 × 10^−2^	Organic acids and their derivatives
12	Luteolin	2.1411	3.62 × 10^−4^	Flavonoids and their derivatives
13	Malic acid	1.9732	3.52 × 10^−2^	Organic acids and their derivatives
14	Interiotherin A	1.8982	6.14 × 10^−4^	Others
15	7,8-Dehydropenstemoside	1.8620	4.74 × 10^−3^	Terpenoids and their derivatives
16	Linoleic acid	1.7444	2.81 × 10^−3^	Organic acids and their derivatives
17	1-Galloyl-glucose	1.7205	8.42 × 10^−5^	Carbohydrates and their derivatives
18	Heptyl 6-*O*-α-L -arabinopyranosyl-β-D-glucopyranoside	1.6129	9.31 × 10^−4^	Carbohydrates and their derivatives
19	Esculentoside B	1.6104	9.84 × 10^−4^	Terpenoids and their derivatives
20	2-*O*-alpha-D-Glucopyranosyl-L-ascorbic acid	1.6006	1.95 × 10^−8^	Carbohydrates and their derivatives
21	Pantothenic acid	1.5922	1.42 × 10^−2^	Organonitrogen compounds and their derivatives
22	4-*O*-Feruloylquinic acid	1.5555	3.16 × 10^−5^	Organic acids and their derivatives
23	Eriodictyol 7-*O*-glucoside	1.5367	8.36 × 10^−5^	Flavonoids and their derivatives
24	(+)-Lariciresinol 4′-glucoside	1.4578	1.05 × 10^−3^	Carbohydrates and their derivatives
25	Benzoic acid	1.3395	4.28 × 10^−6^	Organic acids and their derivatives
26	6-Hydroxyluteolin 7-glucoside	1.3256	3.17 × 10^−3^	Flavonoids and their derivatives
27	Ethyl gallate	1.1449	2.65 × 10^−5^	Lipids and their derivatives
28	5alpha-Hydroxytriptonide	1.0931	3.29 × 10^−6^	Others
29	Epiafzelechin 5-*O*-beta-D-glucopyranoside	1.0766	1.09 × 10^−4^	Flavonoids and their derivatives
30	(−)-Epicatechin-3-*O*-gallate	1.0765	1.73 × 10^−4^	Flavonoids and their derivatives
31	5-Hydroxy-7,4′-dimethoxyflavone	1.0375	4.82 × 10^−4^	Flavonoids and their derivatives
32	Isochlorogenic acid A	1.0161	3.73 × 10^−4^	Organic acids and their derivatives

**Table 5:** The detailed information of differential compounds between *Laportea bulbifera* and *Urtica mairei*.

NO.	Compound	VIP Value	*p*	Class
1	Baimaside	16.7991	9.19 × 10^−4^	Flavonoids and their derivatives
2	Gallocatechin-(4alpha->8)-epicatechin	8.7260	2.75 × 10^−6^	Flavonoids and their derivatives
3	Catechin	5.4357	1.55 × 10^−3^	Flavonoids and their derivatives
4	Procyanidin B1	4.7163	6.92 × 10^−3^	Flavonoids and their derivatives
5	Cuneataside E	4.1574	9.79 × 10^−3^	Terpenoids and their derivatives
6	Quercetin-3-*O*-beta-D-glucose-7-*O*-beta-D-gentiobioside	4.1566	1.56 × 10^−2^	Flavonoids and their derivatives
7	Phenylalanine	3.8482	1.70 × 10^−6^	Organonitrogen compounds and their derivatives
8	Kaempferol 7-*O*-rhamnoside	3.3749	1.80 × 10^−3^	Flavonoids and their derivatives
9	7,8-Dehydropenstemoside	1.9225	3.26 × 10^−3^	Terpenoids and their derivatives
10	1-Galloyl-glucose	1.8915	1.30 × 10^−5^	Carbohydrates and their derivatives
11	Linoleic acid	1.7689	1.46 × 10^−3^	Organic acids and their derivatives
12	Benzyl alcohol xylopyranosyl(1 → 6)glucopyranoside	1.7111	4.28 × 10^−3^	Carbohydrates and their derivatives
13	2-*O*-alpha-D-Glucopyranosyl-L-ascorbic acid	1.6226	8.95 × 10^−9^	Carbohydrates and their derivatives
14	Heptyl 6-*O*-α-L-arabinopyranosyl-β-D-glucopyranoside	1.5978	9.07 × 10^−4^	Carbohydrates and their derivatives
15	Eriodictyol 7-*O*-glucoside	1.5413	6.74 × 10^−5^	Flavonoids and their derivatives
16	4-*O*-Feruloylquinic acid	1.5083	4.22 × 10^−5^	Organic acids and their derivatives
17	(5R)-1-(3,4-Dimethoxyphenyl)-5-hydroxydecan-3-one	1.3820	1.01 × 10^−6^	Others
18	Isoeucommin A	1.3373	3.12 × 10^−3^	Carbohydrates and their derivatives
19	(+)-Lariciresinol 4′-glucoside	1.3324	2.47 × 10^−3^	Carbohydrates and their derivatives
20	3-Hexenyl-beta-glucopyranoside	1.1283	1.49 × 10^−3^	Carbohydrates and their derivatives
21	D-Galactono-1,4-lactone	1.1266	1.78 × 10^−2^	Carbohydrates and their derivatives
22	5-Hydroxy-7,4′-dimethoxyflavone	1.1153	1.39 × 10^−4^	Flavonoids and their derivatives
23	Epiafzelechin 5-*O*-beta-D-glucopyranoside	1.0670	1.05 × 10^−4^	Flavonoids and their derivatives
24	(−)-Epicatechin-3-*O*-gallate	1.0545	1.94 × 10^−4^	Flavonoids and their derivatives
25	Isochlorogenic acid A	1.0485	2.04 × 10^−4^	Organic acids and their derivatives
26	3,6-Dihydroxycoumarin	1.0131	8.58 × 10^−4^	Coumarins and their derivatives
27	Luteolin	1.0045	2.30 × 10^−3^	Flavonoids and their derivatives

**Table 6:** The detailed information of differential compounds between *Girardinia diversifolia* and *Urtica mairei.*

NO.	Compound	VIP Value	*p*	Class
1	Protocatechuic acid 3-glucoside	13.3082	4.29 × 10^−5^	Carbohydrates and their derivatives
2	Roxburic acid	5.7305	1.60 × 10^−6^	Terpenoids and their derivatives
3	Schaftoside	4.9349	1.98 × 10^−5^	Flavonoids and their derivatives
4	Bruceine C	4.3506	2.15 × 10^−4^	Terpenoids and their derivatives
5	Luteolin	2.9339	4.07 × 10^−5^	Flavonoids and their derivatives
6	Apigenin	2.35284	5.14 × 10^−4^	Flavonoids and their derivatives
7	Interiotherin A	2.2987	5.12 × 10^−4^	Others
8	Esculentoside B	1.9247	9.60 × 10^−4^	Terpenoids and their derivatives
9	Isoeucommin A	1.6750	2.09 × 10^−3^	Carbohydrates and their derivatives
10	(5R)-1-(3,4-Dimethoxyphenyl)-5-hydroxydecan-3-one	1.5031	5.06 × 10^−6^	Others
11	Dichotomoside A	1.2729	1.36 × 10^−2^	Organic acids and their derivatives
12	3-Hexenyl-beta-glucopyranoside	1.2639	3.00 × 10^−3^	Carbohydrates and their derivatives
13	L-Proline	1.1908	5.00 × 10^−3^	Organonitrogen compounds and their derivatives

## Data Availability

The original contributions presented in this study are included in the article/Supplementary Material. Further inquiries can be directed to the corresponding authors.

## Supplementary Materials

The following supporting information can be downloaded at: https://www.mdpi.com/article/10.3390/molecules31152614/s1. Figure S1: The MS/MS spectra of reference standards. Table S1: Experimental sample information; Table S2: The Standard compounds for experiments.
