# Supplementary material for: Chemical Profiling and Differential Constituents Analysis of Honghema and Its Easily Confusable Species Driven by High-Resolution Mass Spectrometry and Pattern Recognition
Source: Molecules. 2026 Jul 27;31(15):2614. doi: 10.3390/molecules31152614 (PMC13468369; doi:10.3390/molecules31152614)

Rutin

Rutin-POS

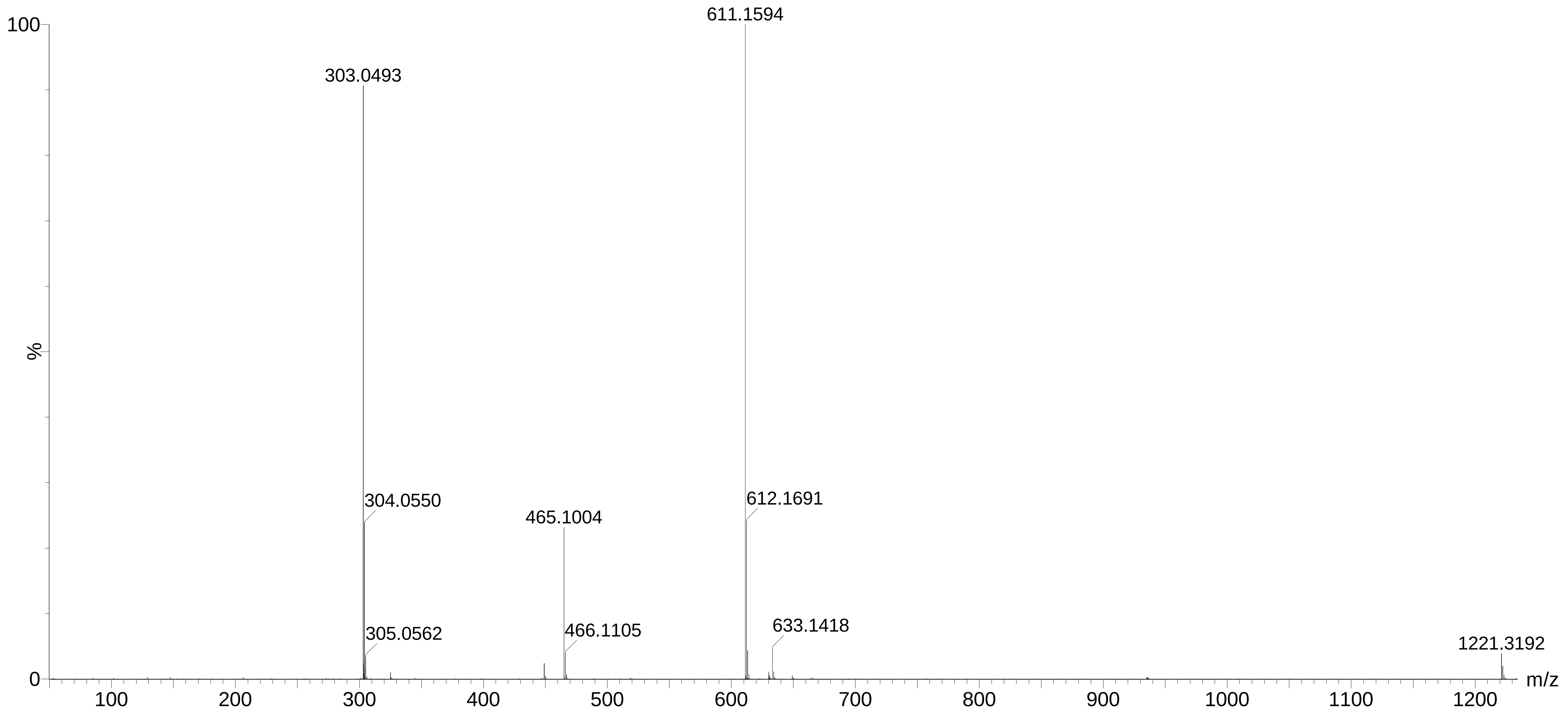

611.1594
303.0493
304.0550
465.1004
612.1691
305.0562
466.1105
633.1418
1221.3192
100
%
0
100
200
300
400
500
600
700
800
900
1000
1100
1200
m/z

Rutin-NEG

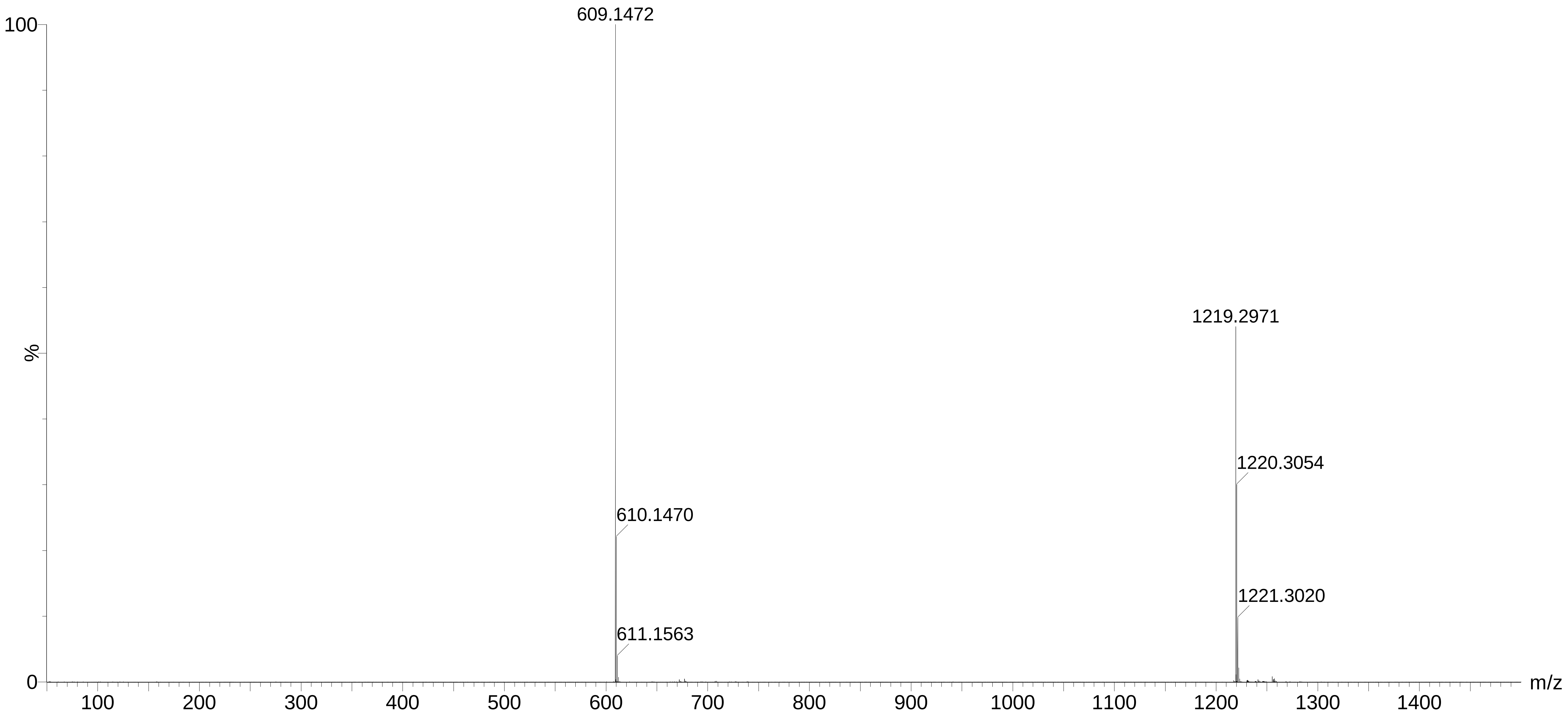

609.1472
100
%
0
610.1470
611.1563
1219.2971
1220.3054
1221.3020
m/z
100
200
300
400
500
600
700
800
900
1000
1100
1200
1300
1400

Luteolin

Luteolin-POS

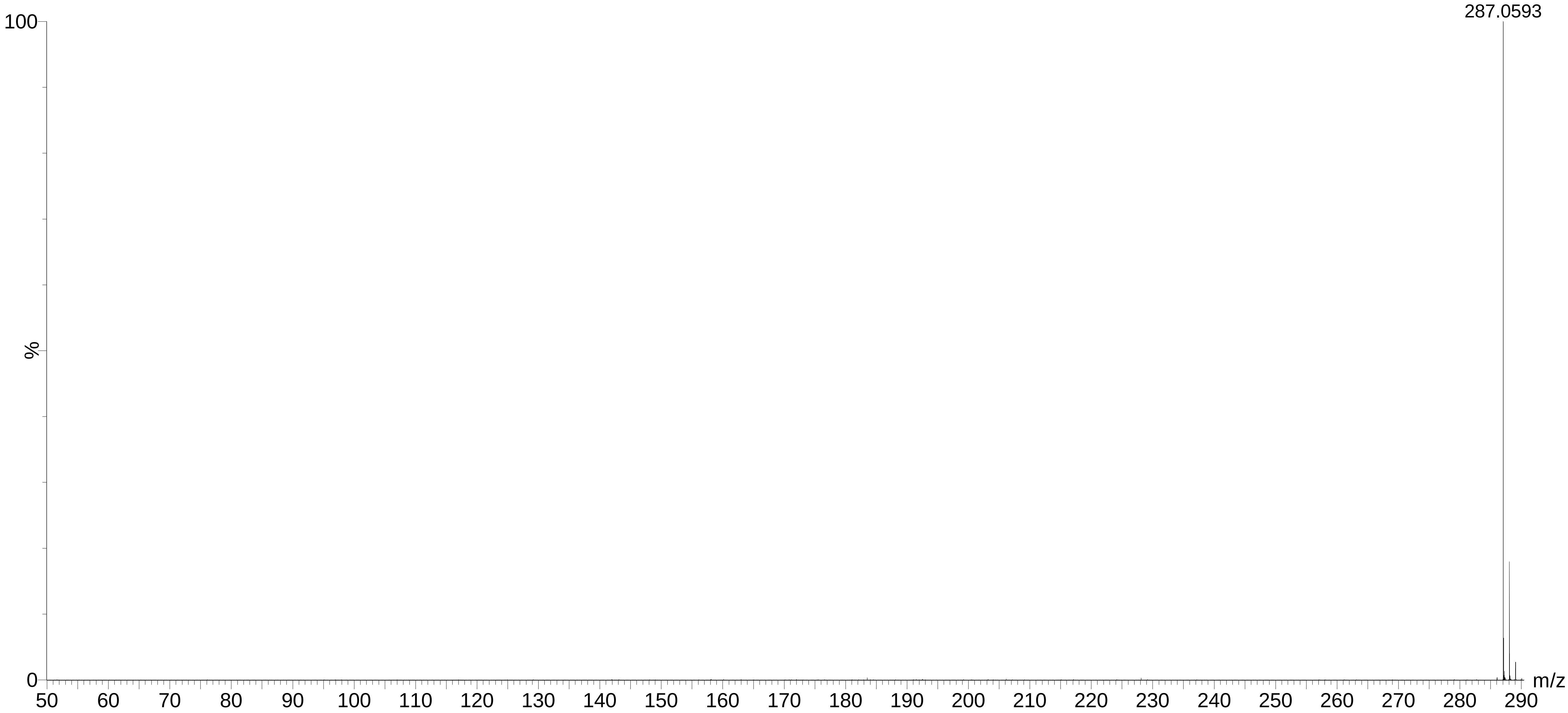

Luteolin-NEG

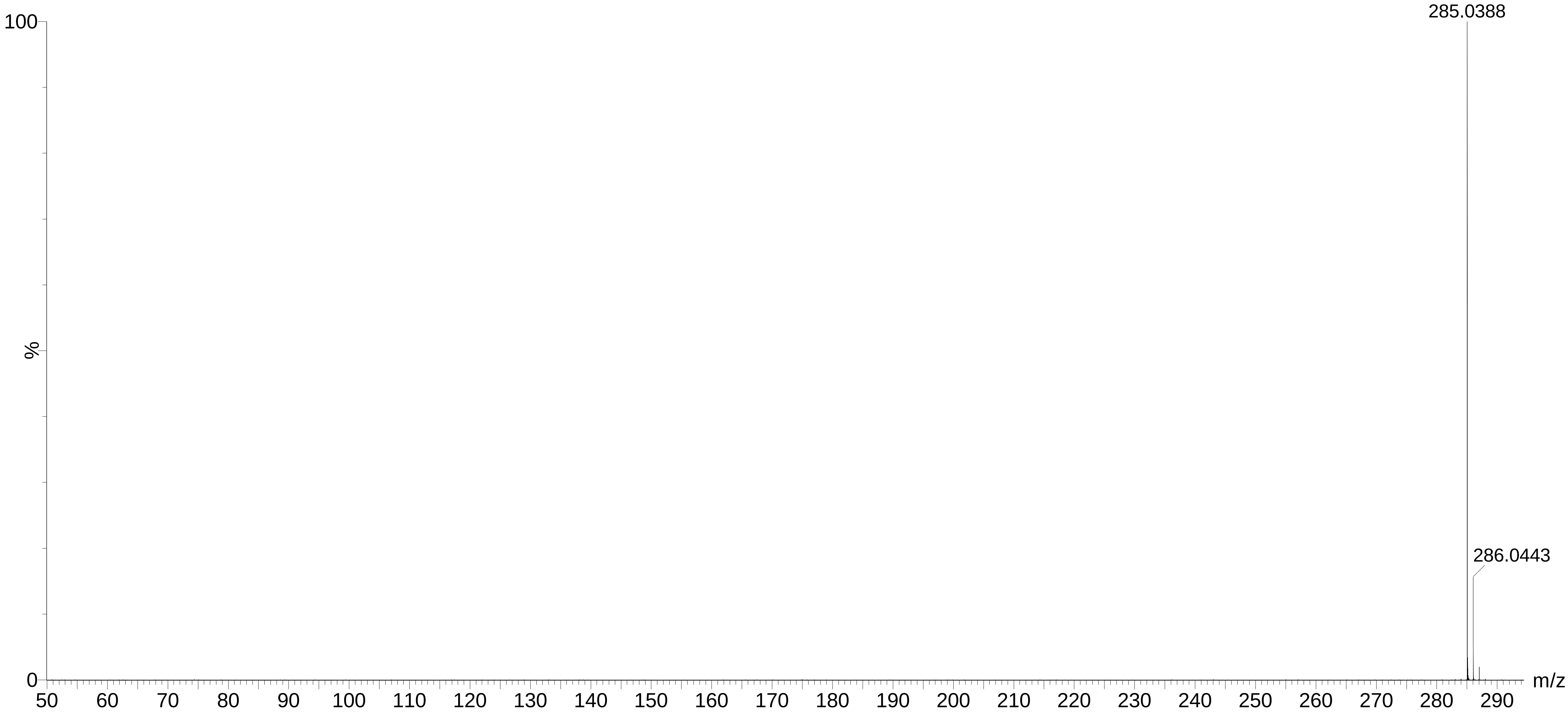

9-Ribofuranosyladenine

9-Ribofuranosyladenine-POS

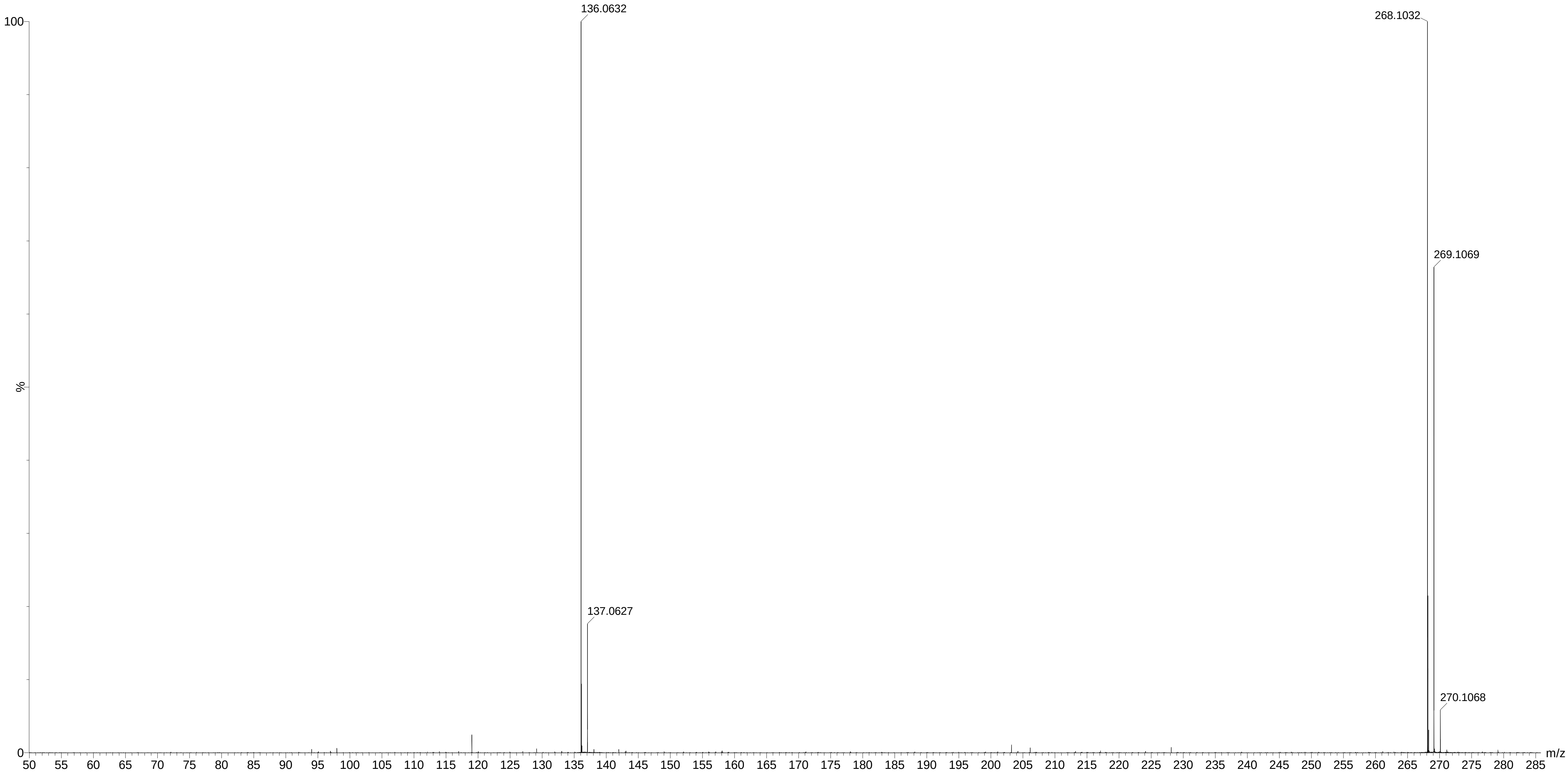

9-Ribofuranosyladenine-NEG

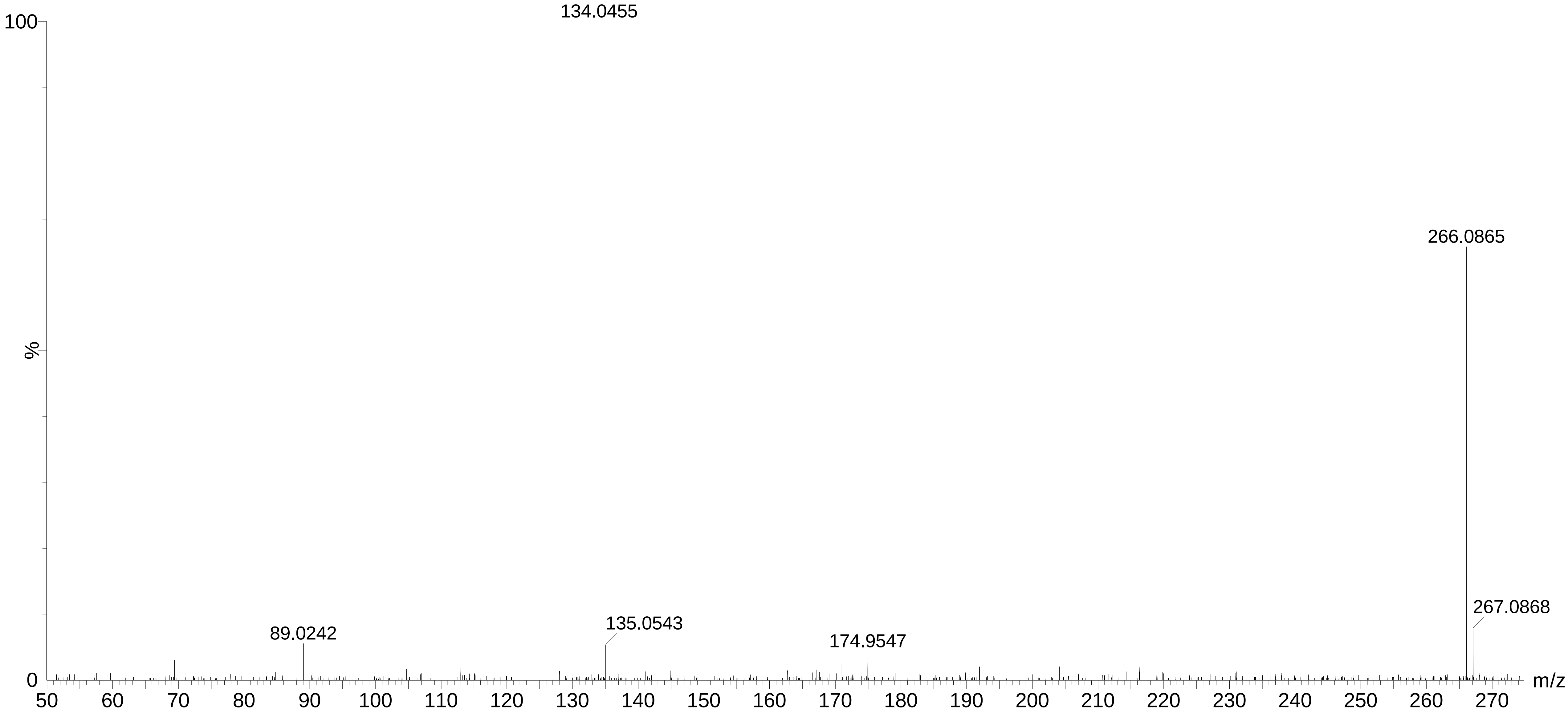

Quercetin-3-O-beta-D-glucose-7-O-beta-D-gentiobioside

Quercetin-3-O-beta-D-glucose-7-O-beta-D-gentiobioside-POS

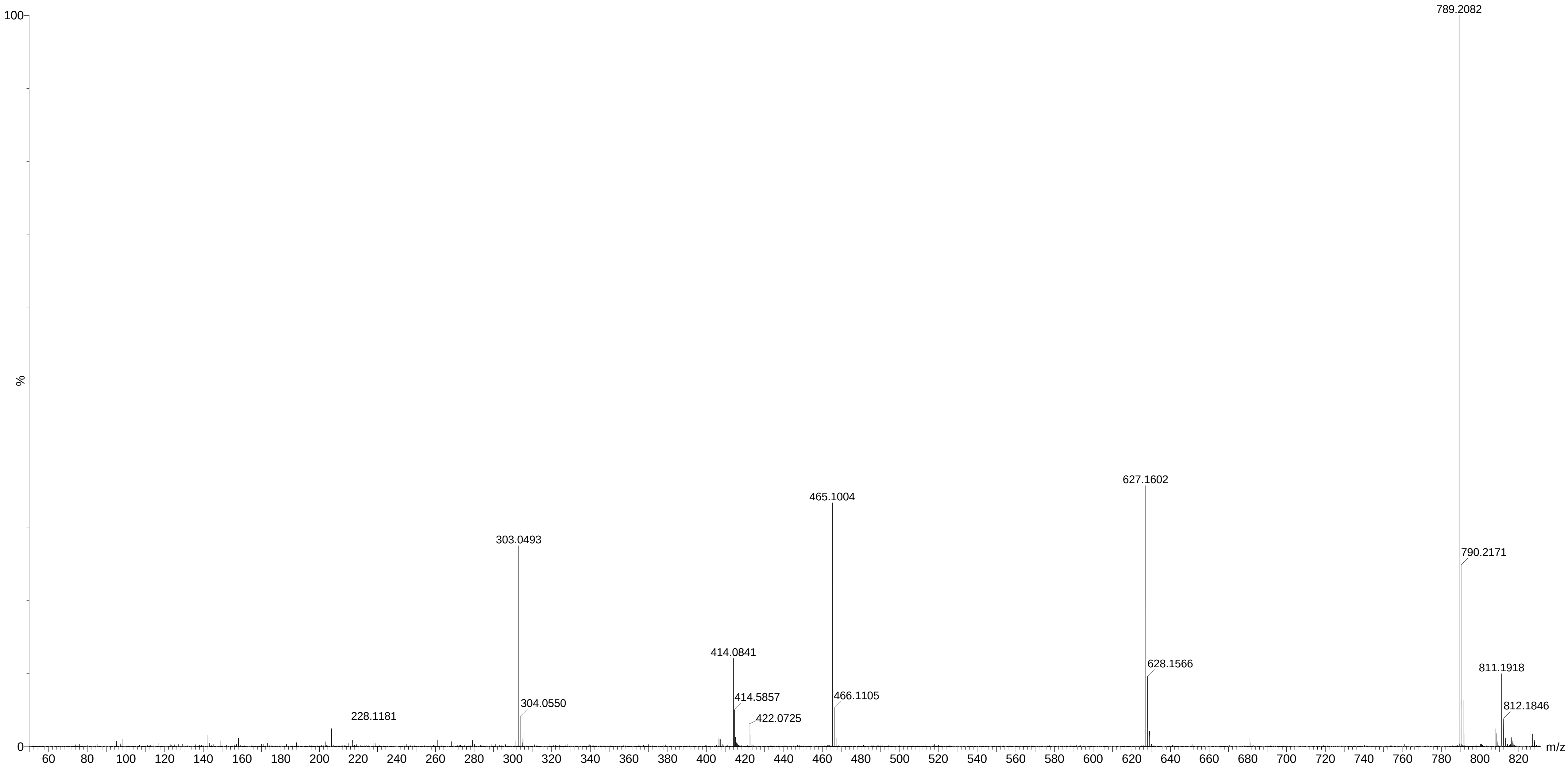

Quercetin-3-O-beta-D-glucose-7-O-beta-D-gentiobioside-NEG

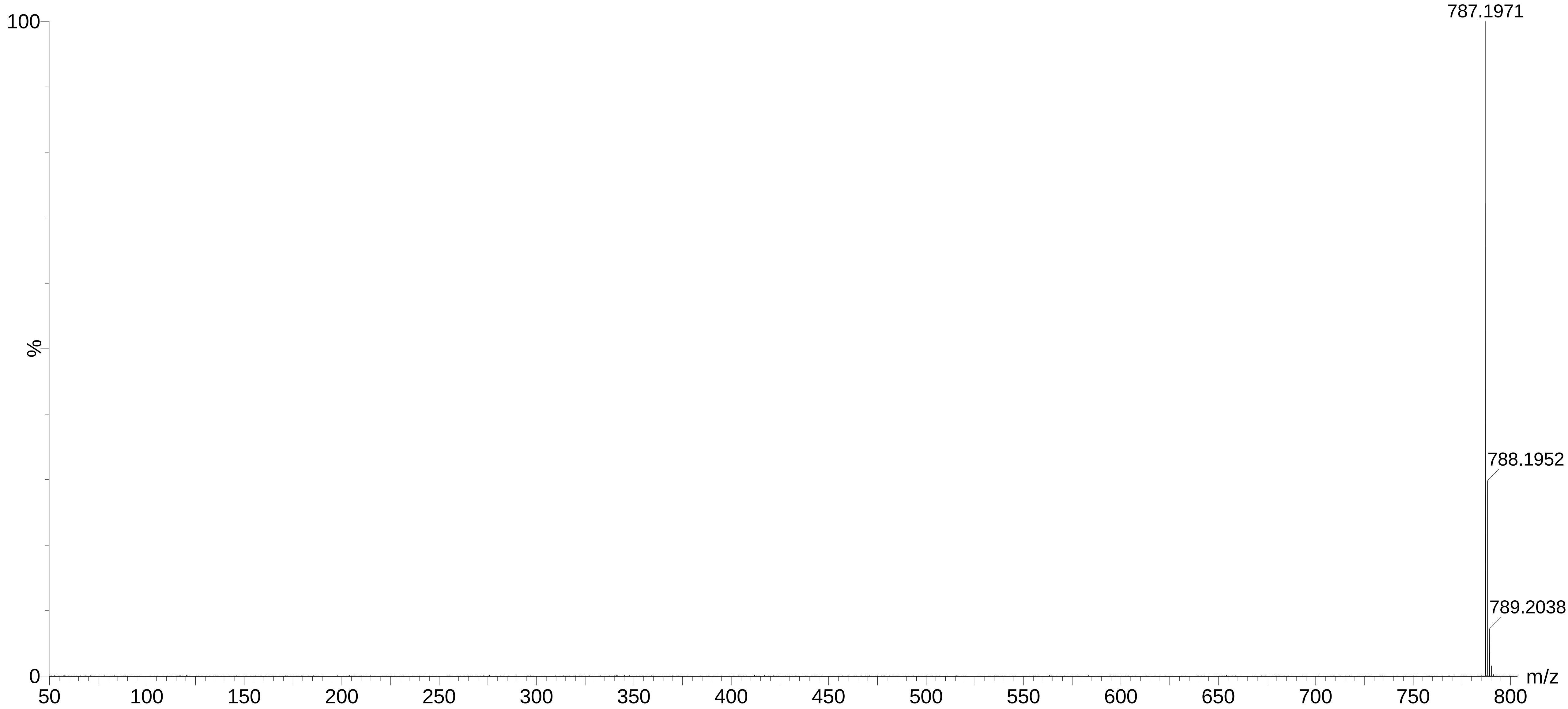

Chlorogenic acid

Chlorogenic acid -POS

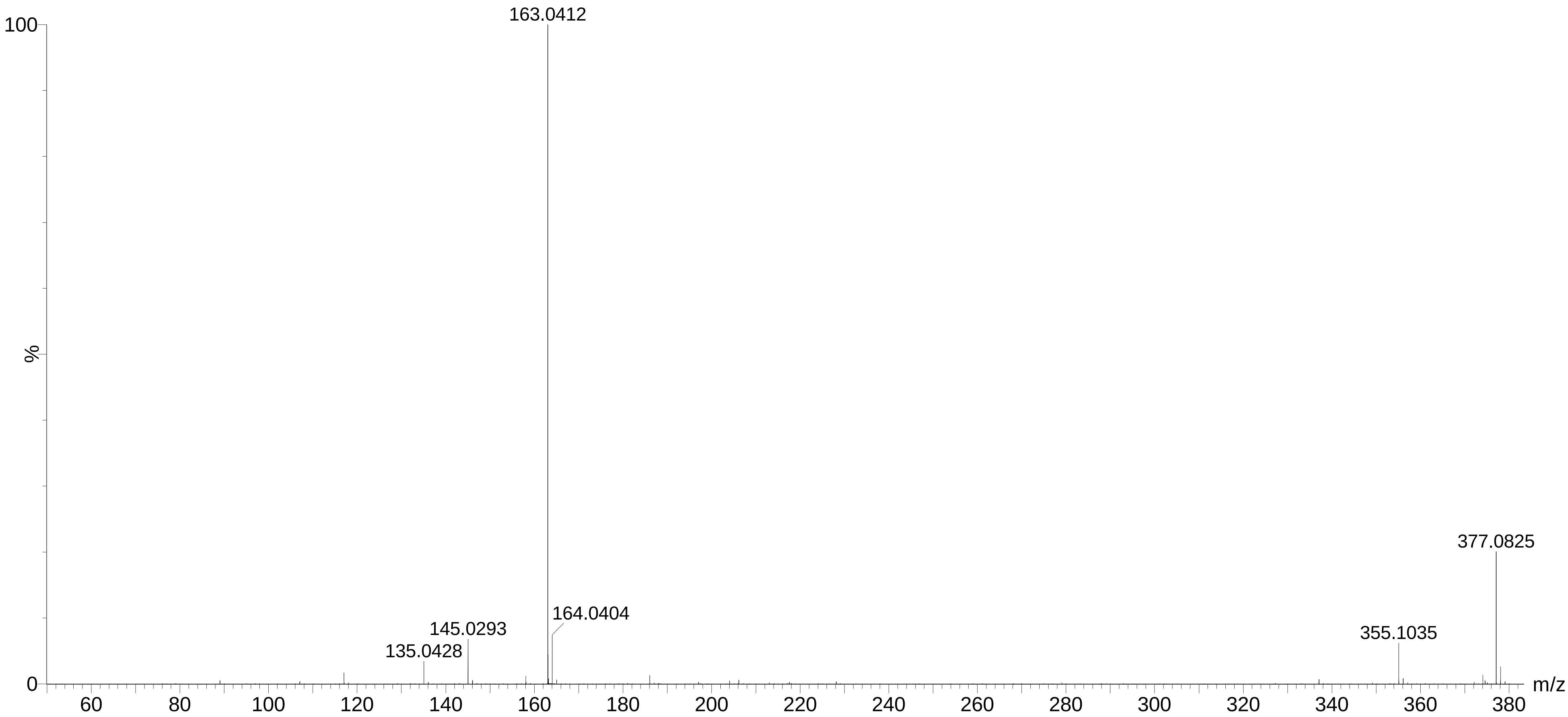

Chlorogenic acid -NEG

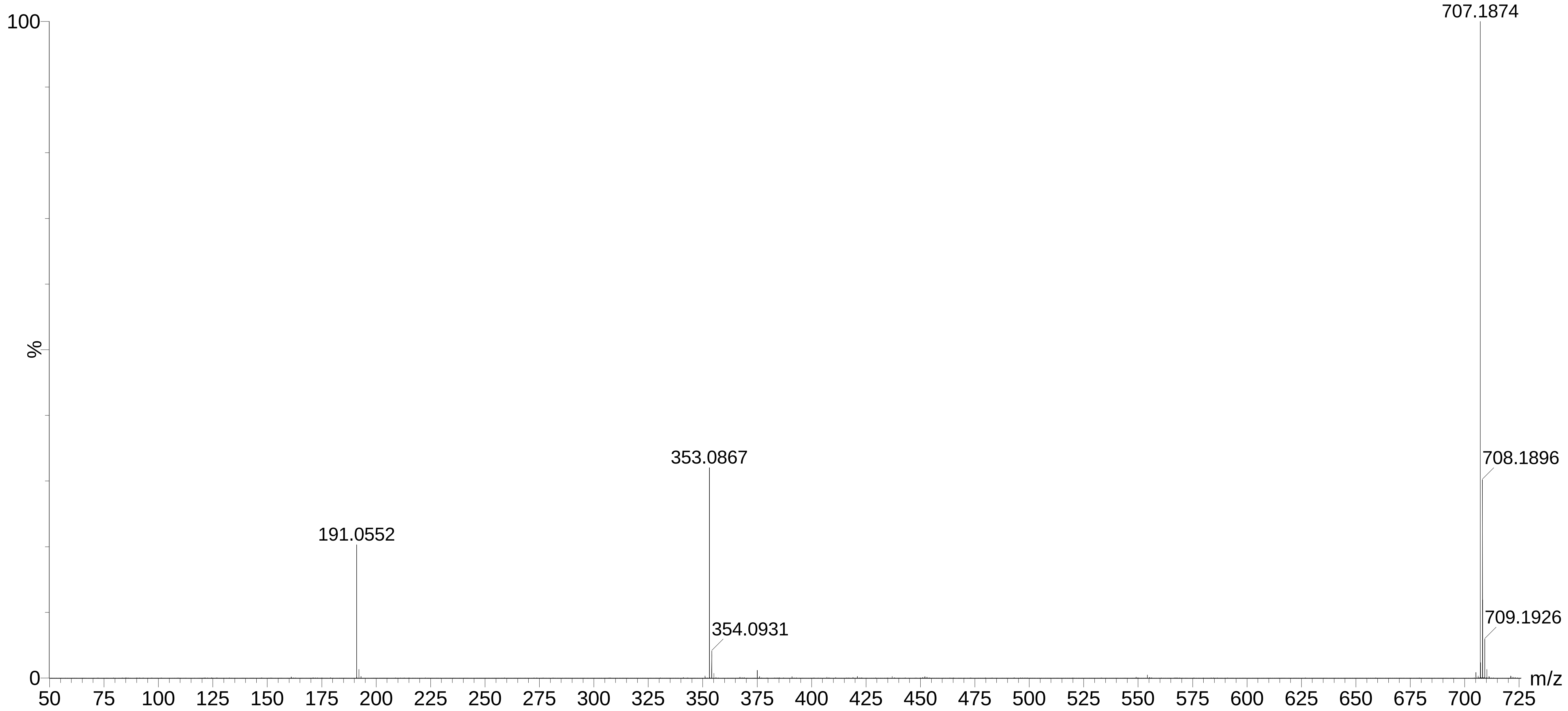

Kaempferol

Kaempferol-POS

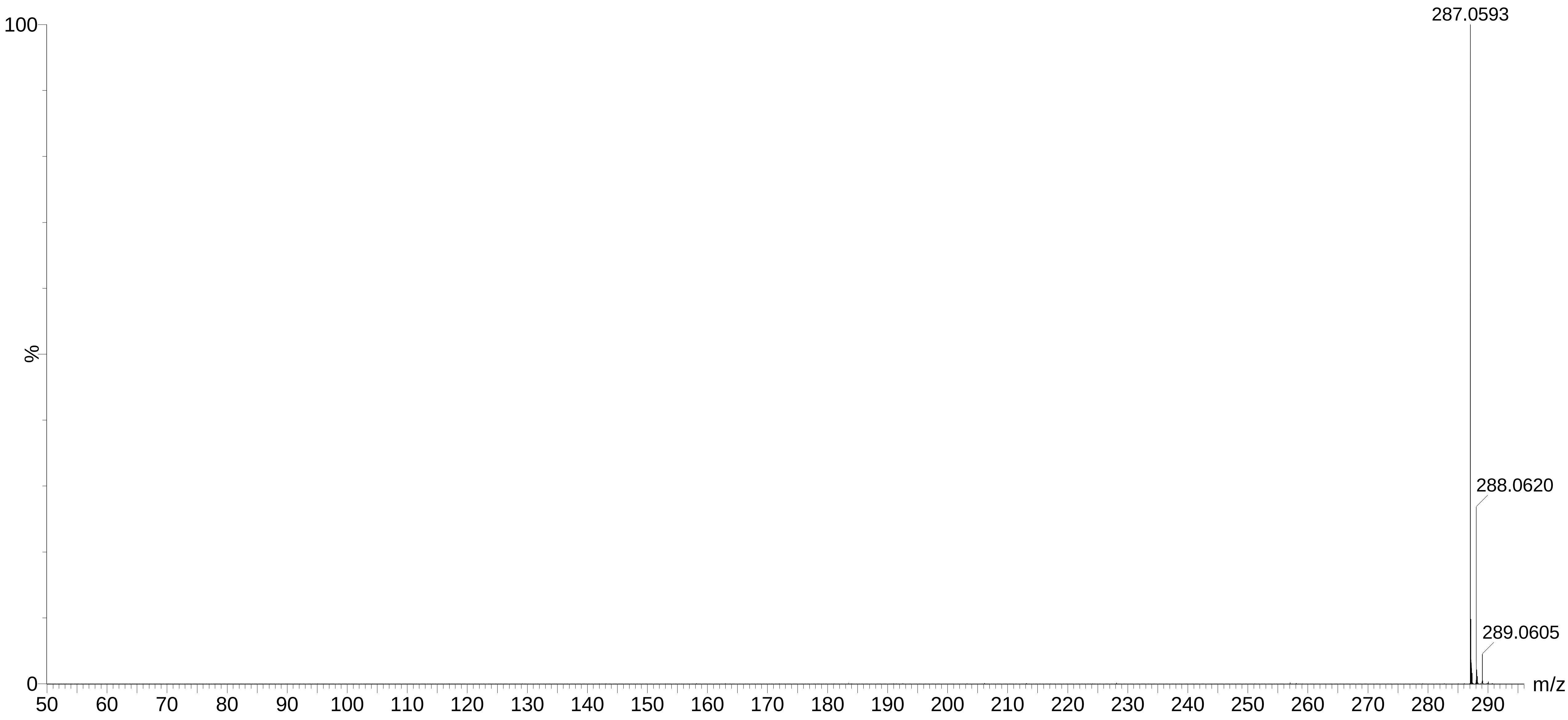

Kaempferol -NEG

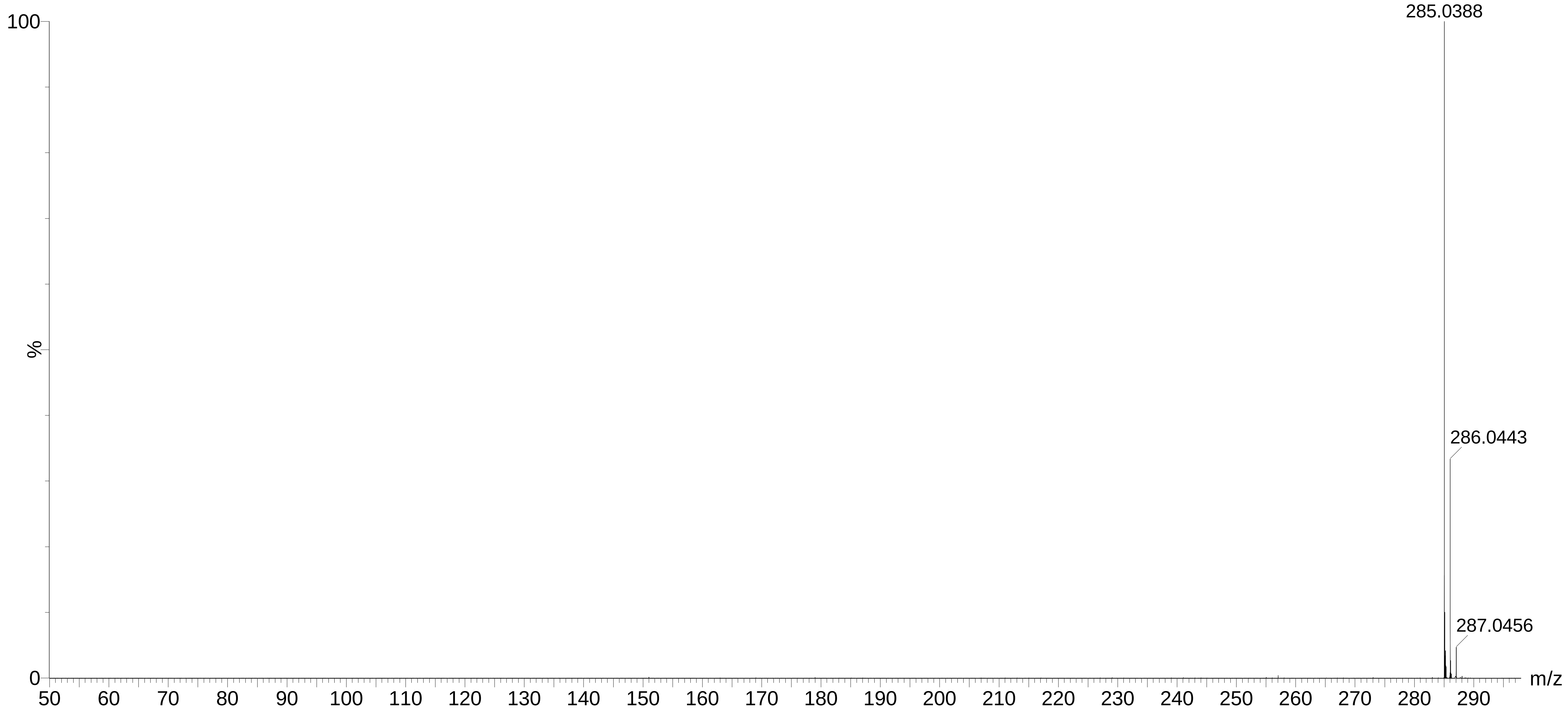

Citric acid

Citric acid-POS

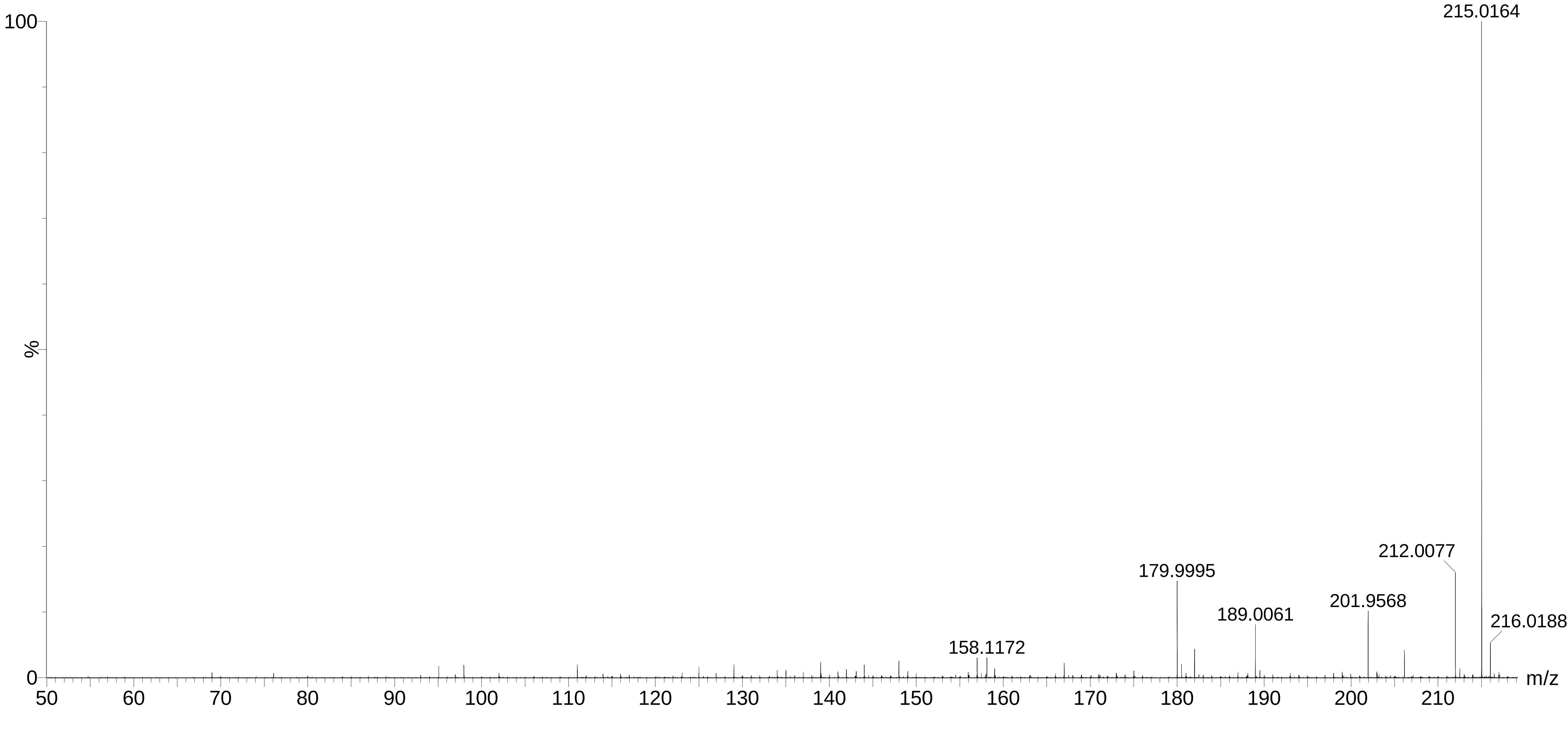

Citric acid-NEG

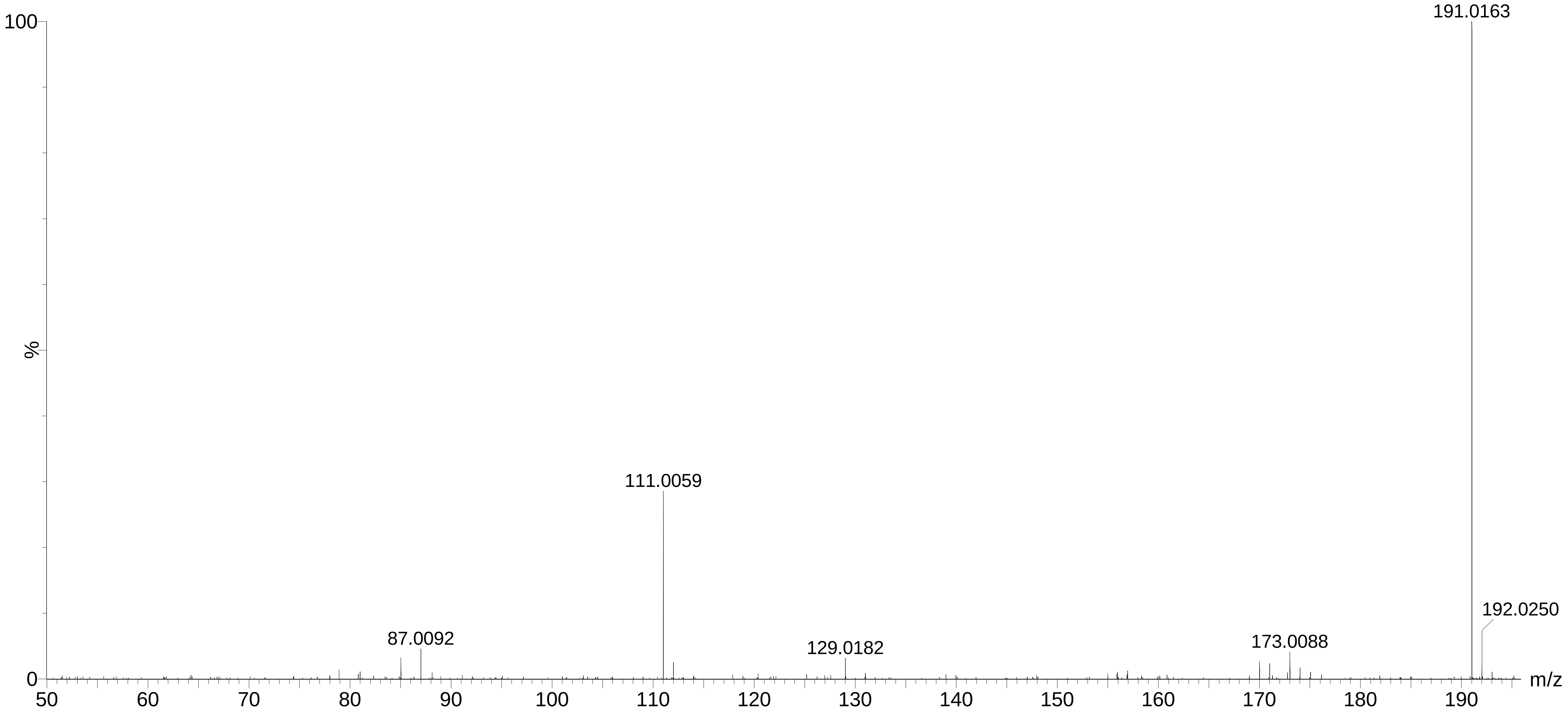

Cryptochlorogenic acid

Cryptochlorogenic acid-POS（1.97）

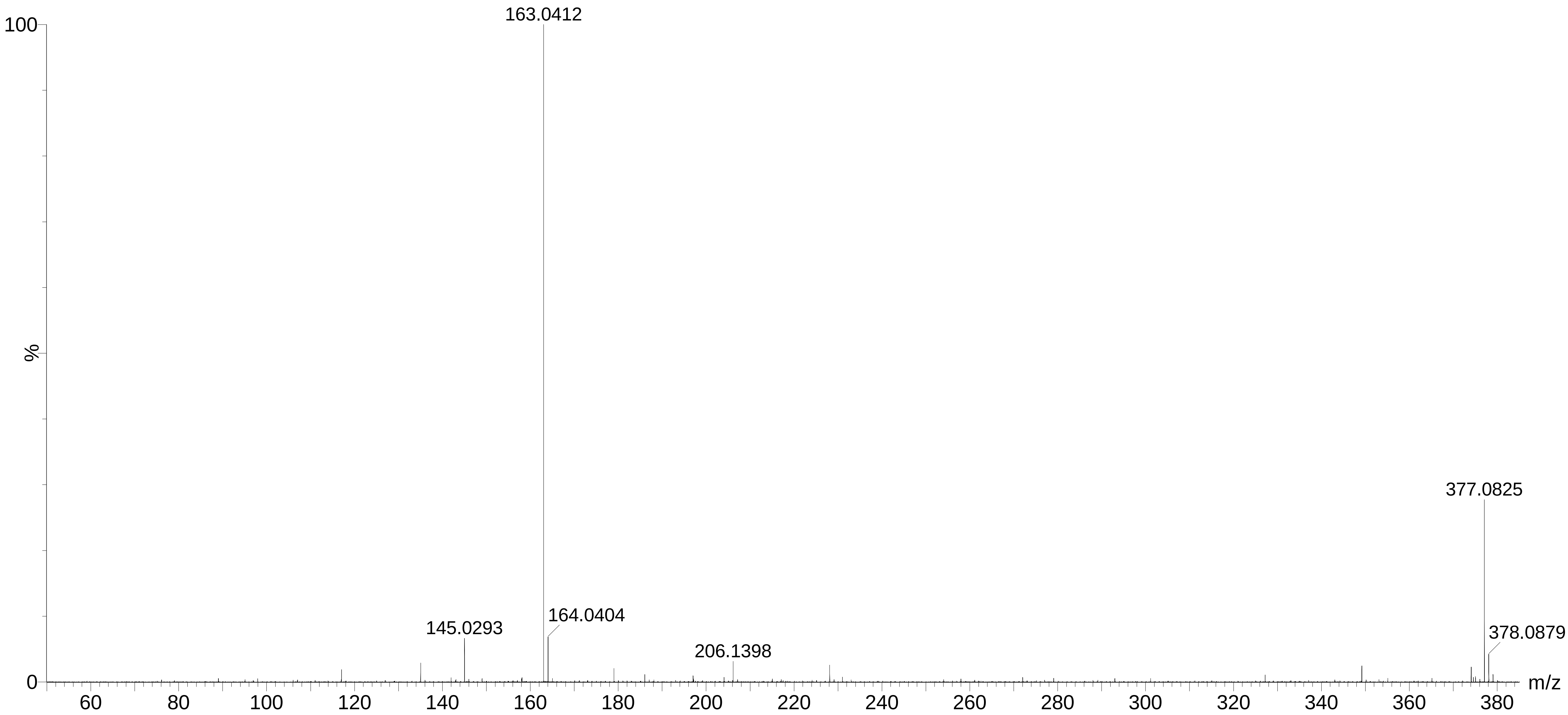

Cryptochlorogenic acid-NEG

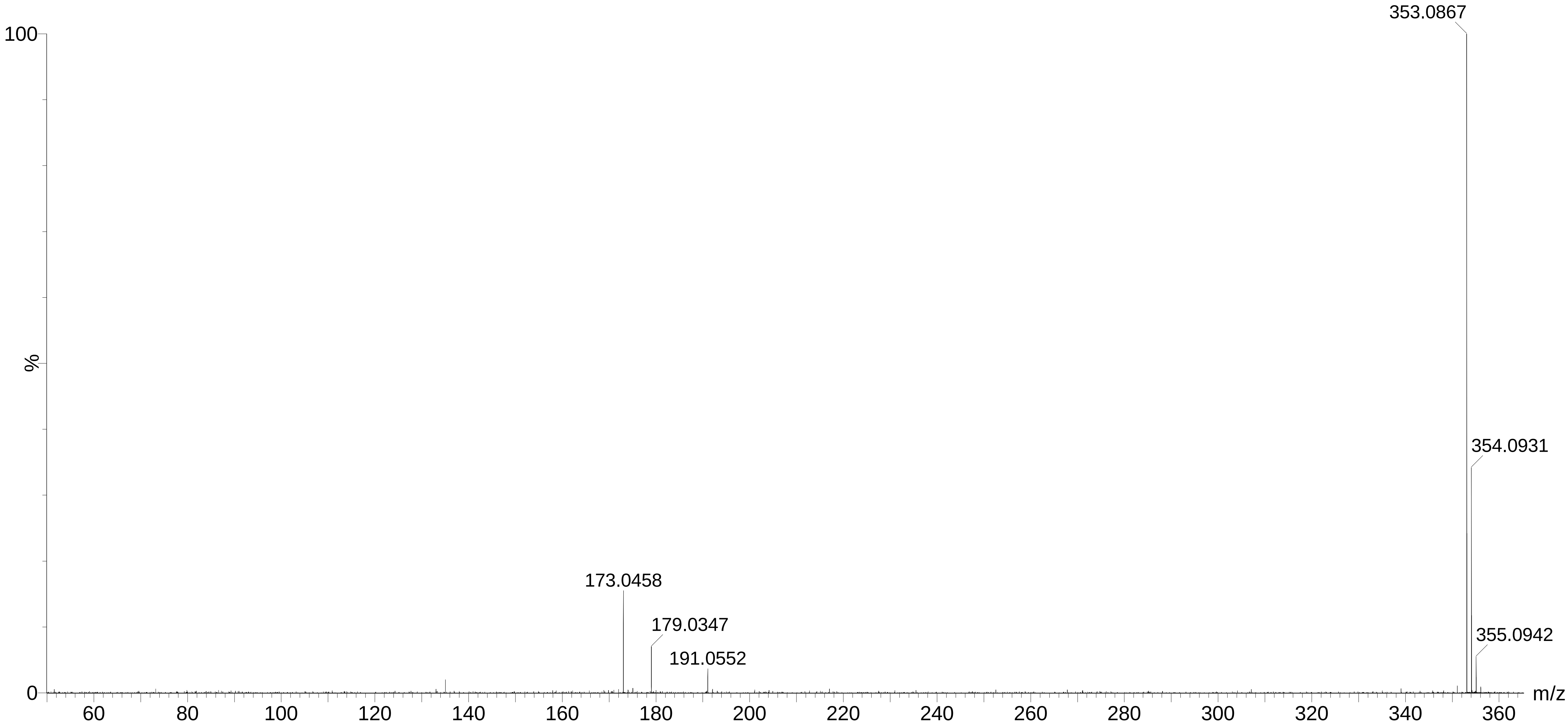

Ferulic acid

Ferulic acid-POS

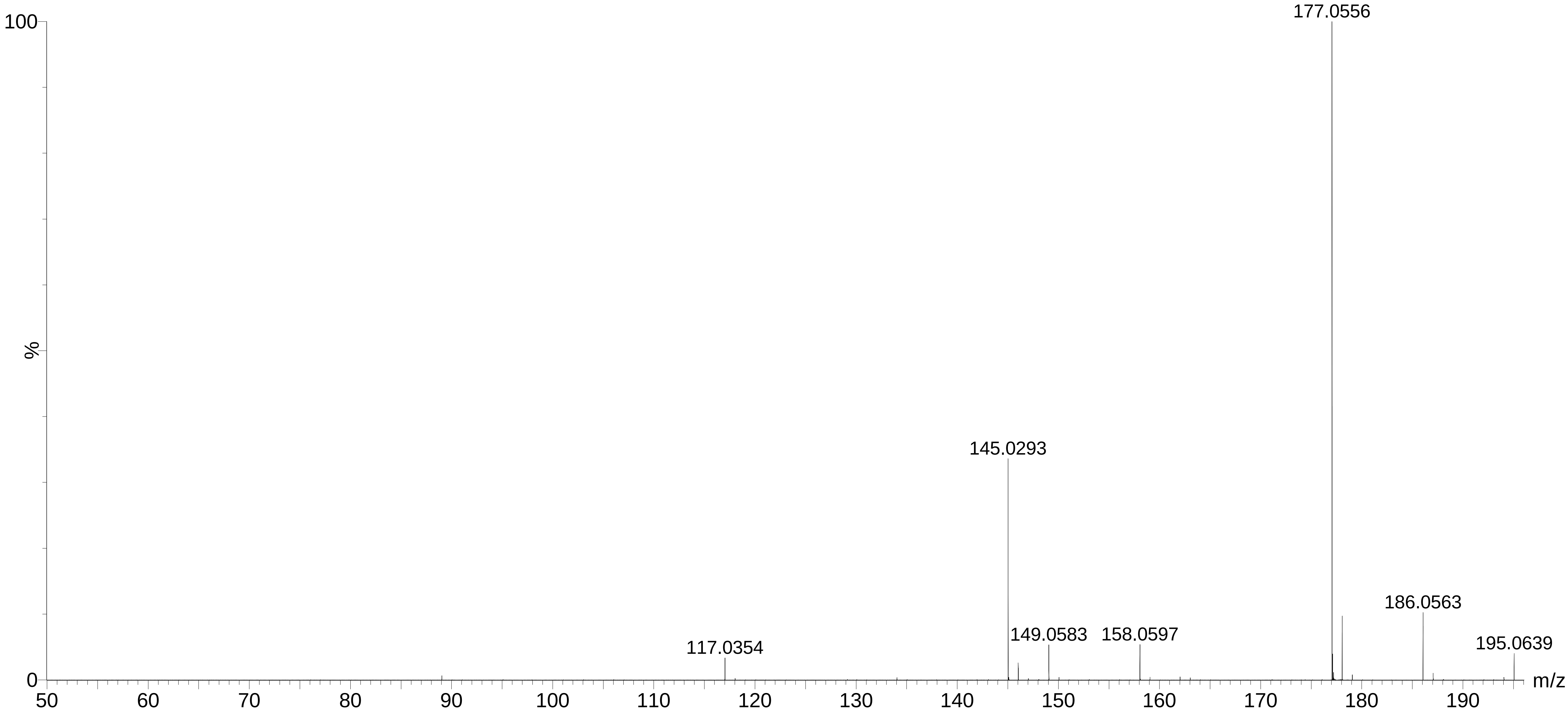

Ferulic acid-NEG

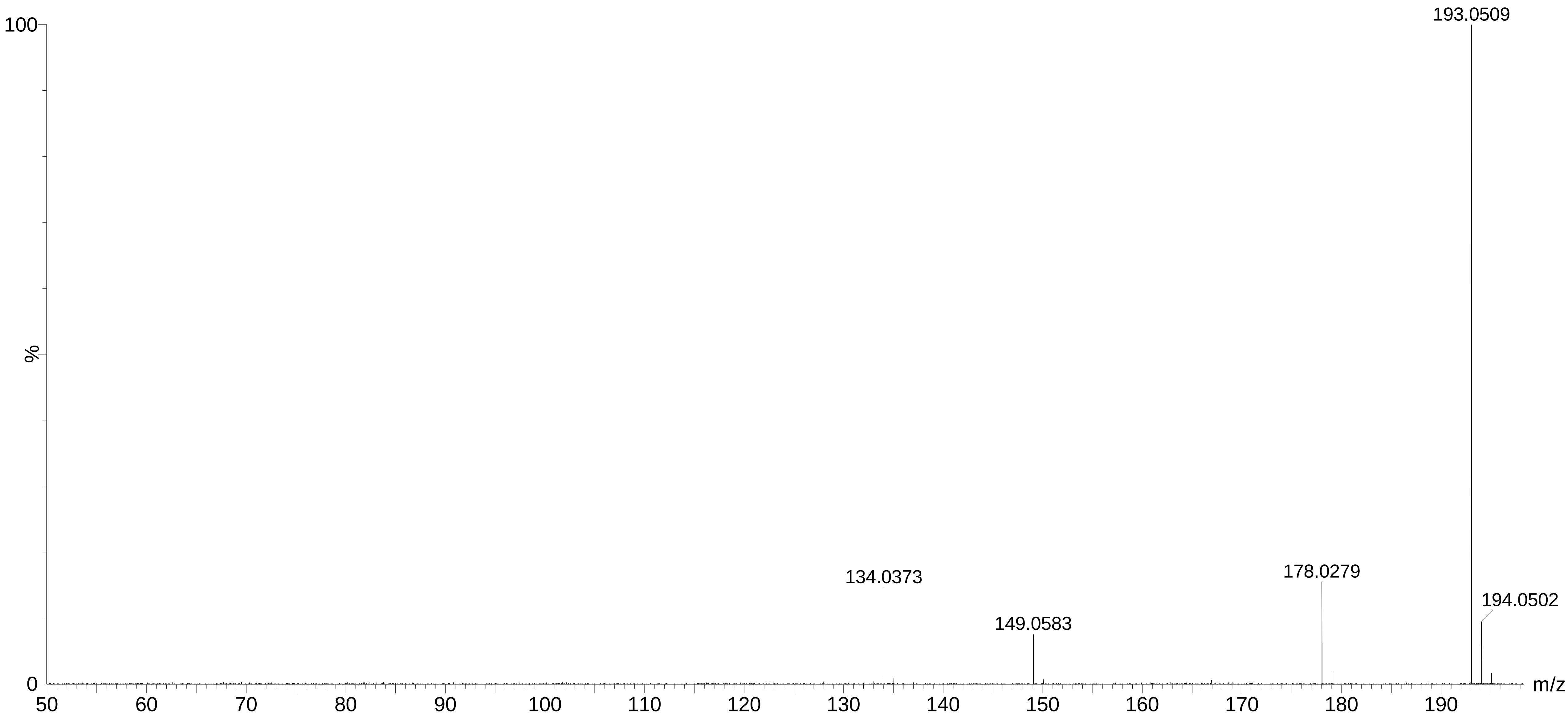

Tryptophan

Tryptophan-POS

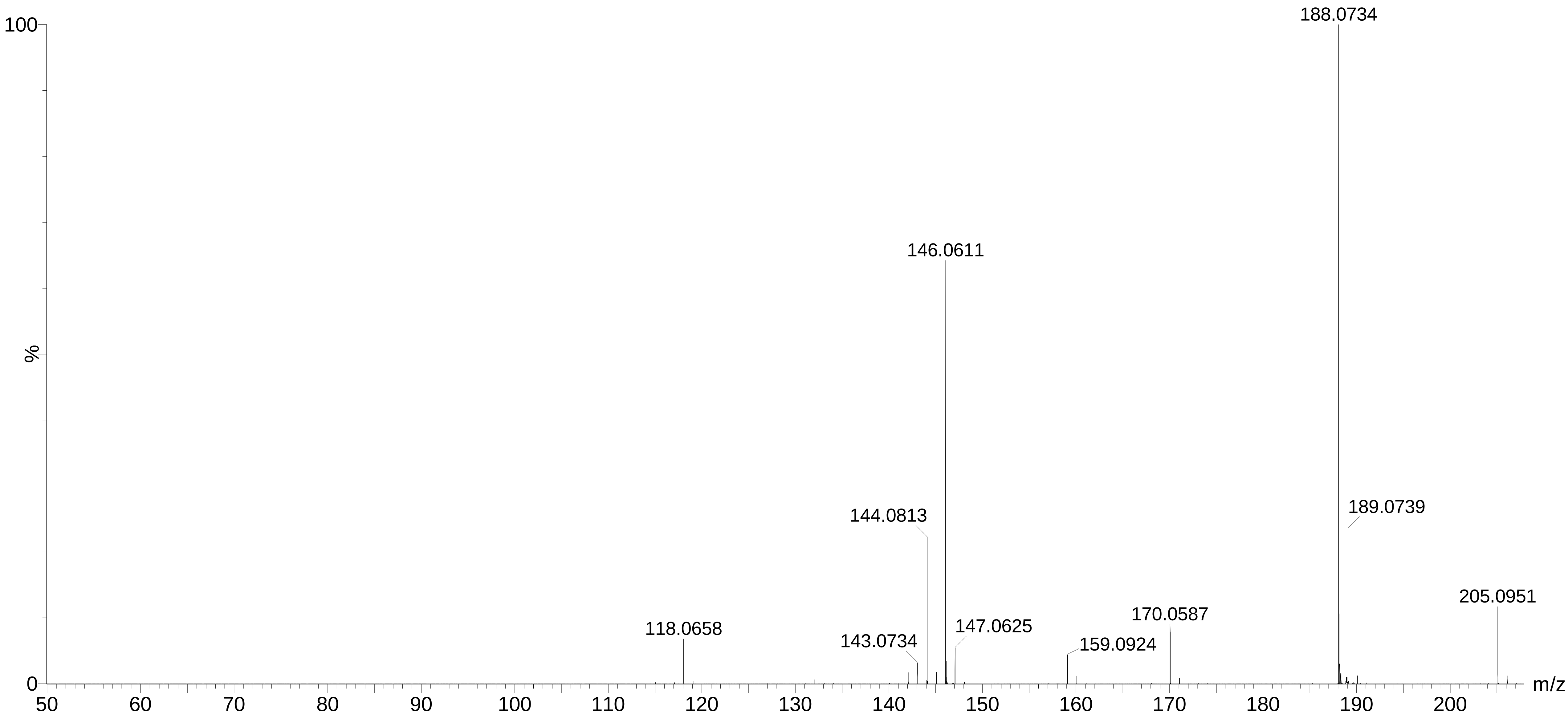

Tryptophan-NEG

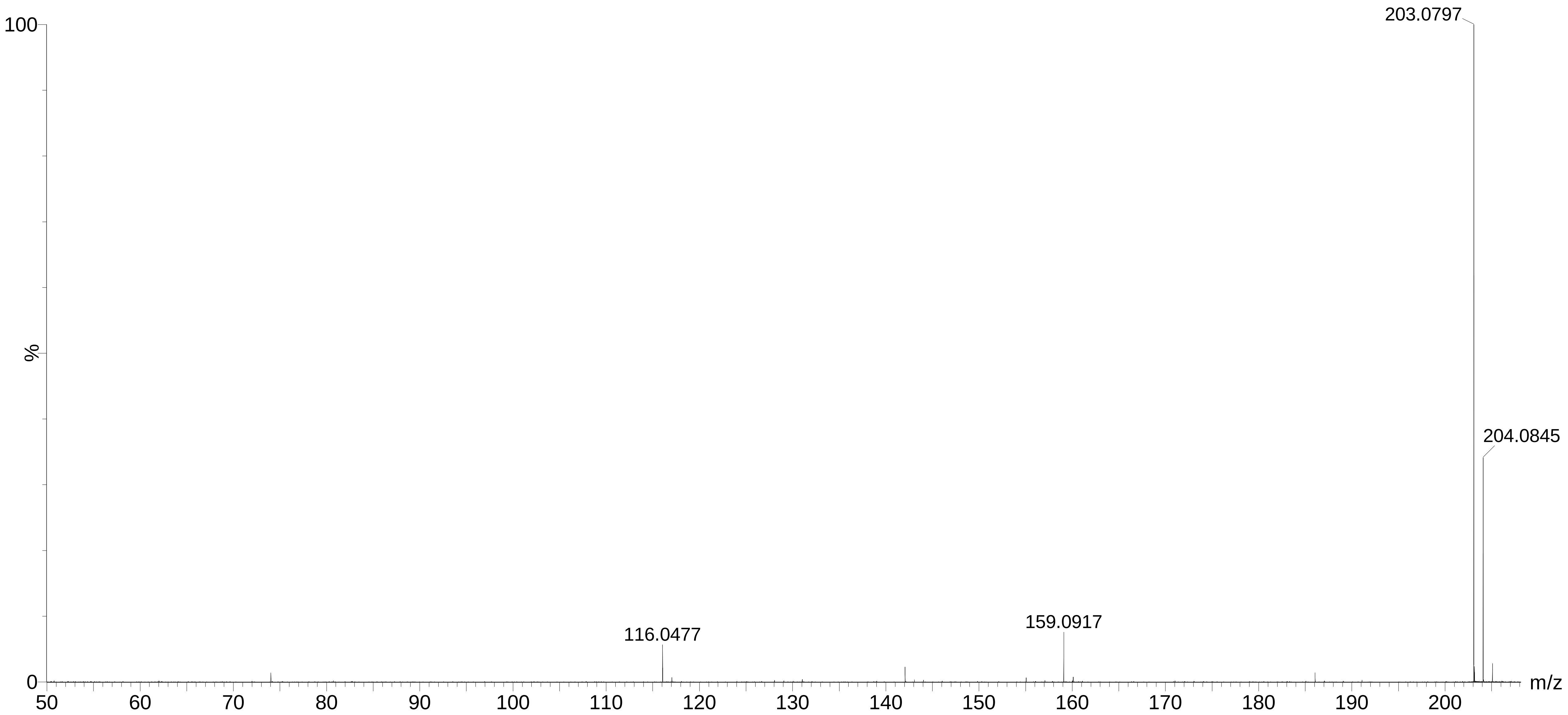

Sinapic Acid

Sinapic Acid-POS

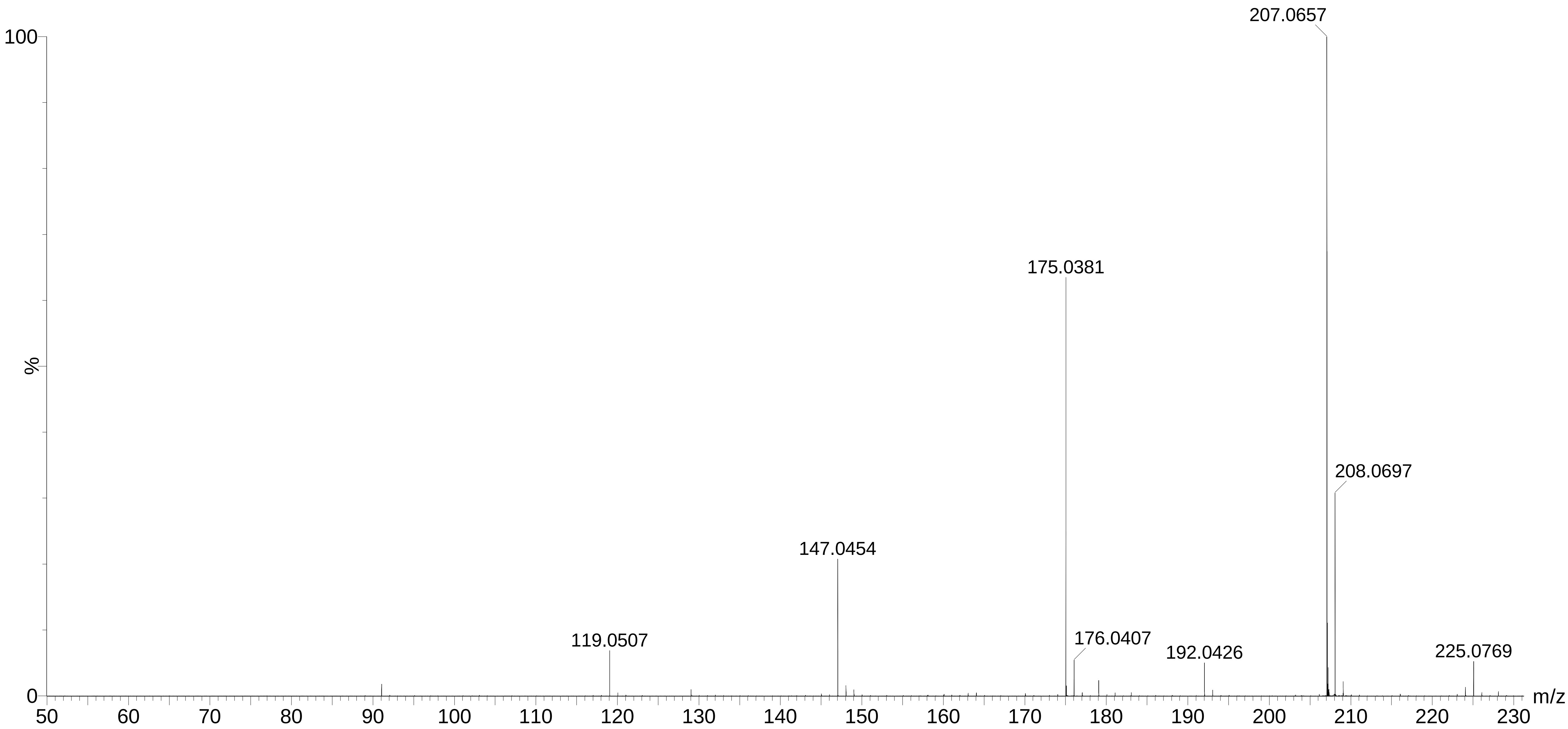

Sinapic Acid-NEG

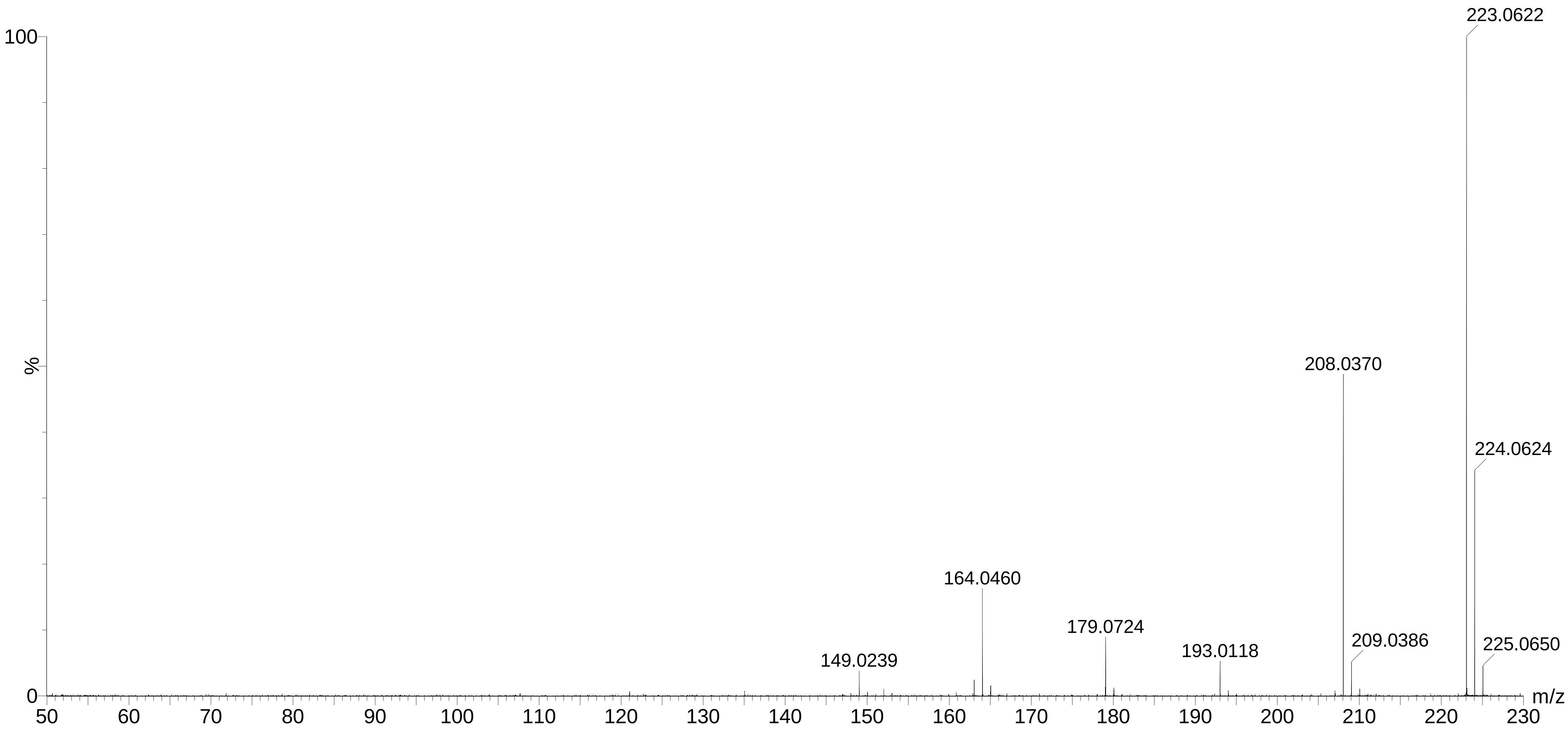

2-O-alpha-D-Glucopyranosyl-L-ascorbic acid

2-O-alpha-D-Glucopyranosyl-L-ascorbic acid-POS

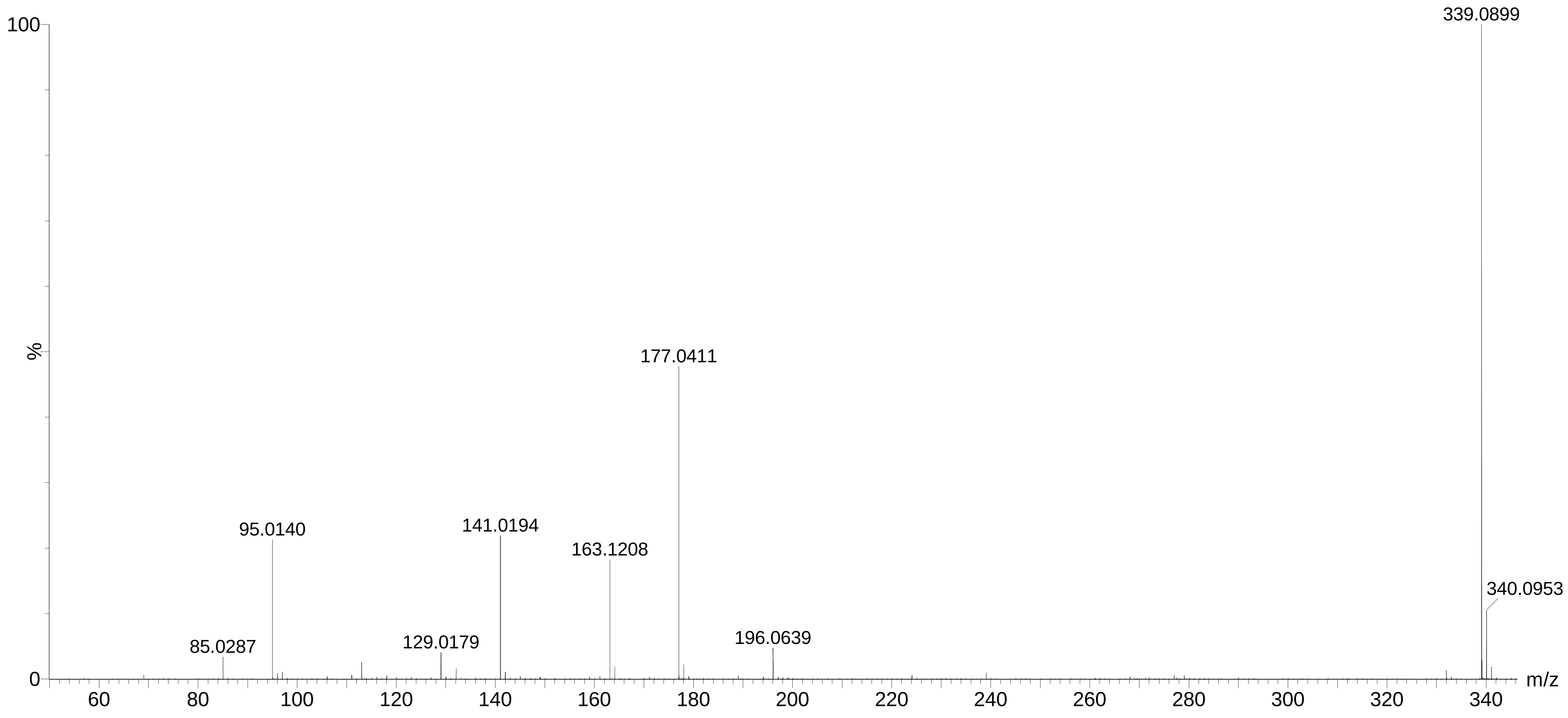

2-O-alpha-D-Glucopyranosyl-L-ascorbic acid-NEG

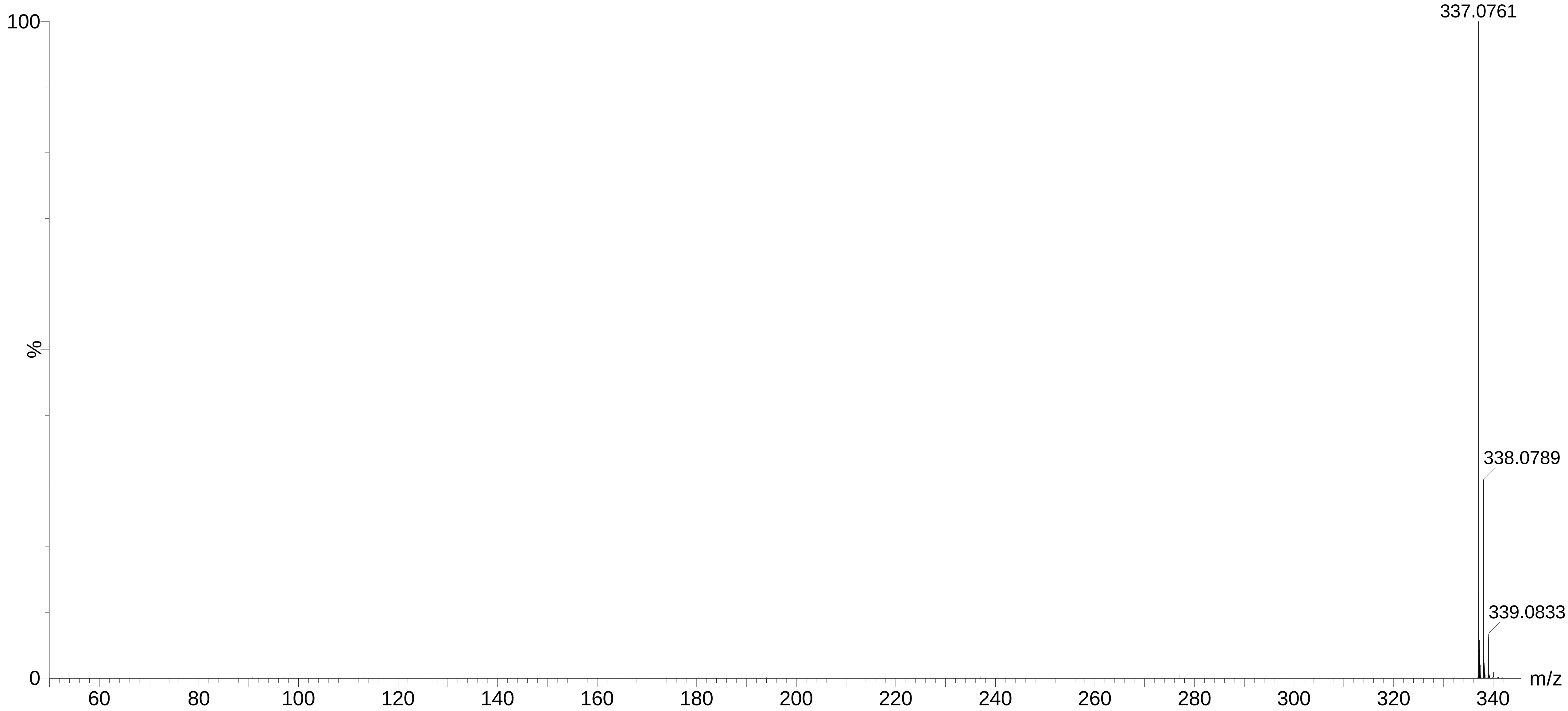

Protocatechuic acid 3-glucoside

Protocatechuic acid 3-glucoside-POS

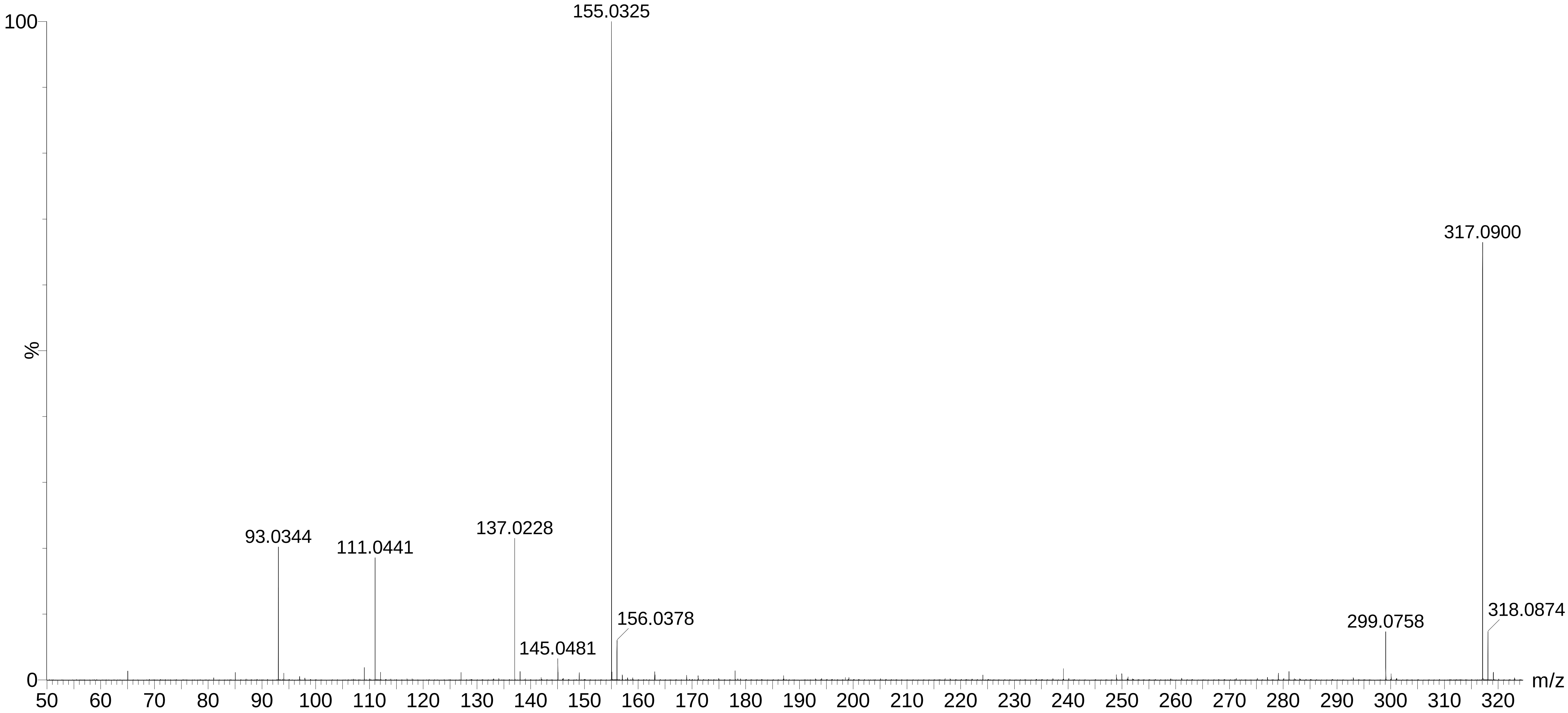

Protocatechuic acid 3-glucoside-NEG

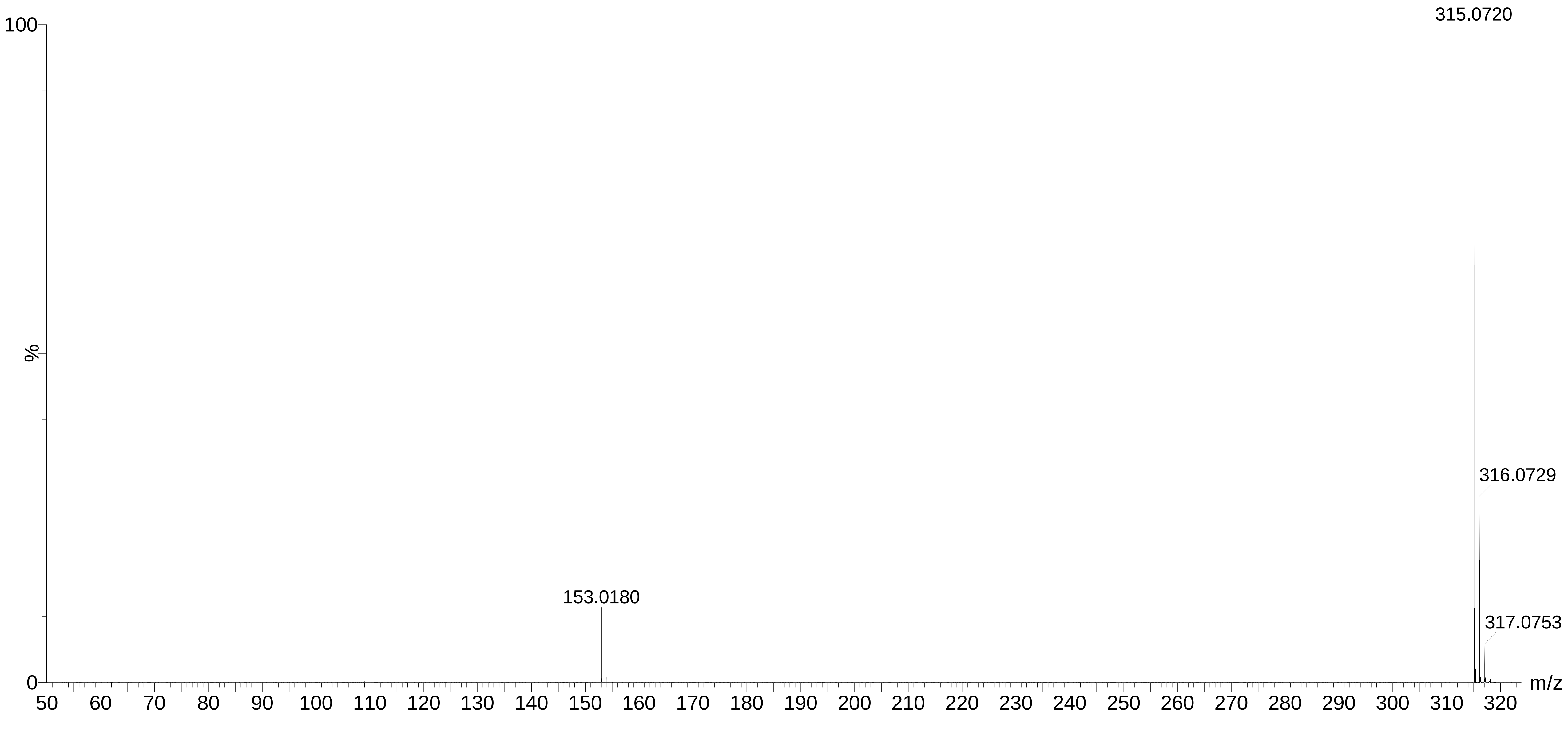

Baimaside

Baimaside-POS

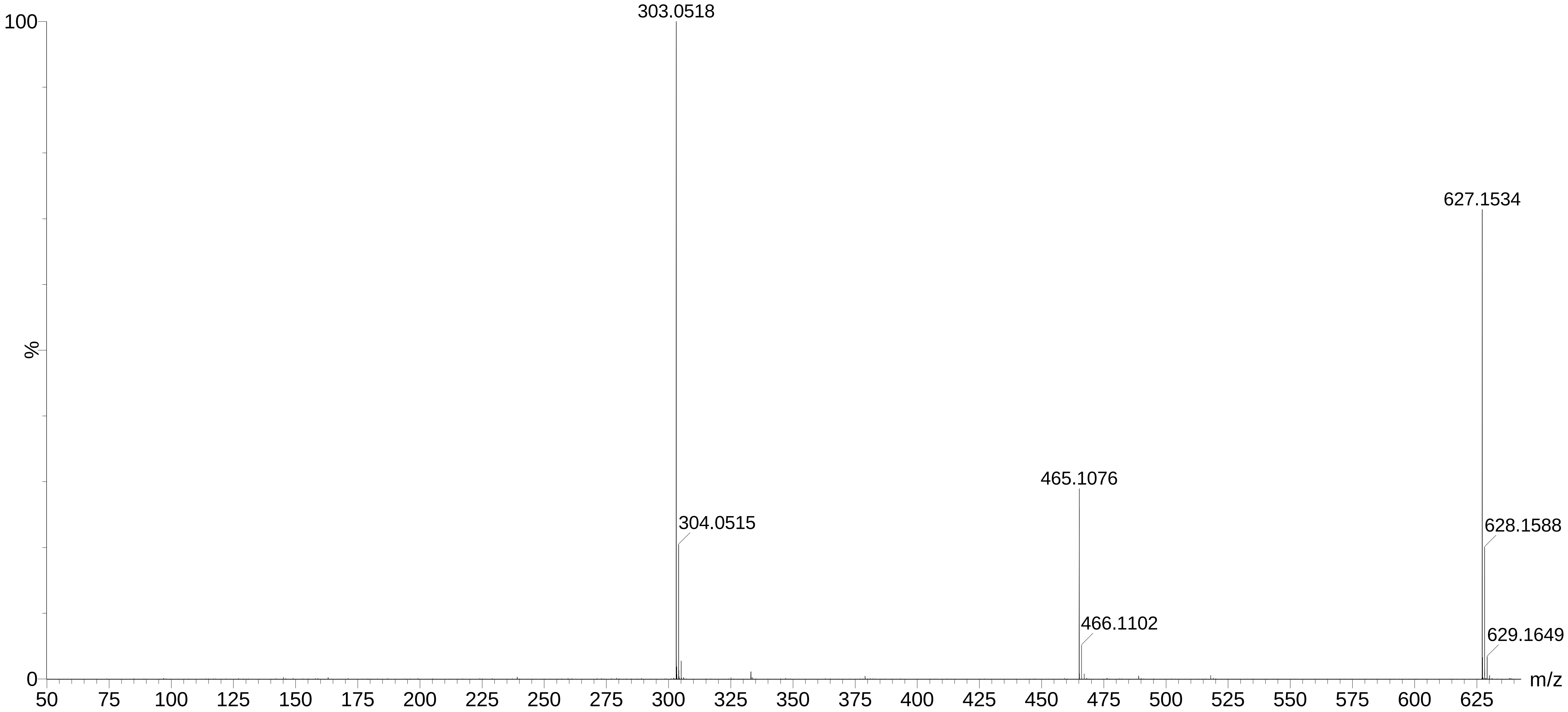

Baimaside-NEG

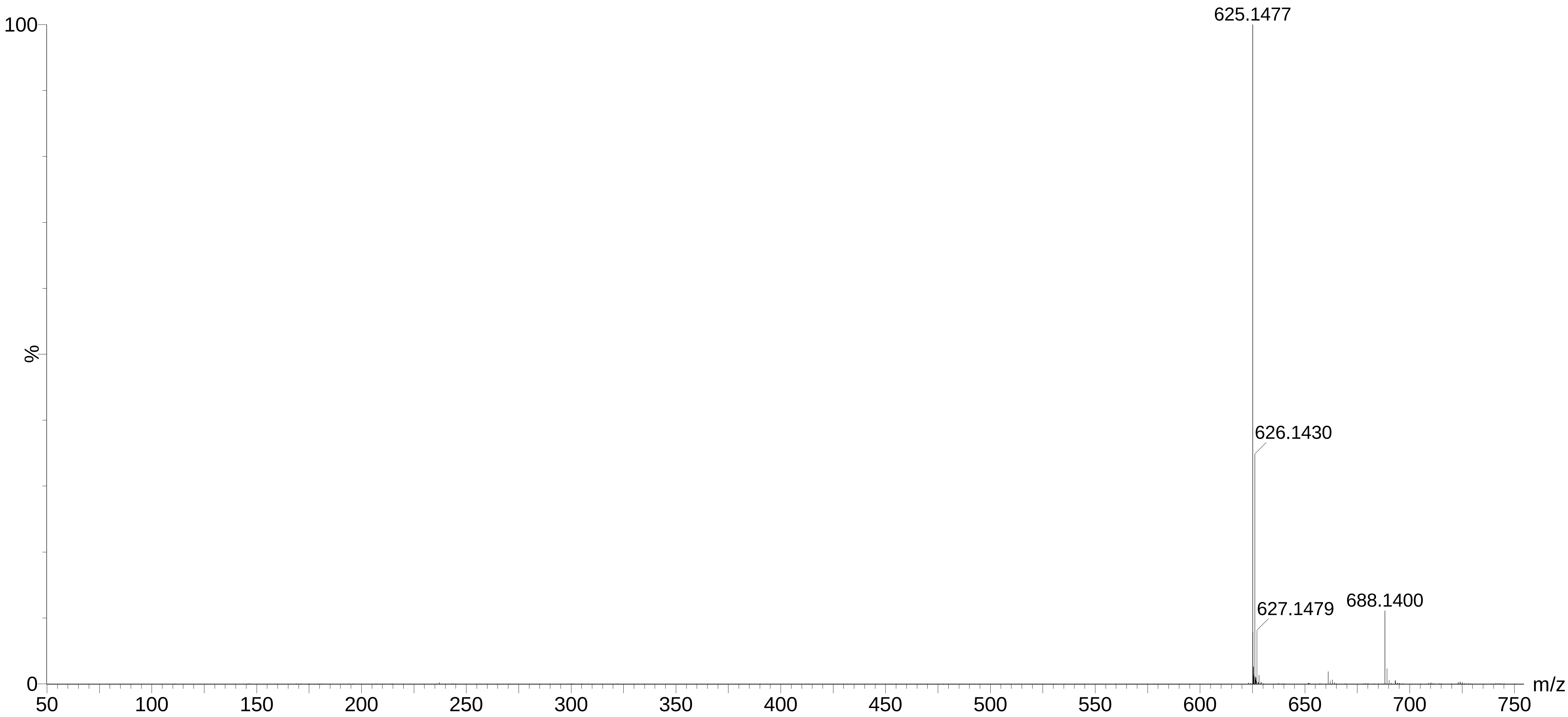

1-o-caffeoylglucose

1-o-caffeoylglucose-POS

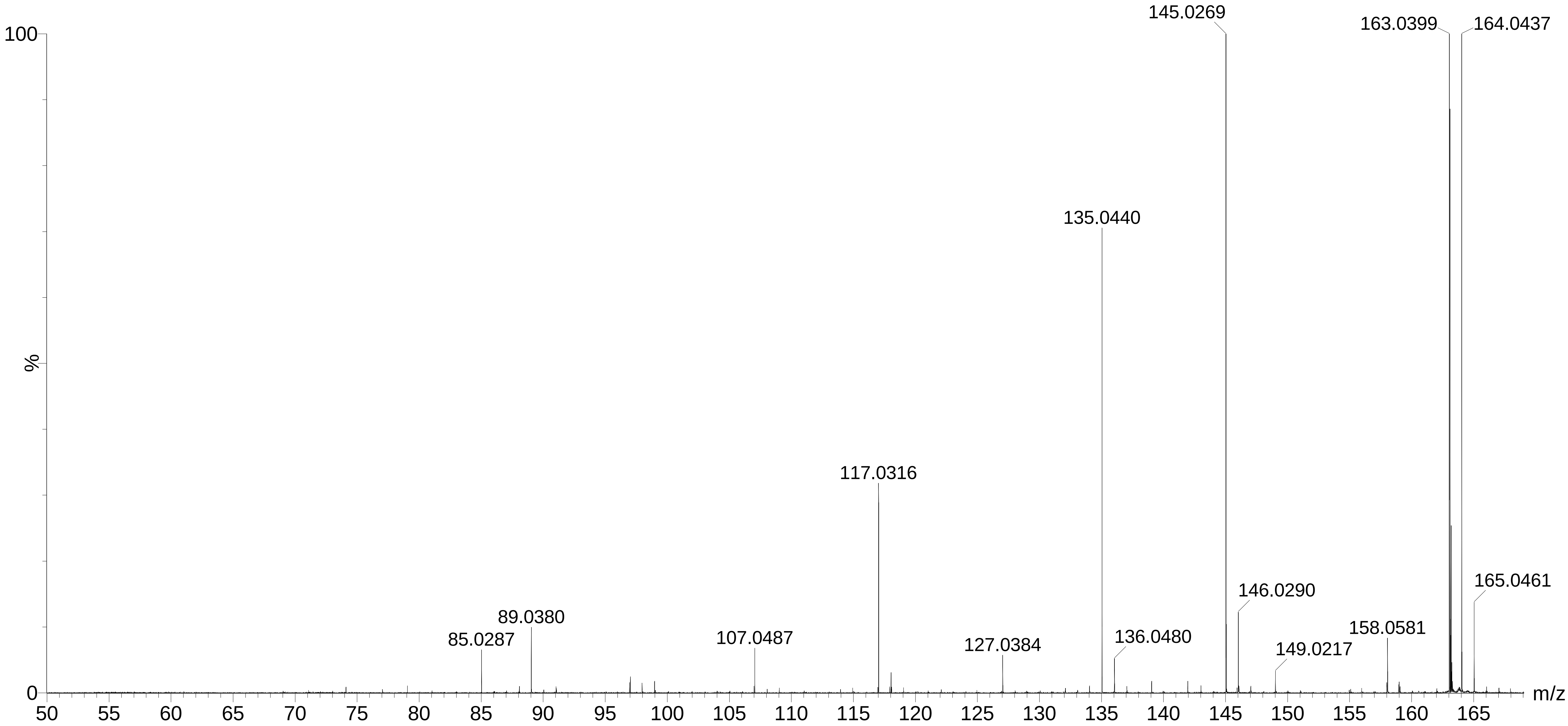

1-o-caffeoylglucose-NEG

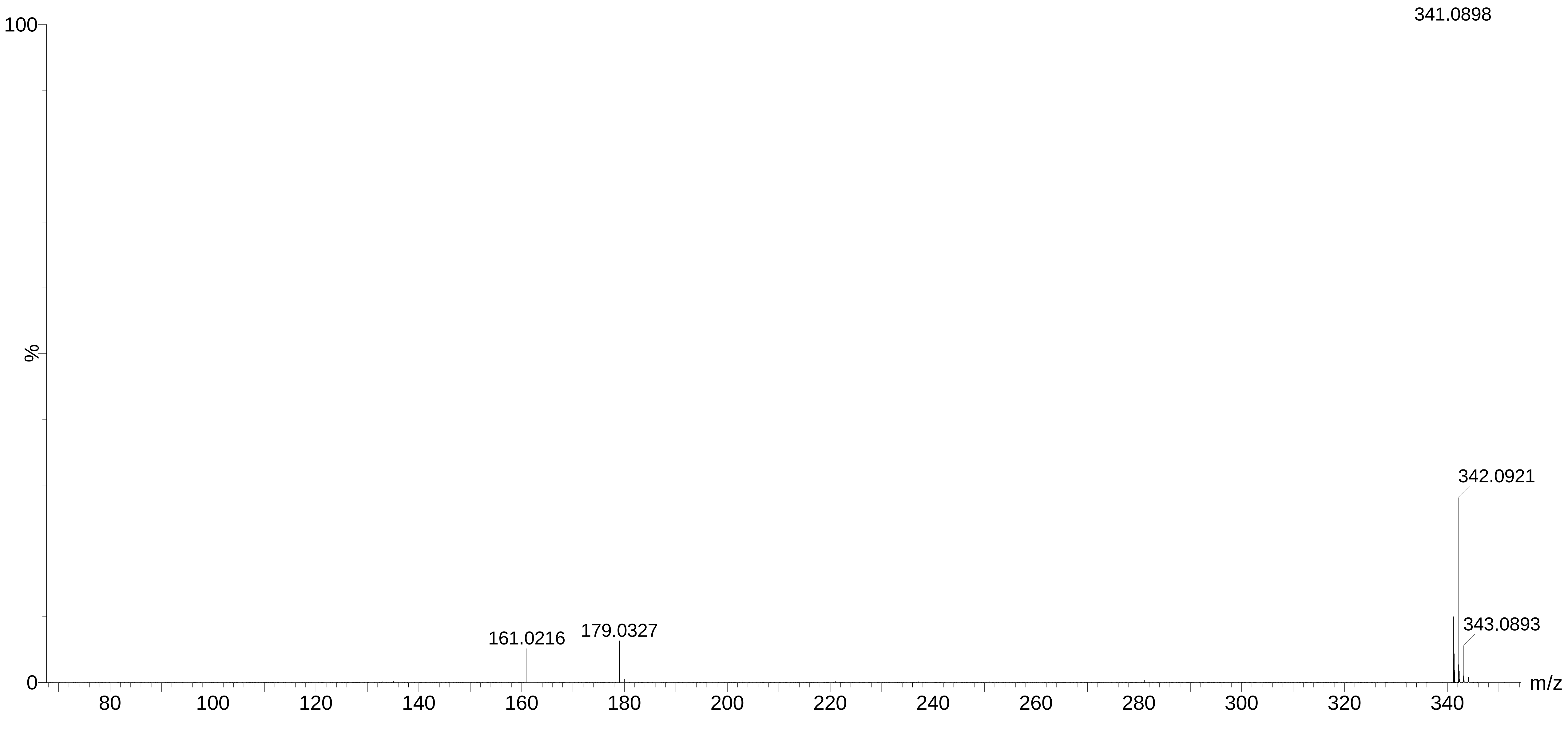

Catechin

Catechin-POS

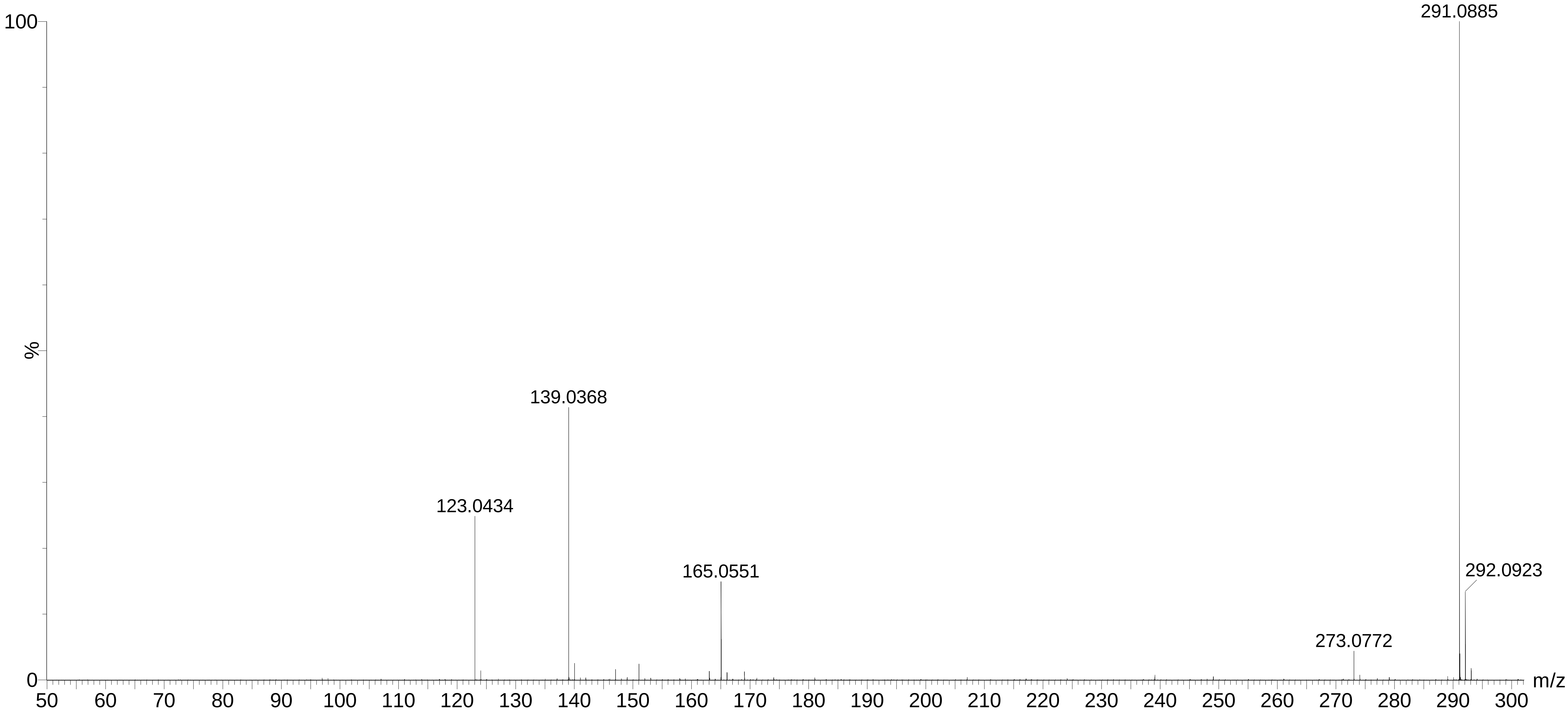

Catechin-NEG

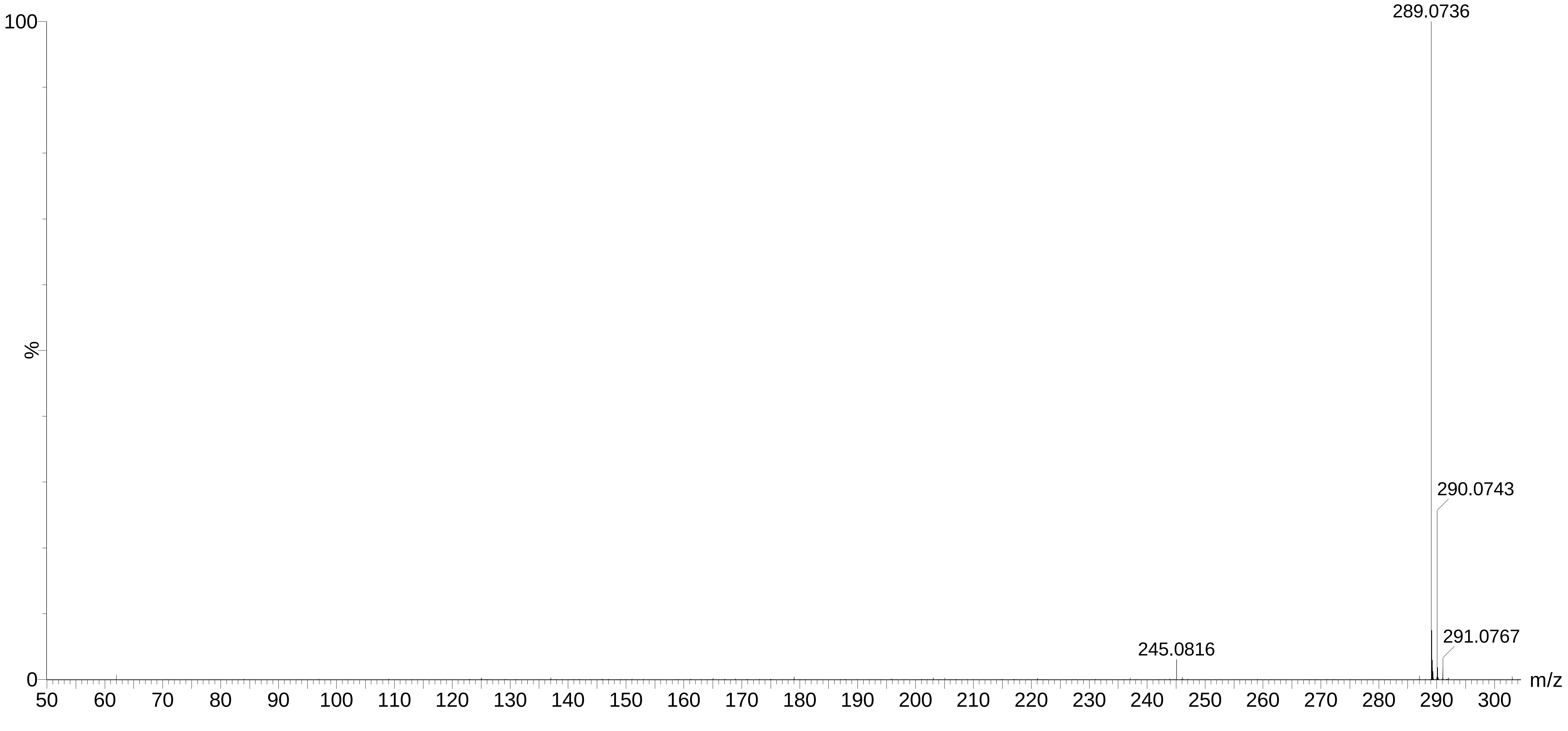

Catechin-NEG

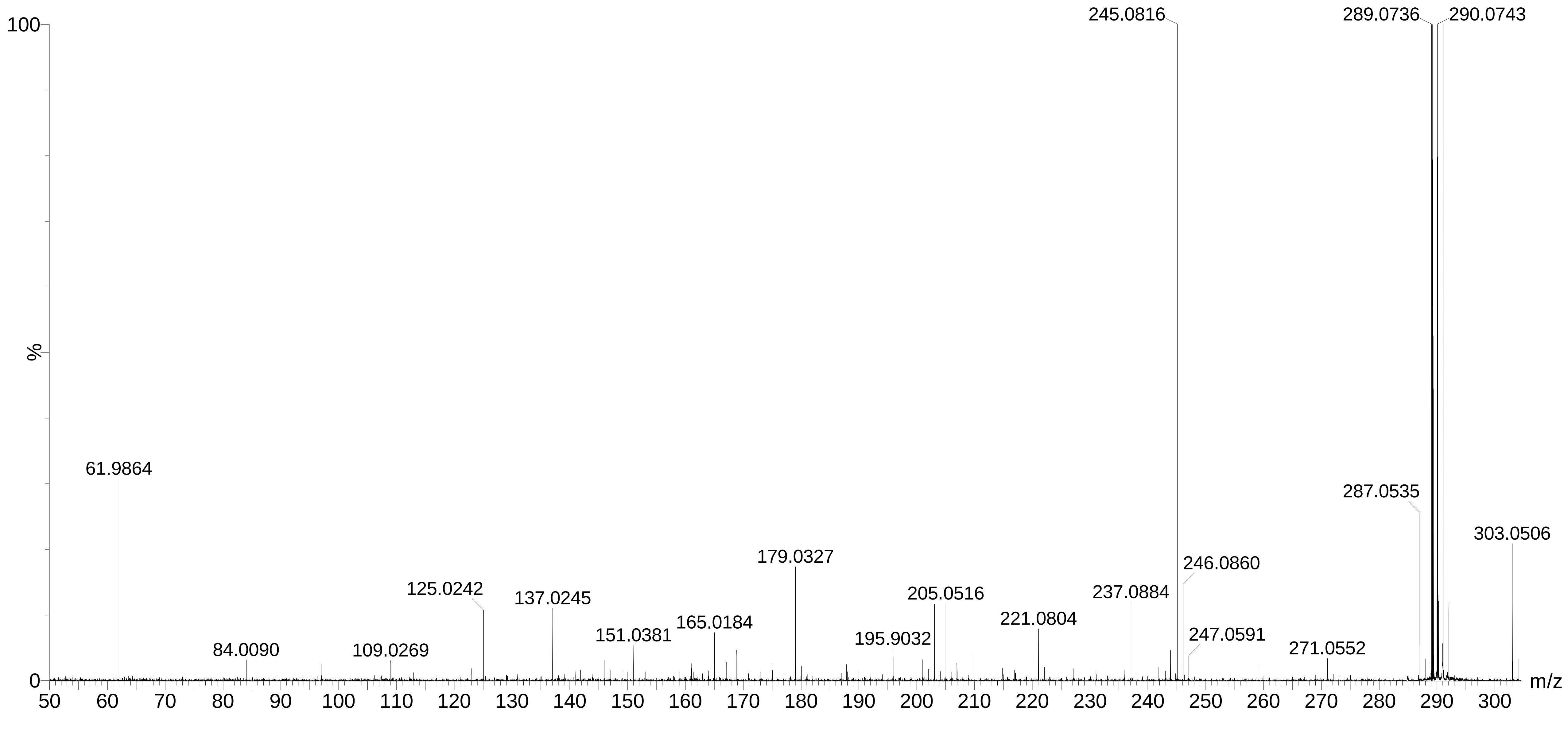

Hyperoside

Hyperoside-POS

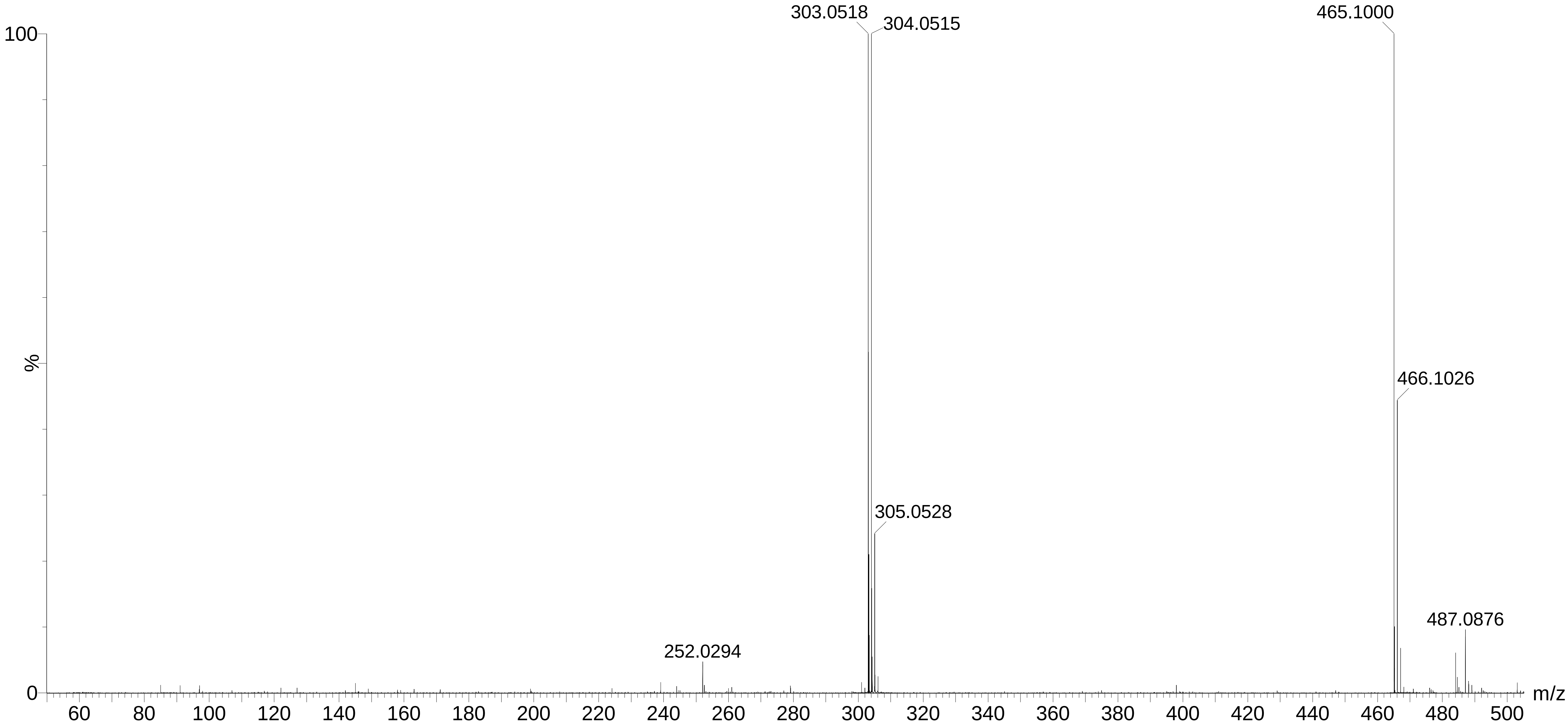

Hyperoside-POS

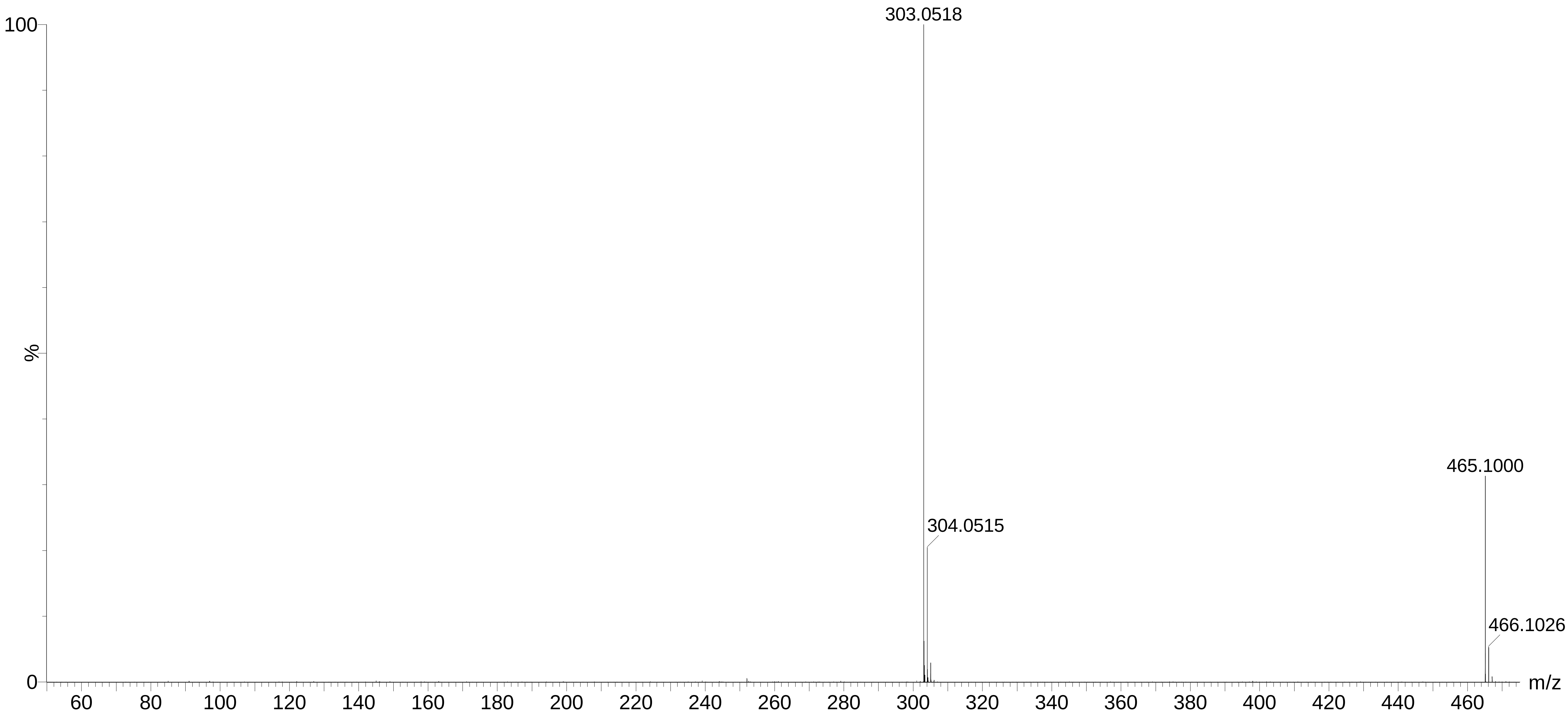

Phenylalanine

Phenylalanine-POS

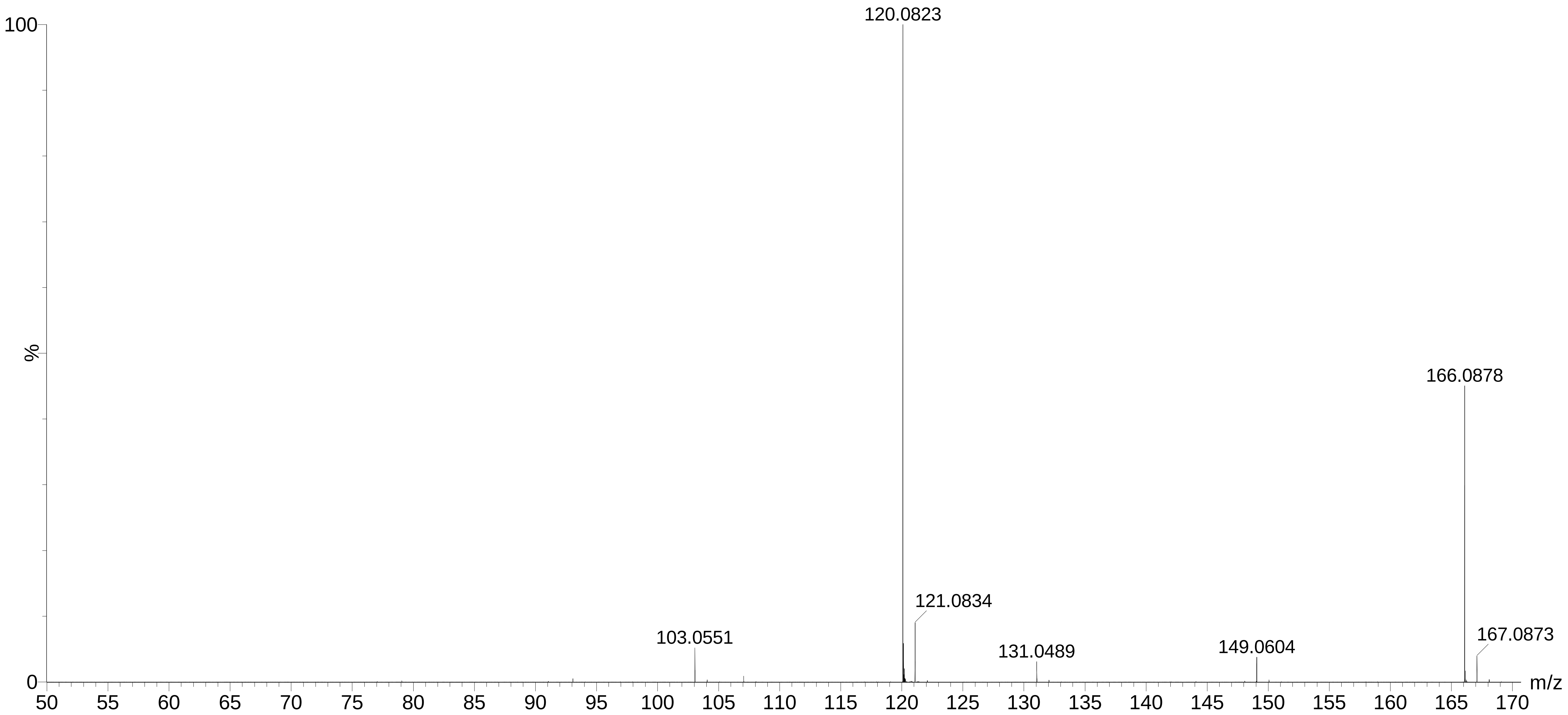

Phenylalanine-NEG

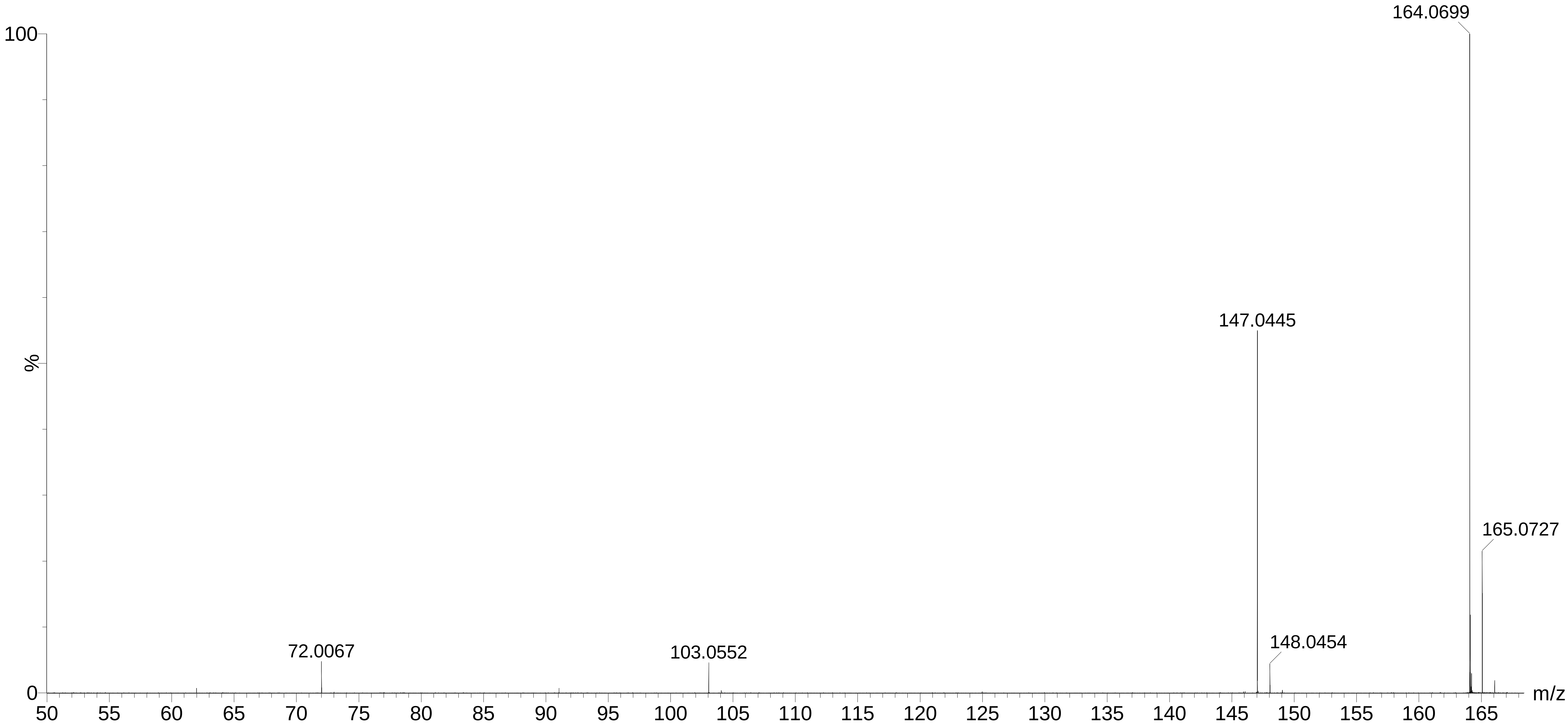

Procyanidin B1

Procyanidin B1-POS

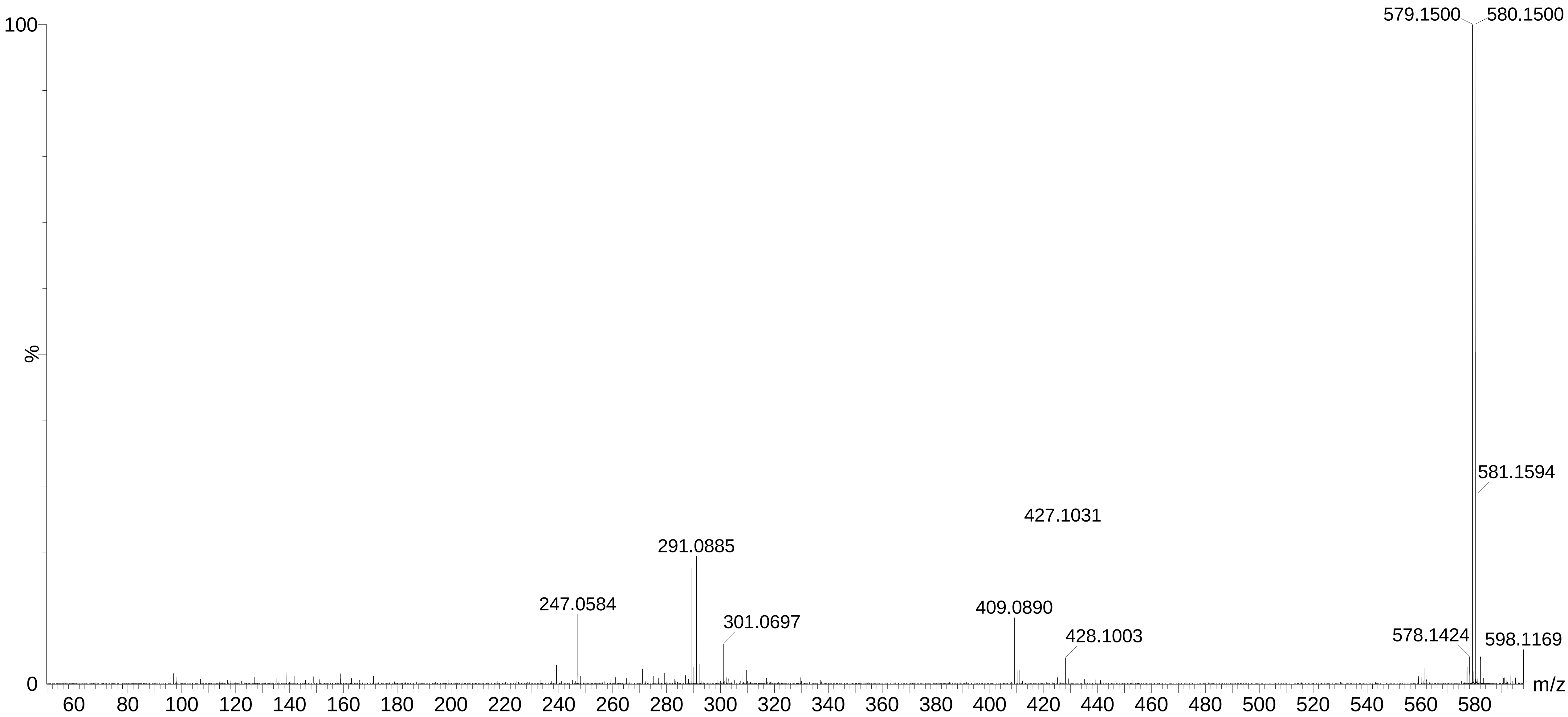

Procyanidin B1-NEG

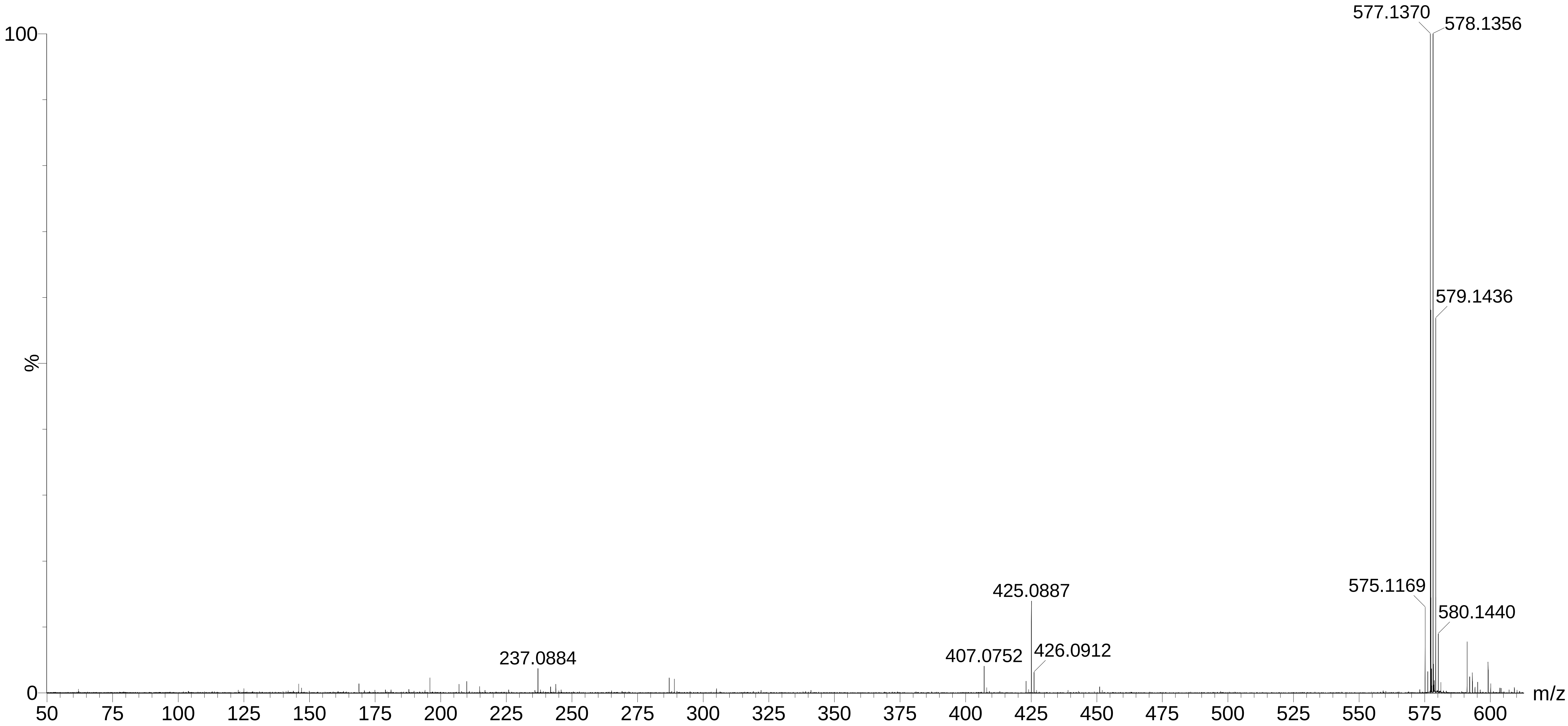

7-Methoxy-2H-chromen-2-one

7-Methoxy-2H-chromen-2-one-POS

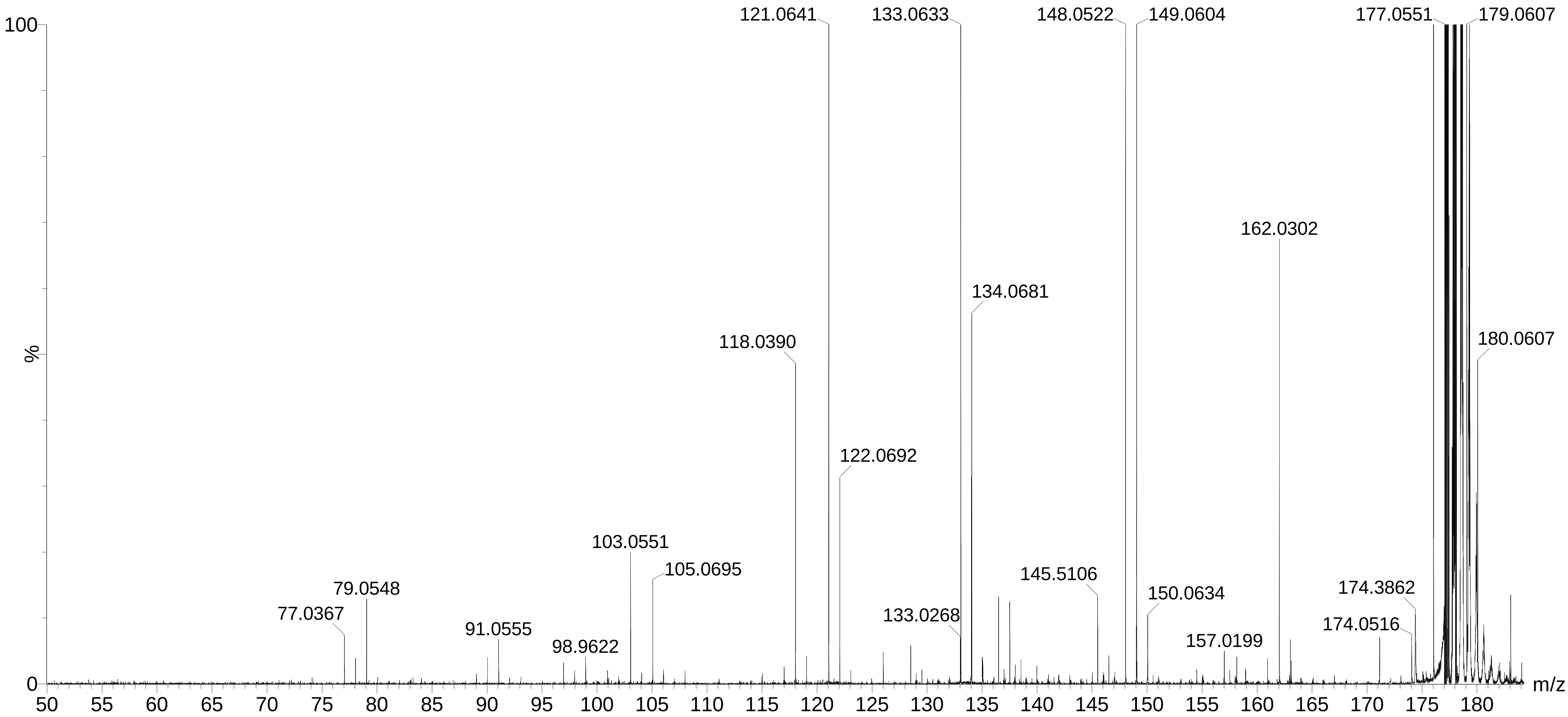

1-Monolinolein

1-Monolinolein-POS

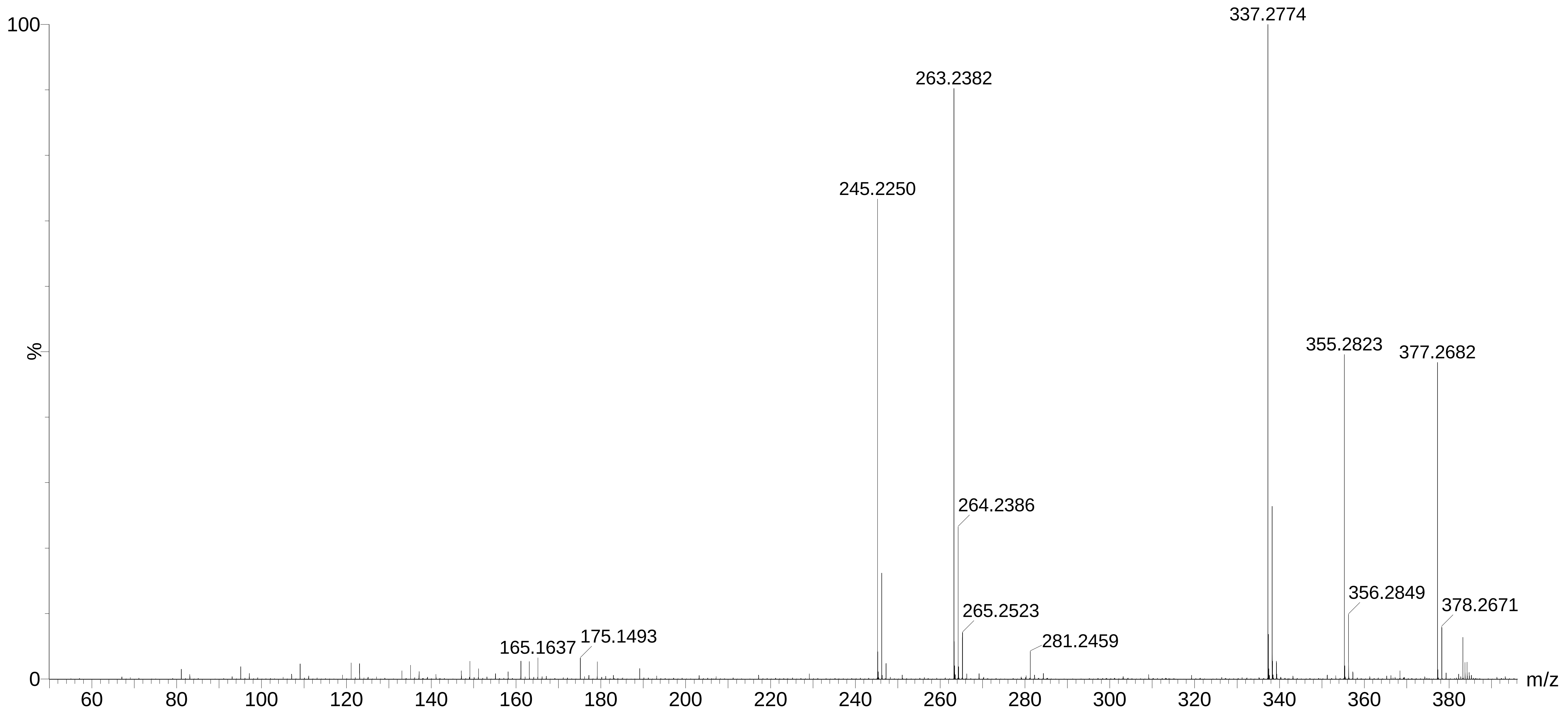

1-Monolinolein-POS

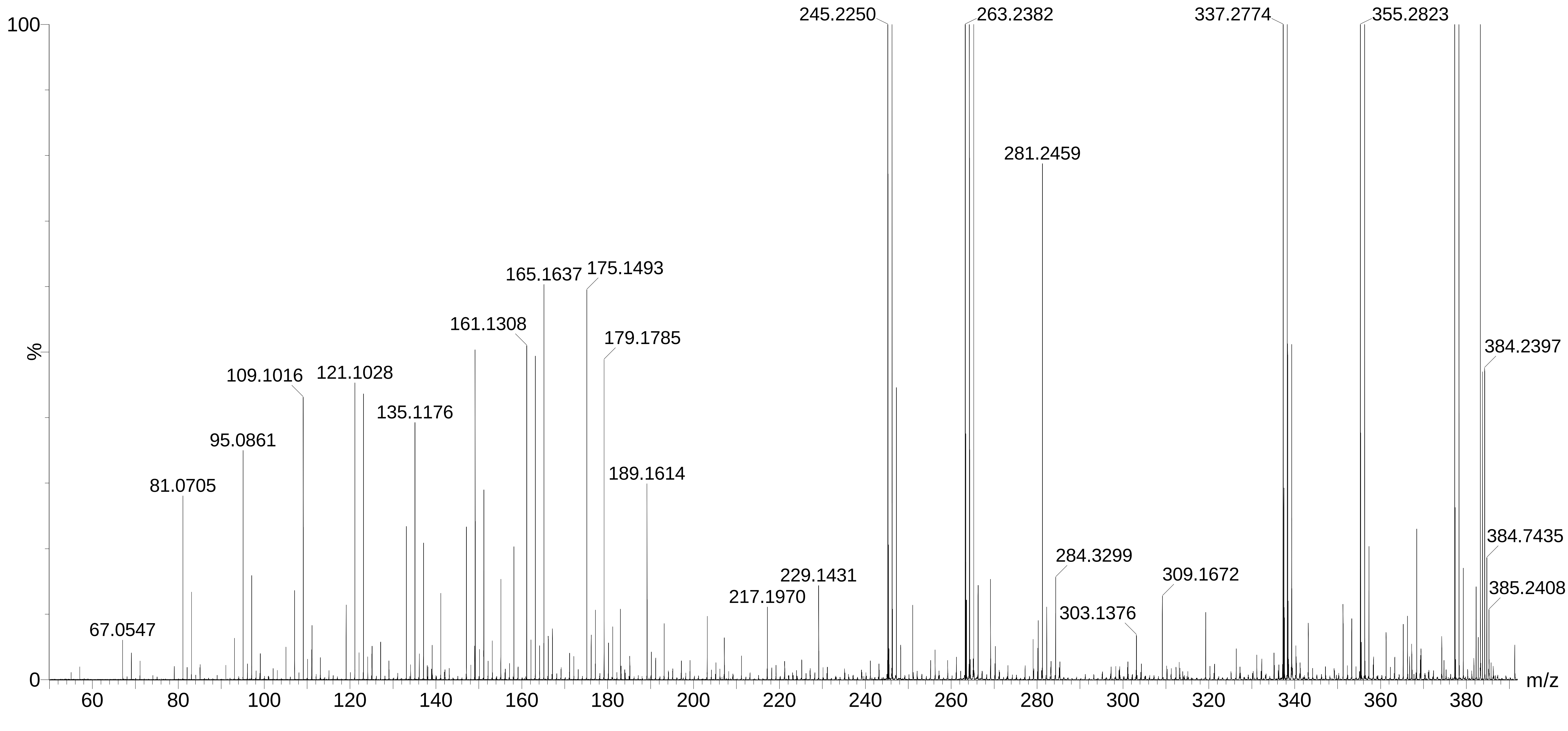

1-Monolinolein-NEG

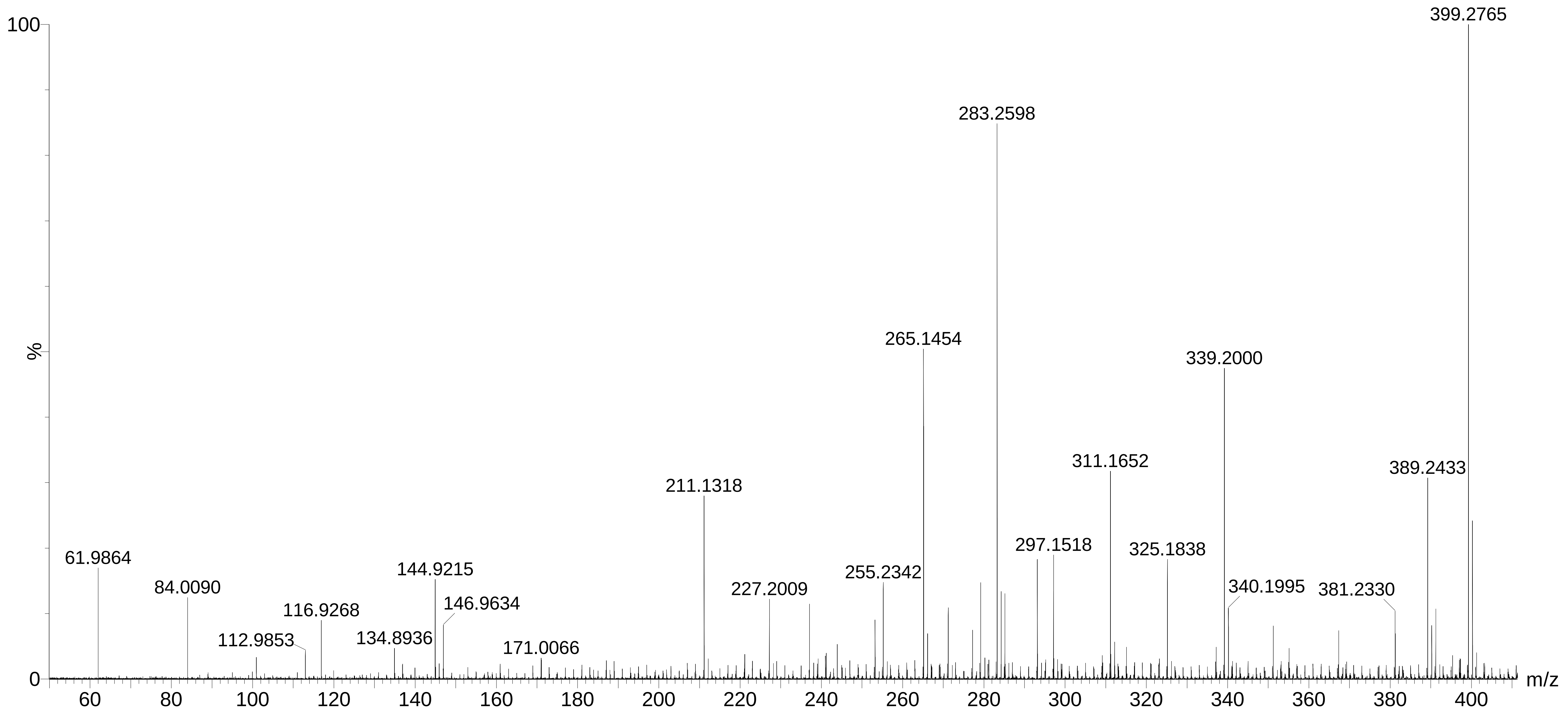

Supplement: Supplementary file 1 [file molecules-31-02614-s001.zip › Supplementary Figure S1. The MSMS spectra of reference standards.pdf]
