# Supplementary material for: Chemical Profiling and Differential Constituents Analysis of Honghema and Its Easily Confusable Species Driven by High-Resolution Mass Spectrometry and Pattern Recognition
Source: Molecules. 2026 Jul 27;31(15):2614. doi: 10.3390/molecules31152614 (PMC13468369; doi:10.3390/molecules31152614)
Supplement: Supplementary file 1 [file molecules-31-02614-s001.zip › Supplementary Table S1. Experimental sample information.pdf]

Supplementary Table S1. Experimental sample information.

Serial number	Species name	Collecting locations	Collecting date
L1	*Laportea bulbifera*	Nayong County, Bijie City, Guizhou Province	2024/12/03
L2	*Laportea bulbifera*	Nayong County, Bijie City, Guizhou Province	2024/12/03
L3	*Laportea bulbifera*	Nayong County, Bijie City, Guizhou Province	2024/12/03
L4	*Laportea bulbifera*	Nayong County, Bijie City, Guizhou Province	2024/12/03
L5	*Laportea bulbifera*	Nayong County, Bijie City, Guizhou Province	2024/12/03
L6	*Laportea bulbifera*	Nayong County, Bijie City, Guizhou Province	2024/12/03
G1	*Girardinia diversifolia*	Qianxi County, Bijie City, Guizhou Provinc	2024/11/09

G2	*Girardinia diversifolia*	Qianxi County, Bijie City, Guizhou Province	2024/11/09
G3	*Girardinia diversifolia*	Qianxi County, Bijie City, Guizhou Province	2024/11/09
G4	*Girardinia diversifolia*	Qianxi County, Bijie City, Guizhou Province	2024/11/09
G5	*Girardinia diversifolia*	Qianxi County, Bijie City, Guizhou Province	2024/11/09
G6	*Girardinia diversifolia*	Qianxi County, Bijie City, Guizhou Province	2024/11/09
U1	*Urtica mairei*	Qianxi County, Bijie City, Guizhou Province	2024/11/09
U2	*Urtica mairei*	Qianxi County, Bijie City, Guizhou Province	2024/11/09
U3	*Urtica mairei*	Qianxi County, Bijie City, Guizhou Province	2024/11/09
U4	*Urtica mairei*	Qianxi County, Bijie City, Guizhou	2024/11/09

		Province	
U5	*Urtica mairei*	Qianxi County, Bijie City, Guizhou Province	2024/11/09
U6	*Urtica mairei*	Qianxi County, Bijie City, Guizhou Province	2024/11/09
