# Supplementary material for: Chemical Profiling and Differential Constituents Analysis of Honghema and Its Easily Confusable Species Driven by High-Resolution Mass Spectrometry and Pattern Recognition
Source: Molecules. 2026 Jul 27;31(15):2614. doi: 10.3390/molecules31152614 (PMC13468369; doi:10.3390/molecules31152614)
Supplement: Supplementary file 1 [file molecules-31-02614-s001.zip › Supplementary Table S2. The Standard compounds for experiments.pdf]

Supplementary Table S2
The Standard compounds for experiments.

NO.	Compounds	CAS	Formula	Purities	Lot number	Manufacturers
1	**Rutin**	153-18-4	$C_{27}H_{30}O_{16}$	⩾98%	200506010	Beijing Beite Renkang Biopharmaceutical Technology Co., Ltd.
2	**Luteolin**	491-70-3	$C_{15}H_{10}O_6$	⩾98%	BT-JG02	Beijing Beite Renkang Biopharmaceutical Technology Co., Ltd.
3	**9-Ribofuranosyladenine**	58-61-7	$C_{10}H_{13}N_5O_4$	⩾98%	200507003	Beijing Beite Renkang Biopharmaceutical Technology Co., Ltd.
4	**Quercetin-3-*O*-beta-D-glucose-7-*O*-beta-D-gentiobioside**	60778-02-1	$C_{33}H_{40}O_{22}$	⩾98%	200507049	Beijing Beite Renkang Biopharmaceutical Technology Co., Ltd.
5	**Chlorogenic acid**	327-97-9	$C_{16}H_{18}O_9$	⩾98%	210203001	Beijing Beite Renkang Biopharmaceutical Technology Co., Ltd.
6	**Kaempferol**	520-18-3	$C_{15}H_{10}O_6$	⩾98%	24071503	Beijing Beite Renkang Biopharmaceutical Technology Co., Ltd.
7	**Citric acid**	77-92-9	$C_6H_8O_7$	⩾98%	24110236	Beijing Beite Renkang Biopharmaceutical Technology Co., Ltd.
8	**Cryptochlorogenic acid**	905-99-7	$C_{16}H_{18}O_9$	⩾98%	200507025	Beijing Beite Renkang Biopharmaceutical Technology Co., Ltd.
9	**Ferulic acid**	1135-24-6	$C_{10}H_{10}O_4$	⩾98%	24030102	Beijing Beite Renkang Biopharmaceutical Technology Co., Ltd.
10	**Tryptophan**	73-22-3	$C_{11}H_{12}N_2O_2$	⩾98%	200506013	Beijing Beite Renkang Biopharmaceutical Technology Co., Ltd.

NO.	Compounds	CAS	Formula	Purities	Lot number	Manufacturers
11	**Sinapic Acid**	7362-37-0	$C_{11}H_{12}O_5$	⩾98%	200507054	Beijing Beite Renkang Biopharmaceutical Technology Co., Ltd.
12	**2-*O*-alpha-D-Glucopyranosyl-L-ascorbic acid**	129499-78-1	$C_{12}H_{18}O_{11}$	⩾98%	26011102	Beijing Beite Renkang Biopharmaceutical Technology Co., Ltd.
13	**Protocatechuic acid 3-glucoside**	20300-54-3	$C_{13}H_{16}O_9$	⩾98%	25120815	Beijing Beite Renkang Biopharmaceutical Technology Co., Ltd.
14	**Baimaside**	18609-17-1	$C_{27}H_{30}O_{17}$	⩾98%	26012010	Beijing Beite Renkang Biopharmaceutical Technology Co., Ltd.
15	**1-*O*-caffeoylglucose**	14364-08-0	$C_{15}H_{18}O_9$	⩾98%	26010344	Beijing Beite Renkang Biopharmaceutical Technology Co., Ltd.
16	**Catechin**	154-23-4	$C_{15}H_{14}O_6$	⩾98%	25121011	Beijing Beite Renkang Biopharmaceutical Technology Co., Ltd.
17	**Hyperoside**	482-36-0	$C_{21}H_{20}O_{12}$	⩾98%	25100103	Beijing Beite Renkang Biopharmaceutical Technology Co., Ltd.
18	**Phenylalanine**	63-91-2	$C_9H_{11}NO_2$	⩾98%	25042338	Beijing Beite Renkang Biopharmaceutical Technology Co., Ltd.
19	**Procyanidin B1**	20315-25-7	$C_{30}H_{26}O_{12}$	⩾98%	25030619	Beijing Beite Renkang Biopharmaceutical Technology Co., Ltd.
20	**7-Methoxy-2H-chromen-2-one**	531-59-9	$C_{10}H_8O_3$	⩾98%	25091001	Beijing Beite Renkang Biopharmaceutical Technology Co., Ltd.
21	**1-Monolinolein**	2277-28-3	$C_{21}H_{38}O_4$	⩾98%	26020187	Beijing Beite Renkang Biopharmaceutical Technology Co., Ltd.
